# Identifying susceptibility of children and adolescents to the Omicron variant (B.1.1.529)

**DOI:** 10.1186/s12916-022-02655-z

**Published:** 2022-11-23

**Authors:** June Young Chun, Hwichang Jeong, Yongdai Kim

**Affiliations:** 1 Department of Internal Medicine, National Cancer Center, Goyang, South Korea; 2 Department of Statistics, Seoul National University, 56-1 Mountain, Sillim-dong, Gwanak-gu, Seoul, 08826 South Korea

**Keywords:** SARS-CoV-2, COVID-19, B.1.1.529 SARS-CoV-2 variant, Mathematical model, Bayesian analysis, Child, Adolescent, Susceptibility

## Abstract

**Background:**

The Omicron variant (B.1.1.529) is estimated to be more transmissible than previous strains of SARS-CoV-2 especially among children, potentially resulting in croup which is a characteristic disease in children. Current coronavirus disease 2019 (COVID-19) cases among children might be higher because (i) school-aged children have higher contact rates and (ii) the COVID-19 vaccination strategy prioritizes the elderly in most countries. However, there have been no reports confirming the age-varying susceptibility to the Omicron variant to date.

**Methods:**

We developed an age-structured compartmental model, combining age-specific contact matrix in South Korea and observed distribution of periods between each stage of infection in the national epidemiological investigation. A Bayesian inference method was used to estimate the age-specific force of infection and, accordingly, age-specific susceptibility, given epidemic data during the third (pre-Delta), fourth (Delta driven), and fifth (Omicron driven) waves in South Korea. As vaccine uptake increased, individuals who were vaccinated were excluded from the susceptible population in accordance with vaccine effectiveness against the Delta and Omicron variants, respectively.

**Results:**

A significant difference between the age-specific susceptibility to the Omicron and that to the pre-Omicron variants was found in the younger age group. The rise in susceptibility to the Omicron/pre-Delta variant was highest in the 10–15 years age group (5.28 times [95% CI, 4.94–5.60]), and the rise in susceptibility to the Omicron/Delta variant was highest in the 15–19 years age group (3.21 times [95% CI, 3.12–3.31]), whereas in those aged 50 years or more, the susceptibility to the Omicron/pre-Omicron remained stable at approximately twofold.

**Conclusions:**

Even after adjusting for contact pattern, vaccination status, and waning of vaccine effectiveness, the Omicron variant of SARS-CoV-2 tends to propagate more easily among children than the pre-Omicron strains.

**Supplementary Information:**

The online version contains supplementary material available at 10.1186/s12916-022-02655-z.

## Background

As the Omicron variant (B.1.1.529) of SARS-CoV-2 drives a new surge in coronavirus disease 2019 (COVID-19) cases globally, increasing proportion of pediatric cases is noteworthy [1]. In the United States (US), where the Omicron variant had been predominant since December 2021, the seroprevalence of infection-induced SARS-CoV-2 antibodies among children aged 0–11 years increased from 44.2 to 75.2% during December 2021 to February 2022, recording the highest increase among all age groups (overall US seroprevalence increased from 33.5 to 57.7%) [2]. In England, pediatric admissions with COVID-19 infections began to rise since December 26, 2021, from an average of 40 admissions per day to 120 per day, a 3-fold rise in 2 weeks [3].

The rise in pediatric cases might be attributed to the elderly-prioritized vaccination strategy against COVID-19 and relatively higher contact rates among school-aged children than adults. As of May 31, 2022, the World Health Organization (WHO) authorized COVID-19 vaccines for individuals aged 18 years and older, and only one vaccine could be used for individuals from 5 years of age, which let children more vulnerable to COVID-19 [4]. Otherwise, children might genuinely be more susceptible to contracting the Omicron infections than adults. Identifying the age-specific susceptibility to SARS-CoV-2 is of much interest for effective public health strategies and vaccination policy. However, it is often not easy to clarify the age-specific susceptibility to an infection due to the lack of sufficient data.

**Fig. 1:**
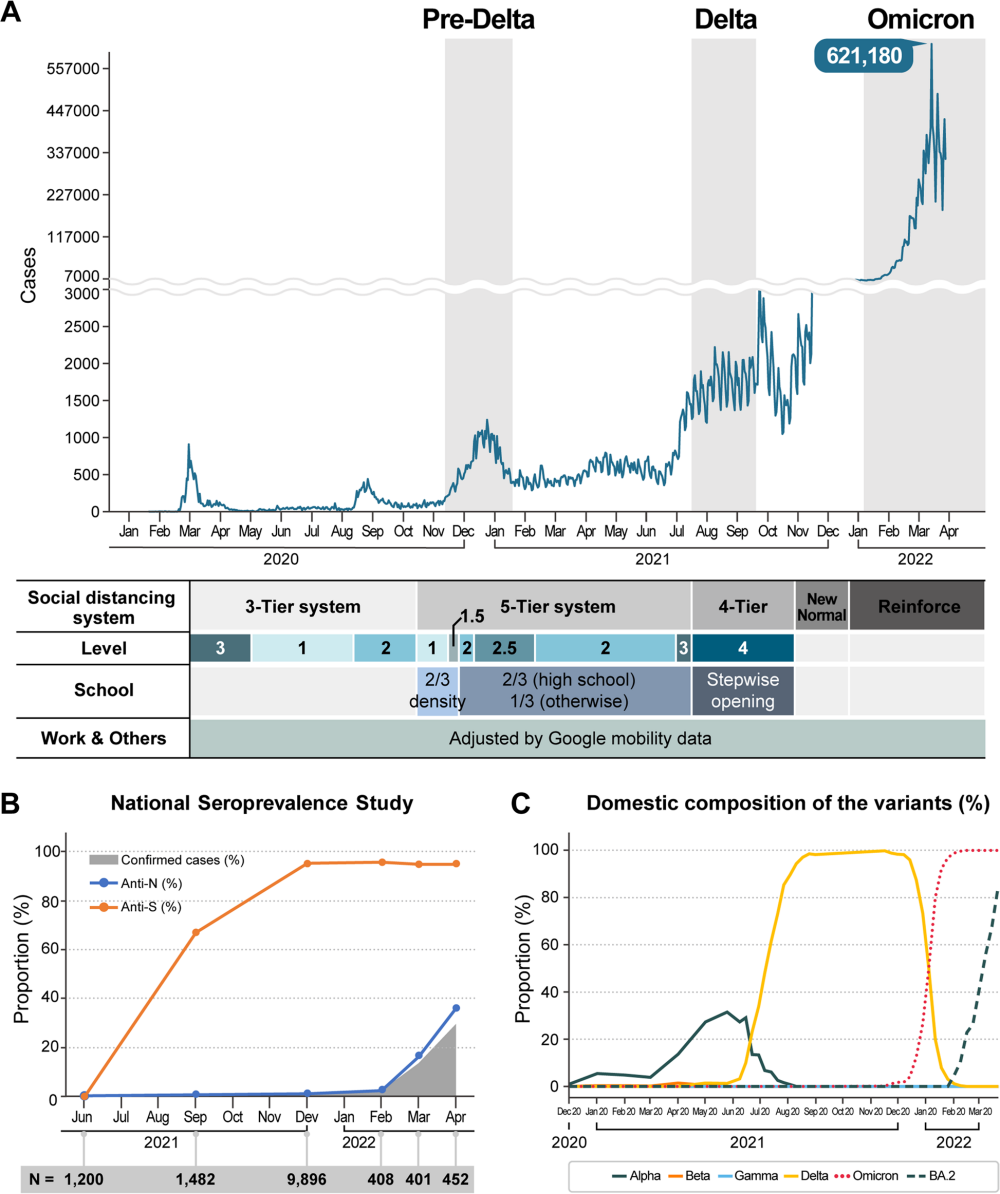
Characteristics of the SARS-CoV-2 outbreak in South Korea: **A** epidemic curve and national interventions, **B** national seroprevalence data*, and **C** domestic composition of variant strains during the study period. *The seroprevalence of SARS-CoV-2-specific anti-nucleocapsid (anti-*N*) and anti-spike (anti-*S*) antibodies have been studied in samples from the Korea National Health and Nutrition Examination Survey which included participants aged 10 years or more. The seroprevalence study was performed in samples from blood donors (aged 20 to 59 years) in December 2021, only. The number denotes the sample size of each study

In South Korea, the biggest 5th wave has been driven by the Omicron variant since January 2022, following the 4th wave by the Delta variant and the 3rd wave by the original SARS-CoV-2 virus (Fig. 1A). Here, we attempted to identify the age-specific susceptibility to the Omicron variant compared to the Delta and the pre-Delta strains, using the epidemiologic data of those three waves along with vaccine coverage data in South Korea. The force of infection (*λ*_*i*_) experienced by age group *i* was used to estimate the age-specific susceptibility in this study [5]. Given that South Korea has the National Infectious Disease Surveillance System (NIDSS) which mandates the web-based reporting of every COVID-19 case and the National Immunization Registry, it could be a suitable country to elucidate this topic [6, 7]. Considering the concordance between the laboratory-confirmed COVID-19 incidence and the national seroprevalence of SARS-CoV-2-specific anti-nucleocapsid (anti-*N*) antibodies (Fig. 1B), the ascertainment ratio in South Korea may not be far from reality [8].

## Methods

We adapted a previously described model which estimated the age-varying susceptibility to the Delta variant [9] and updated the model with recent vaccine coverage data and waning of vaccine effectiveness against the Omicron infection.

### Data

Age-stratified daily COVID-19 incidence and vaccine uptake rates have been reported in public by the Ministry of Health and Welfare of South Korea through NIDSS and National Immunization Registry [7, 8, 10]. More refined vaccination data of doses and manufacturers were provided by Korea Disease Control and Prevention Agency (KDCA) and National Health Insurance Service (NHIS). Age-structured population data was obtained from the Statistics Korea [11].

### Model construction

Following our previous study, we built an age-structured compartmental model stratified into 5-year age bands [9]. Compartments in the model were stratified by infection states (i.e., susceptible [*S*], exposed [*E*], infectious and pre-symptomatic [*Ipresym*], infectious and symptomatic [*Isym*], infectious and asymptomatic [*Iasym*], or quarantined [*Q*]), age band, and the transition time to the next infection state (Additional file 1: eMethods). In South Korea, individuals diagnosed with COVID-19 are isolated immediately; thus, the confirmation date could be regarded as the date on which quarantine started.

The strength of this model is that we know the diagnostic delay distribution (symptom onset to *Q*), transmission onset distribution relative to the symptom onset (*I* given symptom onset), and latent period distribution (*E* to *I*), based on the robust contact tracing study in South Korea (Table 1) [12]. For those who had never developed any symptoms (*Iasym*), we assumed that their latent period distribution was the same as that of individuals who developed symptoms (*Ipresym* → *Isym*) and that their infectious period distribution was the same as the total infectiousness period distribution of symptomatic individuals as suggested [13]. With this backward inference method, the remaining unknown distribution was the transition time from *S* to *E*, which depends on the force of infection. To estimate the parameters in the force of infection, we used a Bayesian inference method with a carefully designed Markov chain Monte Carlo (MCMC) algorithm. In this MCMC algorithm, we inferred the exposure times conditional on that the force of infection for each age group *i* was known and then inferred the force of infection given that the exposure times were available. We repeated these two steps several times until the Markov chain converged.

**Table 1:** Model parameters

	Value	Ref
Incubation period	*Gamma* (*μ*=4.544, *k*=1/0.709)	[12]
Transmission onset relative to the symptom onset	−4 + G*amma* (*μ*=5.266, *k*=1/0.8709)	[12]
Latent period	Incubation period + transmission onset relative to symptom onset	[12]
Delays from symptom onset to diagnosis	Empirical distribution from the raw data	[12]
Infectious period for asymptomatic cases	*Gamma* (*μ*=4, *k*=4/5)	[13]
Proportion of asymptomatic cases	52%, 50%, 45%, and 12% among individuals aged 0–4, 5–11, 12–17, and ≥18 years, respectively; or 50% in all age groups during the Omicron wave for sensitivity analysis	[14]
Relative infectiousness of asymptomatic cases	50% (25–75%)	[13, 15, 16]
Age groups (years)	[0–5], [5–10], [10–15], [15–20], [20–25], [25–30], [30–35], [35–40], [40–45], [45–50], [50–55], [55–60], [60–65], [65–70], [70–75], [≥75]	
Vaccine effectiveness against the Delta variant infection	BNT162b2 one dose ≥21 days: 57% (95% CI 50–63%)	[17–20]
	BNT162b2 two doses >14 days: 80% (95% CI 77–83%)	
	ChAdOx1 one dose ≥ 21 days: 46% (95% CI 35–55%)	
	ChAdOx1 two doses >14 days: 67% (95% CI 62–71%)	
	mRNA-1273 one dose ≥21 days: 75% (95% CI 64–83%)	
	mRNA-1273 two doses >14 days: 85% (95% CI 84–89%)	
	Ad26.COV2.S >14 days: 69% (95% CI 67–71%)	
Vaccine effectiveness against the Omicron variant infection	BNT162b2 one dose ≥18 days: 42.8% (95% CI 40.3–45.1%)	[21]
	BNT162b2 one dose >42 days: 31.5% (95% CI 29.9–33.1%)	
	BNT162b2 two doses <14 days: same value with one dose	
	BNT162b2 two doses >21 days: 65.5% (95% CI 63.9–67.0%)	
	BNT162b2 two doses >49 days: 48.7% (95% CI 47.1–50.2%)	
	BNT162b2 two doses >84 days: 30.1% (95% CI 28.7–31.5%)	
	BNT162b2 two doses >119 days: 15.4% (95% CI 14.2–16.6%)	
	BNT162b2 two doses >154 days: 11.5% (95% CI 10.1–12.9%)	
	BNT162b2 two doses >189 days: 8.8% (95% CI 7.0–10.5%)	
	Interpolate linearly in between	
	ChAdOx1 one dose ≥18 days: 17.7% (95% CI 14.3–21.0%)	
	ChAdOx1 one dose >42 days: 16.7% (95% CI 12.3–20.0%)	
	ChAdOx1 two doses <14 days: same value with one dose	
	ChAdOx1 two doses >21 days: 48.9% (95% CI 39.2–57.1%)	
	ChAdOx1 two doses >49 days: 33.7% (95% CI 25.0–41.5%)	
	ChAdOx1 two doses >84 days: 28.6% (95% CI 20.9–35.6%)	
	ChAdOx1 two doses >119 days: 17.8% (95% CI 13.4–21.9%)	
	ChAdOx1 two doses >154 days: 4.0% (95% CI 1.9–6.1%)	
	ChAdOx1 two doses >189 days: 0%	
	Interpolate linearly in between	
	mRNA-1273 one dose ≥18 days: 47.9% (95% CI 43.1–52.3%)	
	mRNA-1273 one dose >42 days: 31.9% (95% CI 27.3–36.1%)	
	mRNA-1273 two doses <14 days: same value with one dose	
	mRNA-1273 two doses >21 days: 75.1% (95% CI 70.8–78.7%)	
	mRNA-1273 two doses >49 days: 52.8% (95% CI 48.2–57.1%)	
	mRNA-1273 two doses >84 days: 35.6% (95% CI 32.7–38.4%)	
	mRNA-1273 two doses >119 days: 25.3% (95% CI 23.2–27.4%)	
	mRNA-1273 two doses >154 days: 15.0% (95% CI 11.6–18.2%)	
	mRNA-1273 two doses >189 days: 14.9% (95% CI 3.9–24.7%)	
	Interpolate linearly in between	
	Ad26.COV2.S^a^	
	Booster dose^b^ >10 days: 67.3% (95% CI 65.9–68.6%)	
	Booster dose^b^ >21 days: 66.9% (95% CI 65.6–68.1%)	
	Booster dose^b^ >49 days: 55.0% (95% CI 54.2–55.8%)	
	Booster dose^b^ >84 days: 45.7% (95% CI 44.7–46.7%)	
	Interpolate linearly in between	

*CI* confidence interval

^a^We regarded the vaccine effectiveness against the Omicron infection of Ad26.COV2.S was the same as that of ChAdOx1 due to unavailable reference data

^b^Whatever vaccine was used before (BNT162b2, ChAdOx1, mRNA-1273, or Ad26.COV2.S), we regarded the vaccine effectiveness of the booster dose as the same. In South Korea, BNT162b2 and mRNA-1273 have been used for booster shots approximately in a 2:1 ratio; thus, the weighted average of vaccine effectiveness values was used

According to Vynnycky and White [22], the force of infection *λ*_*i*_ is written as follows:

$${\lambda}_i=\sum_j{\beta}_{ij}{I}_j$$

Here, *β*_*ij*_ is the rate at which susceptible individuals in the age group *i* and infectious individuals in the age group *j* come into effective contact per unit time, and *Ι*_*j*_ is the number of infectious individuals in the age group *j*. We further divide *β*_*ij*_ into:

$${\beta}_{ij}={q}_i\frac{\phi_{ij}}{n_i}$$

Here, *q*_*i*_ is the probability that a contact between a susceptible individual in age group *i* and an infectious person leads to infection, *ϕ*_*ij*_ is the number of contacts an individual in age group *j* makes with those in age group *i* per unit time, and *n*_*i*_ is the number of individuals in age group *i*. Since we know the contact matrix for South Korea and the age-stratified incidence of COVID-19 at discrete time *t*, we could infer the *λ*_*i*_ (accordingly *q*_*i*_) of age group *i* [23]. To capture the changes of contact patterns as a result of social distancing measures, we considered school closure policies and reduced contact rates both at work and other places using Google mobility data (Fig. 1A, Additional file 1: Table S1 to S2) [24, 25]. Detailed Bayesian inference methods are available in Additional file 1: eMethods. All analyses were conducted using the *Python* statistical software version 3.6.13.

### Study period

The age-specific susceptibility (*q*_*i*_) during the 5th wave (Omicron driven, from January 1 to January 31, 2022) were compared with those during the 4th (Delta driven, from June 27 to August 21, 2021) and 3rd (pre-Delta, from October 15 to December 22, 2020) waves in South Korea. Since we know the domestic composition of variants during the study period (Fig. 1C), we only take into account the Omicron infections during the 5th wave and the Delta infections during the 4th wave.

As vaccine uptake increased, individuals who were vaccinated have been excluded from the susceptible population in accordance with the vaccine effectiveness against the Delta and the Omicron variants. The waning of vaccine effectiveness was also considered [21]. In detail, age-specific vaccine coverage data by vaccine doses and manufacturers have been reported weekly by the Ministry of Health and Welfare of South Korea (Additional file 1: Table S3) [7]. We divided the weekly number of immunized individuals by 7 to get a daily number of immunized individuals for the corresponding week and removed them from the susceptible population 2 weeks after the vaccination, considering the time to achieve immunity against COVID-19.

### Sensitivity analysis

There are uncertainties about these model parameters, including the age-specific contact patterns, the proportion of individuals who were infected and asymptomatic, and vaccine effectiveness. Therefore, we varied those values with sensitivity analyses. First, the number of contacts made in school was varied from 0.8-fold to 1.2-fold to the baseline, given that school-aged children have higher contact rates compared with other age groups and were likely to affect the result most. Second, considering the high asymptomatic infections with the Omicron variant, we increased the proportion of asymptomatic infections to 50% in all age groups [26]. At baseline, we adopted the prospective household cohort study reporting age-varying asymptomatic proportions (i.e., 52%, 50%, 45%, and 12% among individuals aged 0–4 years, 5–11 years, 12–17 years, and ≥18 years, respectively) [14]. For vaccine effectiveness, we adopted lower and upper bounds of the 95% confidence interval (CI) for sensitivity analyses as reported in another study [21].

## Results

**Fig. 2:**
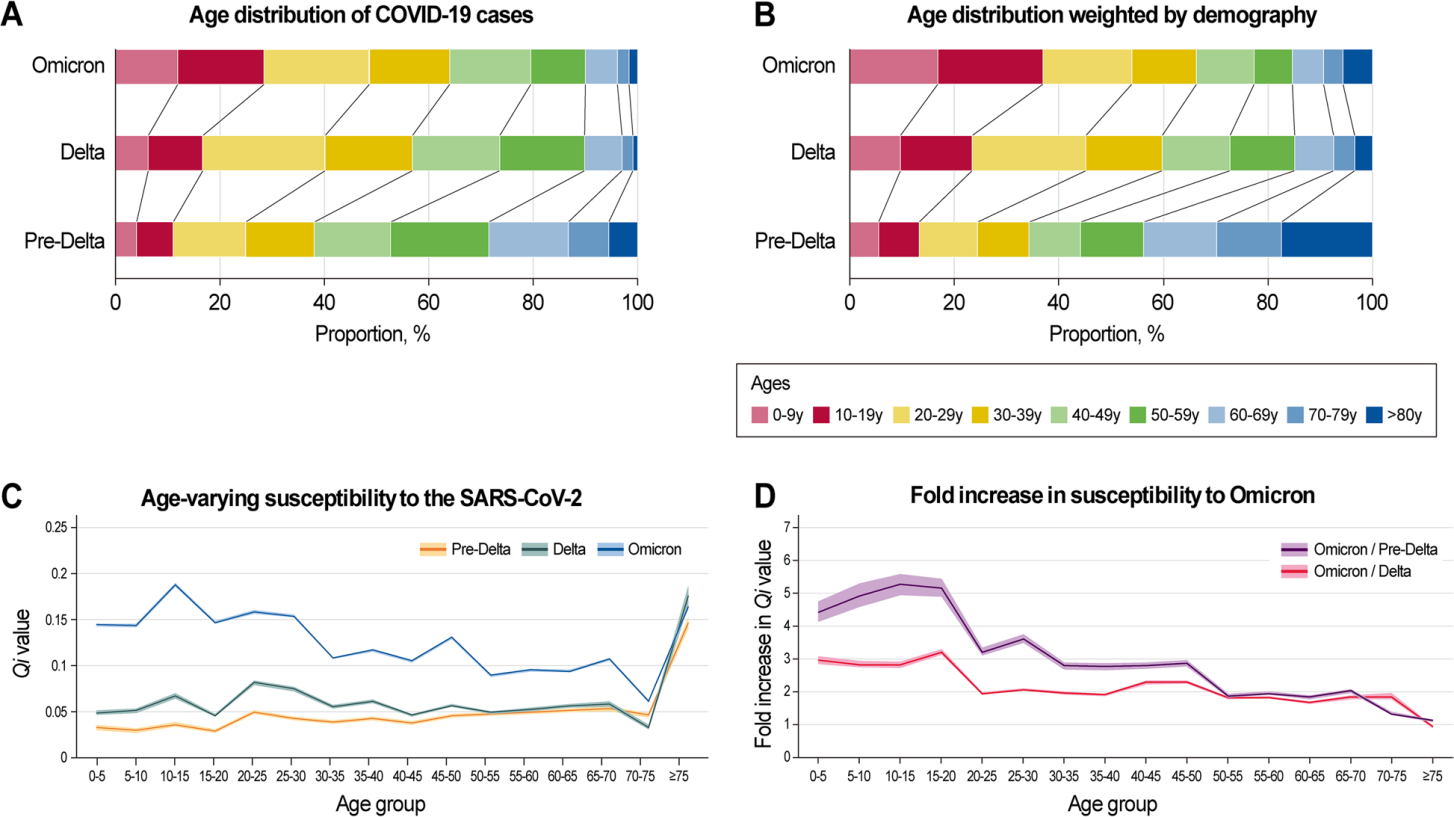
Age distribution of COVID-19 cases (**A**) during the 3rd, 4th, and 5th waves in South Korea and **B** those normalized by the demographic structure. The age-varying susceptibility to SARS-CoV-2 (**C**) during the 3rd (pre-Delta), 4th (Delta), and 5th (Omicron) waves in South Korea and **D** the fold rise in susceptibility to the Omicron/Delta and Omicron/pre-Delta by age groups. The shadow indicates the 95% confidence intervals

The age distribution of COVID-19 cases during the 3rd (pre-Delta), 4th (Delta), and 5th (Omicron) waves in South Korea is shown in Fig. 2A, B. The proportions of COVID-19 cases among those aged 19 years or less were 11.02%, 16.72%, and 28.55% during the 3rd, 4th, and 5th waves, respectively. Meanwhile, the proportions of cases among those aged 60 years or more were 28.47%, 10.16%, and 10.04% during the 3rd, 4th, and 5th waves, respectively. Considering the age demographics (age skewed older in South Korea), the normalized proportions of cases by age structure among those aged 19 years or less were 13.28%, 23.43%, and 36.95% during the 3rd, 4th, and 5th waves, respectively.

The estimated age-specific susceptibility to COVID-19 is shown in Fig. 2C, D. Both the 3rd (pre-Delta) and 4th (Delta) waves showed a similar age-dependent increase, whereas the 5th (Omicron) wave showed an inverted bell curve with bimodal peaks. A significant difference between the susceptibility to the Omicron and that to the Delta and pre-Delta variants was found in the younger age group. The rise in susceptibility to the Omicron/pre-Delta variant was highest in the 10–15 years age group (5.28 times [95% CI, 4.94–5.60]), whereas in those aged 50 years or more, the susceptibility to the Omicron/pre-Delta remained stable at approximately twofold, scoring the lowest value of 1.12 (95% CI, 1.09–1.14) among those aged 75 years or more. The rise in susceptibility to the Omicron/Delta variant was highest in the 15–19 years age group (3.21 times [95% CI, 3.12–3.31]) and lowest in those aged 75 years or more (0.93 times [95% CI, 0.89–0.97]).

For validation, we simulated the model forward with the estimated susceptibility parameters during the training period and verified the estimated case numbers could reproduce the observed epidemiological patterns equally well. We also presented the convergence of the Markov chain Monte Carlo (MCMC) algorithm through the trace plots and autocorrelation function (ACF) of the parameters. Varying the contact patterns, proportion of asymptomatic cases, and vaccine effectiveness in sensitivity analyses, the rise in susceptibility to the Omicron/pre-Omicron was not changed, recording the higher values in the 0–19 years age groups than in other age groups (Additional file 1: Fig. S1 to S12).

## Discussion

Even after adjusting for contact pattern, vaccination status, and waning of vaccine effectiveness, the age-specific susceptibility among age group 0–19 years was approximately 5 times higher during the 5th wave (Omicron driven) than that during the 3rd wave (pre-Delta) and 3 times higher than that during the 4th wave (Delta driven). Indeed, children are more susceptible to the Omicron variant compared with the previous strains of SARS-CoV-2. According to the US Centers for Disease Control and Prevention report, the hospitalization rates among individuals aged 12–17 years were 3.5 times as high during the peak week of the Omicron period than during the Delta period [27]. Previously mentioned pediatric admissions with COVID-19 infections in England also showed a 3-fold rise in 2 weeks from December 26, 2021 [3]. These findings are in line with our result, although the increased susceptibility to the infection is not necessarily correlated with the increased hospitalization rates.

Considering that the Omicron variant has shifted tropism to the upper respiratory tract from the lower respiratory tract, children whose upper airway is immature and relatively smaller than those in adults could be much more easily affected [28, 29]. Likewise, increasing cases of croup, an acute laryngotracheobronchitis characterized by barking cough, were noted in South Korea, during the Omicron surge [30]. What is more, the endocytic entry which Omicron prefers over the angiotensin-converting enzyme 2 (ACE2)-dependent pathway could also explain the higher number of pediatric cases since children have a lower number of ACE receptors [31, 32]. Taken together, our finding is in accordance with both epidemiological and biological observations.

There are several limitations in this study. First, the contact matrix in this study was not of our own empirical data, but instead mathematically estimated data [23]. Second, although we attempted to reflect the social distancing policy into our model, the implementation of nonpharmaceutical interventions (NPI), which could change the effective contact rates, was not fully considered in this study. However, the essential measures such as mask mandate, contact tracing, and mandatory quarantine for international arrival have been applied consistently throughout the study period (Additional file 1: Table S1). Moreover, the relative increase in susceptibility within the same age group would not change much, given the NPI use was likely to be consistent among the same age group [33]. Third, the exact proportion of asymptomatic infections by age groups remains unclear. To overcome this hurdle, we adopted the result from a prospective cohort study and further conducted a sensitivity analysis with variable ranges of asymptomatic proportions [14]. In addition, public health authorities in South Korea had conducted a mass screening test for those who had contact with confirmed COVID-19 cases until February 2022. For example, if there was one case in a school, whole classmates (occasionally whole students in the school) and their parents were screened for SARS-CoV-2 with either polymerase chain reaction (PCR) or rapid antigen test. Thus, we think it is unlikely to miss a significant number of cases in South Korea.

## Conclusions

In conclusion, large-scale testing, prompt epidemiological survey, and vaccination status records in a national registry in South Korea allowed us to analyze the age-stratified susceptibility to SARS-CoV-2. Generally, the Omicron variant of SARS-CoV-2 was estimated to spread more easily among children than the Delta and pre-Delta strains. At the beginning of the SARS-CoV-2 pandemic, an increased number of cases and a greater risk of severe disease with increasing age were notable. We might now see the course of adaptation of novel SARS-CoV-2 to humans. This age affinity seems to be similar with influenza that we include both the youngest and the elderly as target groups for vaccination. Although it is not yet clear whether children could be key driver groups in SARS-CoV-2 transmission hereafter, additional efforts for vaccinating children might be considered to reduce the pandemic’s impact on the whole community.

## Supplementary Information

**Additional file 1: eMethods.** Bayesian inference method to estimate the age-varying susceptibility to the SARS-CoV-2. **Figure S1.** Result of sensitivity analysis – Baseline. **Figure S2.** Result of sensitivity analysis – The proportion of asymptomatic cases = 50%. **Figure S3.** Result of sensitivity analysis – Varying contact rates at schools = 0.8 times from the baseline. **Figure S4.** Result of sensitivity analysis – Varying contact rates at schools = 1.2 times from the baseline. **Figure S5.** Result of sensitivity analysis – Vaccine efficacy = lower bound of 95% confidence interval. **Figure S6.** Result of sensitivity analysis – Vaccine efficacy = upper bound of 95% confidence interval. **Figure S7.** Model validation in 3^rd^ wave (pre-Delta). **Figure S8.** Model validation in 4^th^ wave (Delta). **Figure S9.** Model validation in 5^th^ wave (Omicron). **Figure S10.** MCMC trace plots and autocorrelation function (ACF) plots in 3^rd^ wave (pre-Delta). **Figure S11.** MCMC trace plots and autocorrelation function (ACF) plots in 4^th^ wave (Delta). **Figure S12.** MCMC trace plots and autocorrelation function (ACF) plots in 5^th^ wave (Omicron). **Table S1.** Overview of Social Distancing System in South Korea. **Table S2.** School Attendance Ratio (%) during the 4^th^ and 5^th^ waves. **Table S3.** Vaccine coverage data in South Korea.

## Data Availability

Code and data to reproduce the analyses are available at https://github.com/Hwichang/Age-varying-susceptibility-to-the-Omicron-variant-of-SARS-CoV-2.

## Abbreviations

ACE2: Angiotensin-converting enzyme 2
ACF: Autocorrelation function
anti-*N*: Anti-nucleocapsid
CI: Confidence interval
COVID-19: Coronavirus disease 2019
*E*: Exposed
*Iasym*: Infectious and asymptomatic
*Ipresym*: Infectious and pre-symptomatic
*Isym*: Infectious and symptomatic
MCMC: Markov chain Monte Carlo
NIDSS: National Infectious Disease Surveillance System
NPI: Nonpharmaceutical interventions
*Q*: Quarantined
*S*: Susceptible
US: United States
WHO: World Health Organization
