# Supplementary material for: Identifying susceptibility of children and adolescents to the Omicron variant (B.1.1.529)
Source: BMC Med. 2022 Nov 23;20:451. doi: 10.1186/s12916-022-02655-z (PMC9684890; doi:10.1186/s12916-022-02655-z)
Supplement: Supplementary file 1 — Additional file 1: eMethods. Bayesian inference method to estimate the age-varying susceptibility to the SARS-CoV-2. Figure S1. Result of sensitivity analysis – Baseline. Figure S2. Result of sensitivity analysis – The proportion of asymptomatic cases = 50%. Figure S3. Result of sensitivity analysis – Varying contact rates at schools = 0.8 times from the baseline. Figure S4. Result of sensitivity analysis – Varying contact rates at schools = 1.2 times from the baseline. Figure S5. Result of sensitivity analysis – Vaccine efficacy = lower bound of 95% confidence interval. Figure S6. Result of sensitivity analysis – Vaccine efficacy = upper bound of 95% confidence interval. Figure S7. Model validation in 3rd wave (pre-Delta). Figure S8. Model validation in 4th wave (Delta). Figure S9. Model validation in 5th wave (Omicron). Figure S10. MCMC trace plots and autocorrelation function (ACF) plots in 3rd wave (pre-Delta). Figure S11. MCMC trace plots and autocorrelation function (ACF) plots in 4th wave (Delta). Figure S12. MCMC trace plots and autocorrelation function (ACF) plots in 5th wave (Omicron). Table S1. Overview of Social Distancing System in South Korea. Table S2. School Attendance Ratio (%) during the 4th and 5th waves. Table S3. Vaccine coverage data in South Korea. [file 12916_2022_2655_MOESM1_ESM.docx]

**Additional file 1**

**eMethods.** Bayesian inference method to estimate the age-varying susceptibility to the SARS-CoV-2

**Fig. S1.** Result of sensitivity analysis – Baseline

**Fig. S2.** Result of sensitivity analysis – The proportion of asymptomatic cases = 50%

**Fig. S3.** Result of sensitivity analysis – Varying contact rates at schools = 0.8 times from the baseline

**Fig. S4.** Result of sensitivity analysis – Varying contact rates at schools = 1.2 times from the baseline

**Fig. S5.** Result of sensitivity analysis – Vaccine efficacy = lower bound of 95% confidence interval

**Fig. S6.** Result of sensitivity analysis – Vaccine efficacy = upper bound of 95% confidence interval

**Fig. S7.** Model validation in 3^rd^ wave (pre-Delta)

**Fig. S8.** Model validation in 4^th^ wave (Delta)

**Fig. S9.** Model validation in 5^th^ wave (Omicron)

**Fig. S10.** MCMC trace plots and autocorrelation function (ACF) plots in 3^rd^ wave (pre-Delta)

**Fig. S11.** MCMC trace plots and autocorrelation function (ACF) plots in 4^th^ wave (Delta)

**Fig. S12.** MCMC trace plots and autocorrelation function (ACF) plots in 5^th^ wave (Omicron)

**Table S1.** Overview of Social Distancing System in South Korea

**Table S2.** School Attendance Ratio (%) during the 4^th^ and 5^th^ waves

**Table S3.** Vaccine coverage data in South Korea

**eMethods**

We adopted a method in our previous study regarding the age-varying susceptibility to the Delta variant (doi:10.1001/jamanetworkopen.2022.3064 (2022)).

*Overview*

We built an age-structured compartment model stratified into 5-year age bands. We assumed that people are initially susceptible (*S*) and become exposed (*E*) after an effective contact with an infectious person. After a latent period, exposed individuals become infectious, either with a pre-symptomatic state ($I_{presym}$) followed by symptomatic infection ($I_{sym}$), or with an asymptomatic state ($I_{asym}$). After the infectious period, individuals enter the removed state due to isolation (*Q*).


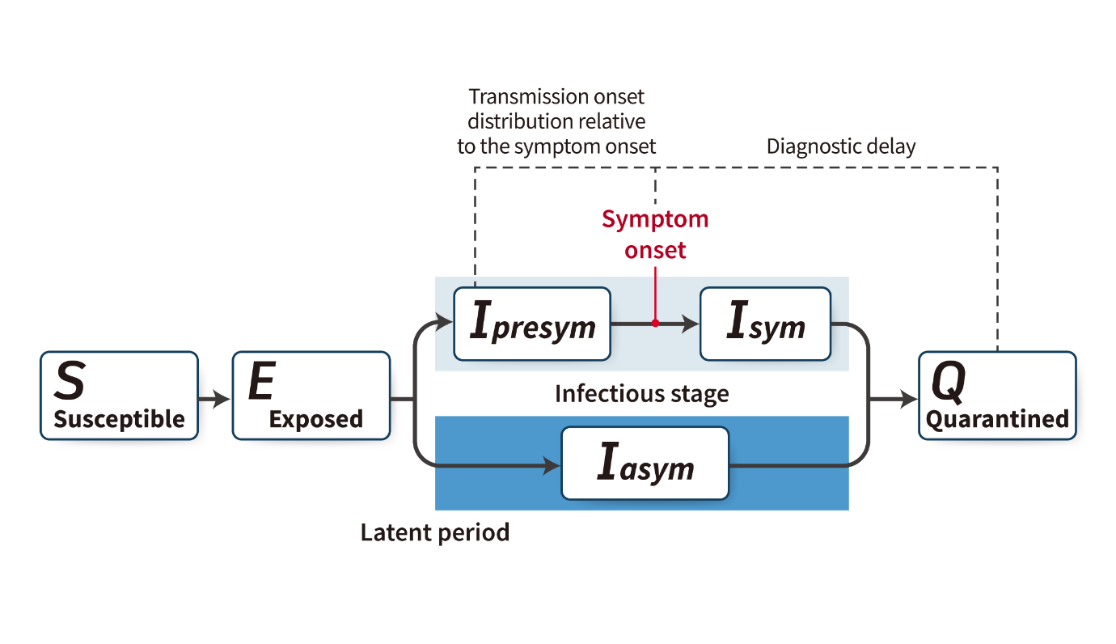


**Figure. A schematic plot of different time periods of the transmission of SARS-CoV-2.**

In a compartmental model, the force of infection $\lambda$, the rate at which susceptible individuals are infected (i.e exposed), is a crucial factor. The age-specific force of infection $\lambda_{i}$ in age group $i$ at discrete time $t$ could be written as:

$$\lambda_{i} = \mu\left( t | q_{i} \right)= \frac{q_{i}\sum_{j=1}^{A} \phi_{ij}\left( t \right)\left( I_{presym}^{j}\left( t-1 \right)+ I_{sym}^{j}\left( t-1 \right)+ 0.5I_{asym}^{j}\left( t-1 \right) \right)}{n_{i}} (S1)$$

where $q_{i}$ is the probability that a contact between a susceptible in age group $i$ and infectious person leads to infection, $\phi(t) = \phi_{ij}(t)$ means contact matrix at discrete time $t$ ($\phi_{ij}(t)$ is the number of contacts an individual of age group $j$ makes with those of age group $i$ per unit time at discrete time $t$), $n_{i}$ is the number of individuals in age group $i$. We suppose that the relative infectiousness of the $I_{asym}$is half of $I_{presym}$or $I_{sym}$. Here $i$ is in age group $\underline{A}$ $= \{1,2,\cdots,A\}$.

We try to estimate $q_{i}$ during the 3^rd^, 4^th^, and 5^th^ waves in South Korea. If we could observe the number of exposed individuals at time $t$ for age group $i$ and the number of total infectious individuals, the likelihood of the parameters could be easily derived. However, what we could observe is the number of diagnosed (i.e quarantined) individuals for age group $i \in\underline{A}$ at time $t,$only. In addition, asymptomatic infection which is the notable feature of COVID-19 should also be reflected in the model. To resolve these difficulties, we use a Bayesian approach. In particular, we develop an efficient MCMC (Markov Chain Monte Carlo) algorithm in which the exposed date, symptom onset date and transmission onset date for all quarantined individuals are imputed with an assumption that there are pre-determined proportion of asymptomatic individuals. We explain the details of our Bayesian method in the following three subsections.

*Data, Model and Posterior*

The data we used in the analysis is daily numbers of quarantined individuals for each age group from 15 October to 22 December 2020 (3^rd^ wave), from 27 June to 21 August 2021 (4^th^ wave), and from January 1 to January 31, 2022 (5^th^ wave).

To estimate $q_{i}$, we are going to impute the exposed dates, symptom onset dates and transmission onset dates of all quarantined individuals conditional on given quarantined dates. For this purpose, we need a probability model which relates the exposed dates, symptom onset dates and transmission onset dates to the quarantined dates.

| Symptomatic cases | | Asymptomatic cases | |
| --- | --- | --- | --- |
| Symbol | **Definition** | **Symbol** | **Definition** |
| *E* | Exposed date of an individual | ***E*** | Exposed date of an individual |
| *Y* | Incubation period | ***L*** | Latent period |
| *I* | Transmission onset time relative to the symptom onset | ***C*** | Infectious period suspended by quarantine |
| *D* | Diagnostic delay (i.e Quarantined) from the symptom onset | ***R*** | Infectious period |

For each symptomatic individual, the quarantined date is sum of the exposed date ($E$), the incubation period ($Y$) and the period for symptom onset to diagnostic delay ($D$). In addition, these individuals start infecting other susceptibles from the transmission onset date ($E+Y+I$).

For each asymptomatic individual, the quarantined date is sum of exposed date ($E$), latent period ($L$) and period for transmission onset to quarantined date ($C$). We assume that the latent period distribution of asymptomatic individuals is same as that of symptomatic individuals. Also, the quarantined time distribution of $C(f_{C})$ of asymptomatic individuals is set to be the exponential distribution with mean 1/1.7, which satisfies $P(C>R)=0.01$ where $E+L+R$ is defined as the recovered date.

Finally, the quarantined date $T$ is defined as

$$T = \left( E+Y+D \right)\boldsymbol{I}\left( \Delta=0 \right)+ \left( E+L+C \right)\boldsymbol{I}\left( \Delta=1 \right) (S2)$$

where $\Delta$ is equal to 0 when the individual is symptomatic and 1 when asymptomatic. We set $P(\Delta=1)=a_{i}$, which denotes the proportion of asymptomatic cases in age group $i$.

For individual $k$, let $W_{k} = (E_{k},Y_{k},I_{k},L_{k},C_{k},D_{k})$ and $V_{k} = (Y_{k},I_{k},L_{k},C_{k},D_{k})$. Let $D$ be the observed data which consist of the daily numbers of quarantined individuals. Our strategy to estimate $q_{i}$ is to generate $W_{k}$ and $q$ iteratively from their conditional posterior distributions $P(q_{i}|W,\underline{D})$ and $P(W_{k}|W_{(-k)},q,\underline{D})$ respectively, where $W =\{W_{k}\}$ and $W_{(-k)}$ denotes $W$ except $W_{k}$.

We could describe as,

$$P\left( W_{k} | W_{\left( -k \right)},q_{i_{k}},\underline{D} \right)= P\left( W_{k} | W_{\left( -k \right)},q_{i_{k}},D \right)\boldsymbol{I}\left( \Delta_{k}=0 \right)+ P\left( W_{k} | W_{\left( -k \right)},q_{i_{k}},D \right)\boldsymbol{I}\left( \Delta_{k}=1 \right) (S3)$$

$$= P(E_{k}|V_{\left( 1:N \right),}q_{i_{k}},D)(f_{Y}(Y_{k})f_{I}(I_{k})f_{D}(D_{k})\boldsymbol{I}(\Delta_{k}=0) +f_{L}(L_{k})f_{C}(C_{k})\boldsymbol{I}(\Delta_{k}=1)) (S4)$$

*Generating* $q$ *and* $W$ *from their conditional posterior distributions*

*Generating* $q$

For the prior distribution of $q_{i}$ we use a diffuse gamma distribution $Gamma(0.001,0.001)$ for all $i$. Then

$$p(q_{i}|W_{\left( 1:n_{i} \right)}^{i},\underline{D}) \propto p(q_{i})p(W_{\left( 1:n_{i} \right)}^{i},\underline{D}|q_{i}), i\in\underline{A} (S5)$$

when $W^{i} = \{W_{k} | The age group of k is i\}$, $n_{i}$ is the population for age group $i$.

In turn, $P(W_{1}^{i},\cdots,W_{n_{i}}^{i}, \underline{D}|q_{i})$ can be expressed as

$$P\left( W_{1}^{i},\cdots,W_{n_{i}}^{i},\underline{D} | q_{i} \right)=P\left( E_{1}^{i},\cdots,E_{n_{i}}^{i} | {V_{\left( 1:N \right)},q}_{i} \right)P\left( V_{\left( 1:N \right)} \right)= \prod_{k=1}^{n_{i}} P(E_{k}^{i}\left| {V_{\left( 1:N \right)},q}_{i} \right)P\left( V_{\left( 1:N \right)} \right) (S6)$$

$P(E_{k}^{i}| {V_{(1:N)},q}_{i})$ = $P(E_{k}^{i}| {I_{total}^{j}\left( t \right), t<E_{k}^{i} , q}_{i}) = p(E_{k}^{i} | q_{i})\prod_{t=1}^{E_{k}^{i}-1} (1-p(t|{\theta q}_{i})) (S7)$

Since $p(E_{k}^{i}|V_{(1:N)},q_{i})$ is the probability of an individual $k$ to be infected at discrete time $E_{k}^{i}$ (implying the individual $k$ has not been infected before).

$$P\left( V_{\left( 1:N \right)} \right)= \prod_{\Delta_{k}=0} (1-a_{i_{k}})f_{Y}\left( Y_{k} \right)f_{I}\left( I_{k} \right)f_{D}\left( D_{k} \right)\prod_{\Delta_{k}=1} a_{i_{k}}f_{L}\left( L_{k} \right)f_{C}\left( C_{k} \right) (S8)$$

where $N =\sum_{i} n_{i}$ (Total population size), $V_{k} = (Y_{k},I_{k},L_{k},C_{k},D_{k})$ and $i_{k}$ is the age group of individual $k$. By applying the following approximation,

$$P(E_{k}^{i}|V_{\left( 1:N \right)},q_{i_{k}}) = P(E_{k}^{i}| I_{total}^{j}(t) , t<E_{k}^{i} , q_{i_{k}}) = p(E_{k}^{i} | q_{i_{k}})\prod_{t=1}^{E_{k}^{i}-1} (1-p(t|q_{i_{k}}))\approx p(E_{k}^{i} \left| q_{i_{k}} \right)e^{-\sum_{t=1}^{E_{k}^{i}-1} p\left( t | q_{i_{k}} \right)} (S9)$$

we have

$$q_{i} | W, D \sim Gamma\left( 0.001 + \left| ℇ_{i}^{*} \right|, 0.001 + \sum_{k \inℇ_{i}^{*}} \frac{\sum_{t_{1}}^{E_{k}} \phi_{ij}\left( t \right)I_{total}^{j}\left( t-1 \right)}{n_{i}}+\sum_{k \in\left\{ s : E_{s}>t_{2} or E_{s}=NA \right\}} \frac{\sum_{t_{1}}^{t_{2}} \phi_{ij}\left( t \right)I_{total}^{j}\left( t-1 \right)}{n_{i}} \right) (S10)$$

where $ℇ_{i}^{*}$ is set of individuals in the age group $i$ exposed during the 3^rd^ wave $[t_{1},t_{2}]$ (or 4^th^ and 5^th^ waves).

*Generating* $W$

We generate $W$ by generating $W_{k}$ from $P(W_{k}|W_{(-k)},q,\underline{D})$ iteratively, and generate $W_{k}$ through the Metropolis-Hasting (MH) algorithm. To sample from the posterior $P(W_{k}|W_{(-k)},q,\underline{D})$ by the MH algorithm, we use the following proposal distribution:

$$Q\left( E_{k},V_{k} \right)= Q\left( E_{k} | V_{k} \right)Q\left( V_{k} \right) (S11)$$

$$Q\left( V_{k} \right)= p\left( V_{k} | \underline{D} \right) (S12)$$

$$Q\left( E_{k} | V_{k} \right)= \delta\left( T_{k}-Y_{k}-D_{k} \right)\boldsymbol{I}\left( \Delta_{k}=0 \right)+ \delta\left( T_{k}-L_{k}-C_{k} \right)\boldsymbol{I}\left( \Delta_{k}=1 \right) (S13)$$

Putting the above together, the sampling procedure of $W_{k}$ is summarized in Alogorithm S1.

**Alogorithm S1. Bayesian Inference**

**---------------------------------------------------------------------------------------------------------------------------------------**

Input : $W_{k}^{(0)}$ for $k = 1, 2, \cdots, N$

1. Sample $q^{(0)} = (q_{i}^{(0)})$ from prior.

2. **for** $m = 1:M$ (number of iteration) **do ► Gibbs sampling**

3. **for** $k = 1:N$ do **►** **MCMC**

4. Sample $W_{k}^{(m)}$ from $Q(E_{k},V_{k})$

5. $\alpha\leftarrow\frac{P(E_{k}^{(m)},E_{(-k)}^{(m-1)}| V_{k}^{(m)}, V_{(-k)}^{(m-1)}, q^{(m)} ,\underline{D})}{P(E_{k}^{(m-1)},E_{(-k)}^{(m-1)}| V_{k}^{(m-1)}, V_{(-k)}^{(m-1)}, q^{(m)} ,\underline{D})}$ **► Acceptance ratio**

6. $W_{k}^{(m)} \leftarrow\left\{ \begin{aligned} W_{k}^{(m)} \alpha\geq1 \\ W_{k}^{(m)} with prob \\ W_{k}^{(m-1)} else \end{aligned} \right.\alpha$

7. Sample $(q_{i}^{(m)})$ for $i = 1,2,\cdots,A$ from $p(q_{i}|W^{(m)})$

**---------------------------------------------------------------------------------------------------------------------------------------**

**Fig. S1.** Result of sensitivity analysis – Baseline


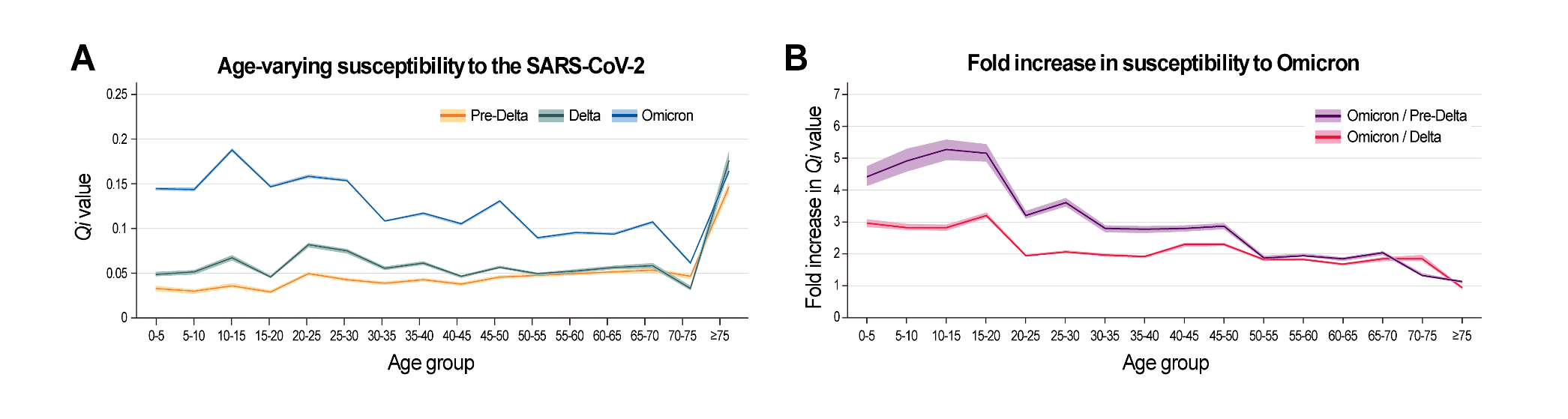


|  | Pre-Delta |  |  |  | Delta |  |  |  | Omicron |  |  |  | Omicron/Delta | |  |  | Omicron/Pre-Delta | |  |
| --- | --- | --- | --- | --- | --- | --- | --- | --- | --- | --- | --- | --- | --- | --- | --- | --- | --- | --- | --- |
|  | 2.50% | 50% | 97.50% |  | 2.50% | 50% | 97.50% |  | 2.50% | 50% | 97.50% |  | 2.50% | 50% | 97.50% |  | 2.50% | 50% | 97.50% |
| [0,5) | 0.030 | 0.033 | 0.035 |  | 0.046 | 0.049 | 0.051 |  | 0.143 | 0.144 | 0.146 |  | 3.077 | 2.964 | 2.854 |  | 4.762 | 4.420 | 4.131 |
| [5,10) | 0.027 | 0.029 | 0.032 |  | 0.048 | 0.051 | 0.053 |  | 0.142 | 0.144 | 0.145 |  | 2.946 | 2.831 | 2.741 |  | 5.294 | 4.923 | 4.578 |
| [10,15) | 0.033 | 0.036 | 0.038 |  | 0.064 | 0.067 | 0.070 |  | 0.186 | 0.188 | 0.190 |  | 2.920 | 2.813 | 2.719 |  | 5.599 | 5.278 | 4.938 |
| [15,20) | 0.027 | 0.029 | 0.030 |  | 0.044 | 0.046 | 0.048 |  | 0.146 | 0.147 | 0.149 |  | 3.310 | 3.213 | 3.120 |  | 5.448 | 5.157 | 4.895 |
| [20,25) | 0.047 | 0.049 | 0.052 |  | 0.079 | 0.081 | 0.084 |  | 0.157 | 0.158 | 0.160 |  | 1.990 | 1.950 | 1.915 |  | 3.344 | 3.214 | 3.100 |
| [25,30) | 0.041 | 0.043 | 0.044 |  | 0.072 | 0.074 | 0.077 |  | 0.152 | 0.154 | 0.155 |  | 2.107 | 2.068 | 2.027 |  | 3.742 | 3.611 | 3.489 |
| [30,35) | 0.037 | 0.039 | 0.041 |  | 0.053 | 0.055 | 0.057 |  | 0.107 | 0.108 | 0.109 |  | 2.007 | 1.967 | 1.923 |  | 2.904 | 2.788 | 2.687 |
| [35,40) | 0.040 | 0.042 | 0.044 |  | 0.059 | 0.061 | 0.063 |  | 0.116 | 0.117 | 0.118 |  | 1.950 | 1.908 | 1.873 |  | 2.865 | 2.763 | 2.665 |
| [40,45) | 0.036 | 0.038 | 0.039 |  | 0.044 | 0.046 | 0.047 |  | 0.104 | 0.105 | 0.106 |  | 2.343 | 2.285 | 2.236 |  | 2.899 | 2.789 | 2.698 |
| [45,50) | 0.044 | 0.046 | 0.048 |  | 0.055 | 0.057 | 0.058 |  | 0.129 | 0.130 | 0.132 |  | 2.354 | 2.304 | 2.259 |  | 2.955 | 2.862 | 2.766 |
| [50,55) | 0.046 | 0.047 | 0.049 |  | 0.047 | 0.049 | 0.051 |  | 0.088 | 0.089 | 0.090 |  | 1.855 | 1.817 | 1.781 |  | 1.931 | 1.878 | 1.829 |
| [55,60) | 0.047 | 0.049 | 0.051 |  | 0.050 | 0.052 | 0.054 |  | 0.094 | 0.095 | 0.096 |  | 1.860 | 1.826 | 1.787 |  | 2.000 | 1.948 | 1.903 |
| [60,65) | 0.049 | 0.051 | 0.053 |  | 0.054 | 0.056 | 0.059 |  | 0.092 | 0.094 | 0.095 |  | 1.709 | 1.672 | 1.625 |  | 1.880 | 1.833 | 1.780 |
| [65,70) | 0.050 | 0.053 | 0.055 |  | 0.055 | 0.058 | 0.061 |  | 0.105 | 0.107 | 0.109 |  | 1.897 | 1.841 | 1.783 |  | 2.092 | 2.030 | 1.962 |
| [70,75) | 0.043 | 0.046 | 0.049 |  | 0.030 | 0.033 | 0.035 |  | 0.060 | 0.061 | 0.063 |  | 1.962 | 1.855 | 1.774 |  | 1.379 | 1.330 | 1.291 |
| [75,+) | 0.141 | 0.147 | 0.153 |  | 0.166 | 0.176 | 0.187 |  | 0.161 | 0.164 | 0.166 |  | 0.972 | 0.933 | 0.892 |  | 1.144 | 1.118 | 1.087 |

**Fig. S2.** Result of sensitivity analysis – The proportion of asymptomatic cases = 50%


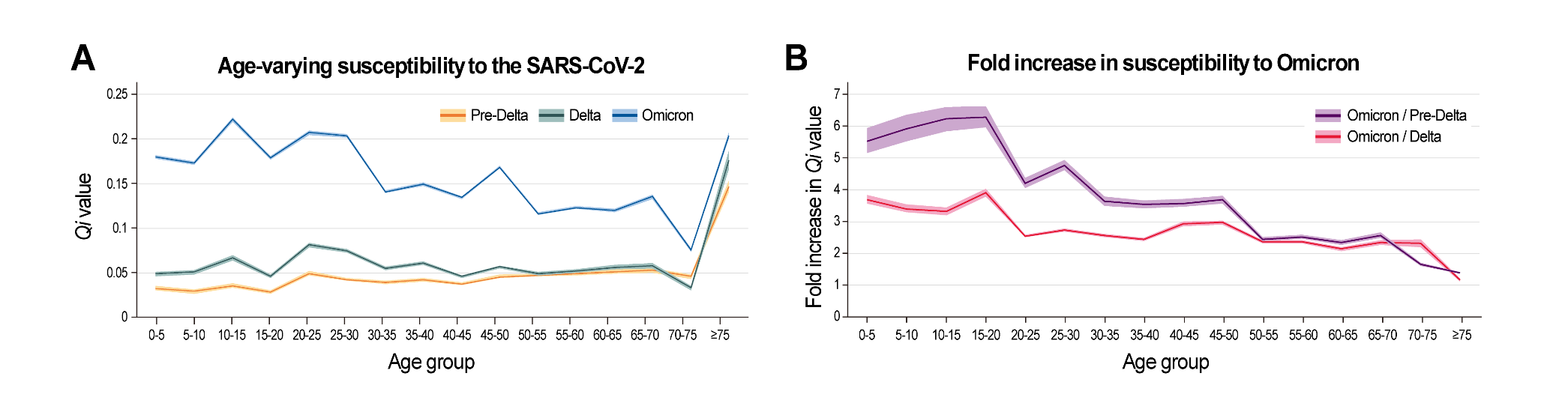


|  | Pre-Delta |  |  |  | Delta |  |  |  | Omicron |  |  |  | Omicron/Delta | |  |  | Omicron/Pre-Delta | |  |
| --- | --- | --- | --- | --- | --- | --- | --- | --- | --- | --- | --- | --- | --- | --- | --- | --- | --- | --- | --- |
|  | 2.50% | 50% | 97.50% |  | 2.50% | 50% | 97.50% |  | 2.50% | 50% | 97.50% |  | 2.50% | 50% | 97.50% |  | 2.50% | 50% | 97.50% |
| [0,5) | 0.030 | 0.033 | 0.035 |  | 0.046 | 0.049 | 0.051 |  | 0.178 | 0.180 | 0.182 |  | 3.837 | 3.700 | 3.561 |  | 5.937 | 5.516 | 5.154 |
| [5,10) | 0.027 | 0.029 | 0.032 |  | 0.048 | 0.051 | 0.053 |  | 0.171 | 0.173 | 0.175 |  | 3.535 | 3.403 | 3.300 |  | 6.352 | 5.917 | 5.512 |
| [10,15) | 0.033 | 0.036 | 0.038 |  | 0.064 | 0.067 | 0.070 |  | 0.219 | 0.222 | 0.224 |  | 3.441 | 3.319 | 3.211 |  | 6.599 | 6.228 | 5.832 |
| [15,20) | 0.027 | 0.029 | 0.030 |  | 0.044 | 0.046 | 0.048 |  | 0.177 | 0.179 | 0.181 |  | 4.028 | 3.913 | 3.797 |  | 6.630 | 6.280 | 5.958 |
| [20,25) | 0.047 | 0.049 | 0.052 |  | 0.079 | 0.081 | 0.084 |  | 0.205 | 0.207 | 0.209 |  | 2.597 | 2.551 | 2.503 |  | 4.363 | 4.204 | 4.052 |
| [25,30) | 0.041 | 0.043 | 0.044 |  | 0.072 | 0.074 | 0.077 |  | 0.201 | 0.203 | 0.205 |  | 2.786 | 2.737 | 2.682 |  | 4.947 | 4.778 | 4.615 |
| [30,35) | 0.037 | 0.039 | 0.041 |  | 0.053 | 0.055 | 0.057 |  | 0.139 | 0.141 | 0.142 |  | 2.612 | 2.565 | 2.506 |  | 3.780 | 3.636 | 3.500 |
| [35,40) | 0.040 | 0.042 | 0.044 |  | 0.059 | 0.061 | 0.063 |  | 0.148 | 0.149 | 0.151 |  | 2.494 | 2.444 | 2.396 |  | 3.664 | 3.540 | 3.411 |
| [40,45) | 0.036 | 0.038 | 0.039 |  | 0.044 | 0.046 | 0.047 |  | 0.133 | 0.134 | 0.136 |  | 2.998 | 2.928 | 2.867 |  | 3.709 | 3.574 | 3.459 |
| [45,50) | 0.044 | 0.046 | 0.048 |  | 0.055 | 0.057 | 0.058 |  | 0.167 | 0.168 | 0.170 |  | 3.039 | 2.976 | 2.917 |  | 3.813 | 3.697 | 3.572 |
| [50,55) | 0.046 | 0.047 | 0.049 |  | 0.047 | 0.049 | 0.051 |  | 0.115 | 0.116 | 0.118 |  | 2.416 | 2.366 | 2.322 |  | 2.515 | 2.445 | 2.384 |
| [55,60) | 0.047 | 0.049 | 0.051 |  | 0.050 | 0.052 | 0.054 |  | 0.121 | 0.123 | 0.124 |  | 2.412 | 2.360 | 2.312 |  | 2.593 | 2.518 | 2.461 |
| [60,65) | 0.049 | 0.051 | 0.053 |  | 0.054 | 0.056 | 0.059 |  | 0.118 | 0.120 | 0.122 |  | 2.188 | 2.137 | 2.080 |  | 2.407 | 2.342 | 2.278 |
| [65,70) | 0.050 | 0.053 | 0.055 |  | 0.055 | 0.058 | 0.061 |  | 0.133 | 0.135 | 0.138 |  | 2.406 | 2.331 | 2.258 |  | 2.654 | 2.571 | 2.485 |
| [70,75) | 0.043 | 0.046 | 0.049 |  | 0.030 | 0.033 | 0.035 |  | 0.074 | 0.076 | 0.078 |  | 2.438 | 2.307 | 2.197 |  | 1.713 | 1.654 | 1.599 |
| [75,+) | 0.141 | 0.147 | 0.153 |  | 0.166 | 0.176 | 0.187 |  | 0.200 | 0.204 | 0.207 |  | 1.208 | 1.159 | 1.109 |  | 1.423 | 1.390 | 1.353 |

**Fig. S3.** Result of sensitivity analysis – Varying contact rates at schools = 0.8 times from the baseline


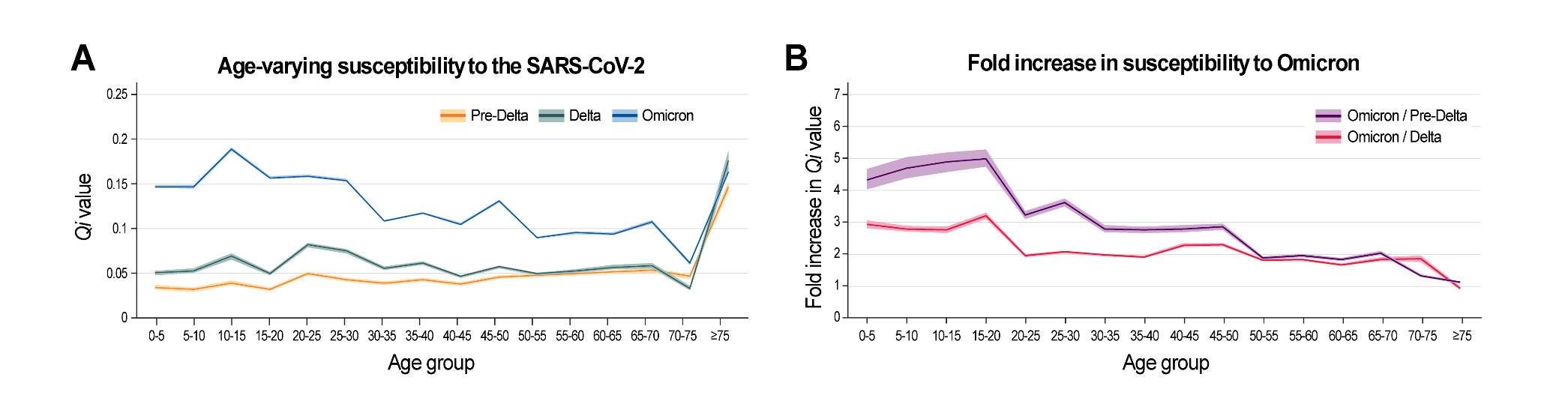


|  | Pre-Delta |  |  |  | Delta |  |  |  | Omicron |  |  |  | Omicron/Delta | |  |  | Omicron/Pre-Delta | |  |
| --- | --- | --- | --- | --- | --- | --- | --- | --- | --- | --- | --- | --- | --- | --- | --- | --- | --- | --- | --- |
|  | 2.50% | 50% | 97.50% |  | 2.50% | 50% | 97.50% |  | 2.50% | 50% | 97.50% |  | 2.50% | 50% | 97.50% |  | 2.50% | 50% | 97.50% |
| [0,5) | 0.031 | 0.034 | 0.037 |  | 0.048 | 0.050 | 0.053 |  | 0.145 | 0.147 | 0.149 |  | 3.049 | 2.938 | 2.823 |  | 4.674 | 4.334 | 4.045 |
| [5,10) | 0.029 | 0.031 | 0.034 |  | 0.050 | 0.053 | 0.055 |  | 0.145 | 0.146 | 0.148 |  | 2.896 | 2.789 | 2.705 |  | 5.041 | 4.696 | 4.375 |
| [10,15) | 0.036 | 0.039 | 0.042 |  | 0.065 | 0.068 | 0.072 |  | 0.187 | 0.189 | 0.191 |  | 2.859 | 2.759 | 2.664 |  | 5.183 | 4.891 | 4.583 |
| [15,20) | 0.029 | 0.031 | 0.033 |  | 0.047 | 0.049 | 0.051 |  | 0.155 | 0.157 | 0.158 |  | 3.300 | 3.205 | 3.107 |  | 5.290 | 5.002 | 4.740 |
| [20,25) | 0.047 | 0.049 | 0.052 |  | 0.079 | 0.081 | 0.084 |  | 0.157 | 0.159 | 0.160 |  | 1.995 | 1.953 | 1.915 |  | 3.350 | 3.219 | 3.103 |
| [25,30) | 0.041 | 0.043 | 0.044 |  | 0.072 | 0.074 | 0.077 |  | 0.152 | 0.154 | 0.155 |  | 2.108 | 2.070 | 2.028 |  | 3.743 | 3.616 | 3.491 |
| [30,35) | 0.037 | 0.039 | 0.041 |  | 0.053 | 0.055 | 0.057 |  | 0.107 | 0.108 | 0.109 |  | 2.010 | 1.971 | 1.925 |  | 2.908 | 2.795 | 2.689 |
| [35,40) | 0.040 | 0.042 | 0.044 |  | 0.059 | 0.061 | 0.063 |  | 0.116 | 0.117 | 0.118 |  | 1.951 | 1.911 | 1.871 |  | 2.867 | 2.768 | 2.665 |
| [40,45) | 0.036 | 0.038 | 0.039 |  | 0.044 | 0.046 | 0.047 |  | 0.104 | 0.105 | 0.106 |  | 2.344 | 2.284 | 2.231 |  | 2.901 | 2.789 | 2.693 |
| [45,50) | 0.044 | 0.046 | 0.048 |  | 0.055 | 0.057 | 0.058 |  | 0.129 | 0.130 | 0.132 |  | 2.351 | 2.303 | 2.260 |  | 2.951 | 2.862 | 2.768 |
| [50,55) | 0.046 | 0.047 | 0.049 |  | 0.047 | 0.049 | 0.051 |  | 0.088 | 0.089 | 0.090 |  | 1.856 | 1.816 | 1.782 |  | 1.932 | 1.877 | 1.831 |
| [55,60) | 0.047 | 0.049 | 0.051 |  | 0.050 | 0.052 | 0.054 |  | 0.094 | 0.095 | 0.096 |  | 1.862 | 1.827 | 1.788 |  | 2.002 | 1.949 | 1.904 |
| [60,65) | 0.049 | 0.051 | 0.053 |  | 0.054 | 0.056 | 0.059 |  | 0.093 | 0.094 | 0.095 |  | 1.714 | 1.672 | 1.627 |  | 1.885 | 1.832 | 1.783 |
| [65,70) | 0.050 | 0.053 | 0.055 |  | 0.055 | 0.058 | 0.061 |  | 0.105 | 0.107 | 0.109 |  | 1.907 | 1.842 | 1.789 |  | 2.102 | 2.032 | 1.970 |
| [70,75) | 0.043 | 0.046 | 0.049 |  | 0.030 | 0.033 | 0.035 |  | 0.060 | 0.061 | 0.063 |  | 1.959 | 1.852 | 1.772 |  | 1.376 | 1.328 | 1.290 |
| [75,+) | 0.141 | 0.147 | 0.153 |  | 0.166 | 0.176 | 0.187 |  | 0.162 | 0.164 | 0.166 |  | 0.975 | 0.932 | 0.891 |  | 1.148 | 1.117 | 1.087 |

**Fig. S4.** Result of sensitivity analysis – Varying contact rates at schools = 1.2 times from the baseline


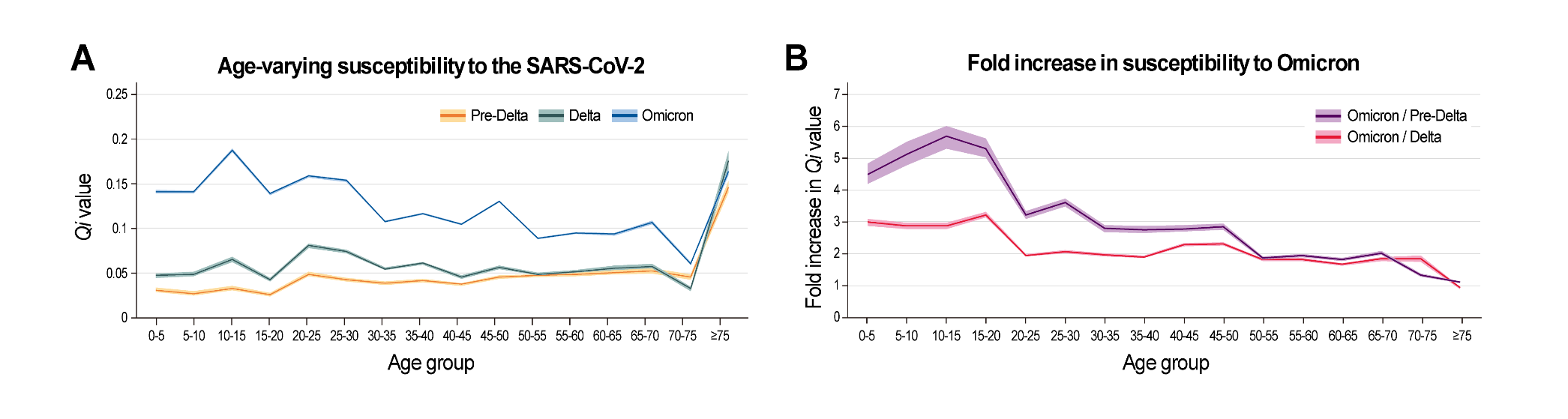


|  | Pre-Delta |  |  |  | Delta |  |  |  | Omicron |  |  |  | Omicron/Delta | |  |  | Omicron/Pre-Delta | |  |
| --- | --- | --- | --- | --- | --- | --- | --- | --- | --- | --- | --- | --- | --- | --- | --- | --- | --- | --- | --- |
|  | 2.50% | 50% | 97.50% |  | 2.50% | 50% | 97.50% |  | 2.50% | 50% | 97.50% |  | 2.50% | 50% | 97.50% |  | 2.50% | 50% | 97.50% |
| [0,5) | 0.029 | 0.032 | 0.034 |  | 0.045 | 0.047 | 0.050 |  | 0.140 | 0.142 | 0.143 |  | 3.097 | 2.988 | 2.880 |  | 4.837 | 4.494 | 4.206 |
| [5,10) | 0.025 | 0.027 | 0.030 |  | 0.047 | 0.049 | 0.051 |  | 0.139 | 0.141 | 0.143 |  | 2.985 | 2.871 | 2.780 |  | 5.522 | 5.139 | 4.788 |
| [10,15) | 0.031 | 0.033 | 0.036 |  | 0.062 | 0.065 | 0.068 |  | 0.185 | 0.187 | 0.189 |  | 2.980 | 2.872 | 2.773 |  | 6.015 | 5.679 | 5.309 |
| [15,20) | 0.025 | 0.026 | 0.028 |  | 0.041 | 0.043 | 0.045 |  | 0.137 | 0.139 | 0.140 |  | 3.327 | 3.229 | 3.136 |  | 5.610 | 5.302 | 5.022 |
| [20,25) | 0.047 | 0.049 | 0.052 |  | 0.079 | 0.081 | 0.084 |  | 0.157 | 0.159 | 0.160 |  | 1.991 | 1.953 | 1.915 |  | 3.343 | 3.216 | 3.100 |
| [25,30) | 0.041 | 0.043 | 0.045 |  | 0.072 | 0.074 | 0.077 |  | 0.152 | 0.154 | 0.155 |  | 2.109 | 2.069 | 2.028 |  | 3.743 | 3.611 | 3.487 |
| [30,35) | 0.037 | 0.039 | 0.041 |  | 0.053 | 0.055 | 0.057 |  | 0.107 | 0.108 | 0.109 |  | 2.009 | 1.970 | 1.922 |  | 2.906 | 2.793 | 2.685 |
| [35,40) | 0.040 | 0.042 | 0.044 |  | 0.059 | 0.061 | 0.063 |  | 0.116 | 0.117 | 0.118 |  | 1.952 | 1.909 | 1.870 |  | 2.868 | 2.763 | 2.661 |
| [40,45) | 0.036 | 0.038 | 0.039 |  | 0.044 | 0.046 | 0.047 |  | 0.104 | 0.105 | 0.106 |  | 2.341 | 2.284 | 2.235 |  | 2.895 | 2.787 | 2.697 |
| [45,50) | 0.044 | 0.046 | 0.048 |  | 0.055 | 0.057 | 0.058 |  | 0.129 | 0.130 | 0.132 |  | 2.354 | 2.303 | 2.260 |  | 2.953 | 2.860 | 2.765 |
| [50,55) | 0.046 | 0.047 | 0.049 |  | 0.047 | 0.049 | 0.051 |  | 0.088 | 0.089 | 0.090 |  | 1.857 | 1.820 | 1.782 |  | 1.933 | 1.880 | 1.829 |
| [55,60) | 0.047 | 0.049 | 0.051 |  | 0.050 | 0.052 | 0.054 |  | 0.094 | 0.095 | 0.096 |  | 1.863 | 1.824 | 1.783 |  | 2.002 | 1.945 | 1.899 |
| [60,65) | 0.049 | 0.051 | 0.053 |  | 0.054 | 0.056 | 0.059 |  | 0.092 | 0.094 | 0.095 |  | 1.708 | 1.670 | 1.622 |  | 1.879 | 1.830 | 1.777 |
| [65,70) | 0.050 | 0.053 | 0.055 |  | 0.055 | 0.058 | 0.061 |  | 0.105 | 0.107 | 0.109 |  | 1.899 | 1.841 | 1.784 |  | 2.094 | 2.029 | 1.964 |
| [70,75) | 0.043 | 0.046 | 0.049 |  | 0.030 | 0.033 | 0.035 |  | 0.060 | 0.061 | 0.062 |  | 1.959 | 1.853 | 1.763 |  | 1.376 | 1.328 | 1.283 |
| [75,+) | 0.141 | 0.147 | 0.153 |  | 0.166 | 0.176 | 0.187 |  | 0.160 | 0.164 | 0.167 |  | 0.968 | 0.931 | 0.893 |  | 1.140 | 1.116 | 1.089 |

**Fig. S5.** Result of sensitivity analysis – Vaccine efficacy = lower bound of 95% confidence interval


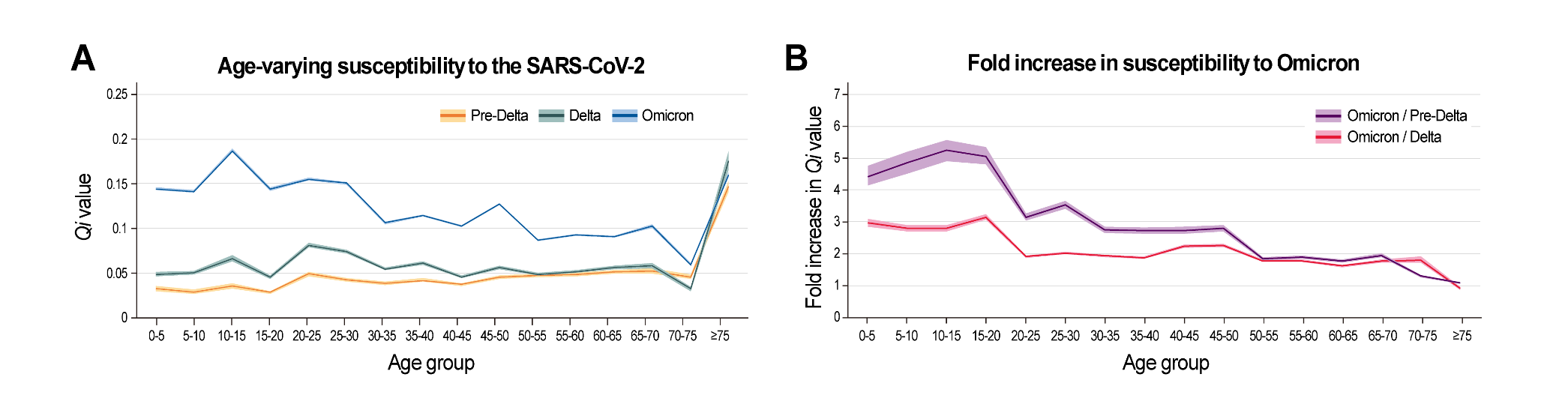


|  | Pre-Delta |  |  |  | Delta |  |  |  | Omicron |  |  |  | Omicron/Delta | |  |  | Omicron/Pre-Delta | |  |
| --- | --- | --- | --- | --- | --- | --- | --- | --- | --- | --- | --- | --- | --- | --- | --- | --- | --- | --- | --- |
|  | 2.50% | 50% | 97.50% |  | 2.50% | 50% | 97.50% |  | 2.50% | 50% | 97.50% |  | 2.50% | 50% | 97.50% |  | 2.50% | 50% | 97.50% |
| [0,5) | 0.030 | 0.033 | 0.035 |  | 0.046 | 0.049 | 0.051 |  | 0.143 | 0.144 | 0.146 |  | 3.080 | 2.966 | 2.856 |  | 4.766 | 4.423 | 4.133 |
| [5,10) | 0.027 | 0.029 | 0.032 |  | 0.048 | 0.051 | 0.053 |  | 0.140 | 0.141 | 0.143 |  | 2.896 | 2.788 | 2.702 |  | 5.204 | 4.847 | 4.513 |
| [10,15) | 0.033 | 0.036 | 0.038 |  | 0.064 | 0.067 | 0.070 |  | 0.185 | 0.187 | 0.189 |  | 2.907 | 2.797 | 2.705 |  | 5.575 | 5.248 | 4.912 |
| [15,20) | 0.027 | 0.029 | 0.030 |  | 0.044 | 0.046 | 0.048 |  | 0.143 | 0.144 | 0.146 |  | 3.241 | 3.151 | 3.057 |  | 5.335 | 5.058 | 4.797 |
| [20,25) | 0.047 | 0.049 | 0.052 |  | 0.079 | 0.081 | 0.084 |  | 0.154 | 0.155 | 0.157 |  | 1.949 | 1.910 | 1.874 |  | 3.274 | 3.148 | 3.034 |
| [25,30) | 0.041 | 0.043 | 0.044 |  | 0.072 | 0.074 | 0.077 |  | 0.149 | 0.151 | 0.152 |  | 2.065 | 2.027 | 1.986 |  | 3.666 | 3.539 | 3.417 |
| [30,35) | 0.037 | 0.039 | 0.041 |  | 0.053 | 0.055 | 0.057 |  | 0.105 | 0.106 | 0.107 |  | 1.972 | 1.934 | 1.890 |  | 2.854 | 2.741 | 2.641 |
| [35,40) | 0.040 | 0.042 | 0.044 |  | 0.059 | 0.061 | 0.063 |  | 0.114 | 0.115 | 0.116 |  | 1.919 | 1.876 | 1.841 |  | 2.820 | 2.717 | 2.621 |
| [40,45) | 0.036 | 0.038 | 0.039 |  | 0.044 | 0.046 | 0.047 |  | 0.102 | 0.102 | 0.104 |  | 2.295 | 2.233 | 2.185 |  | 2.839 | 2.726 | 2.637 |
| [45,50) | 0.044 | 0.046 | 0.048 |  | 0.055 | 0.057 | 0.058 |  | 0.126 | 0.128 | 0.129 |  | 2.300 | 2.254 | 2.209 |  | 2.886 | 2.800 | 2.705 |
| [50,55) | 0.046 | 0.047 | 0.049 |  | 0.047 | 0.049 | 0.051 |  | 0.086 | 0.087 | 0.088 |  | 1.814 | 1.778 | 1.742 |  | 1.888 | 1.837 | 1.789 |
| [55,60) | 0.047 | 0.049 | 0.051 |  | 0.050 | 0.052 | 0.054 |  | 0.091 | 0.093 | 0.094 |  | 1.817 | 1.780 | 1.741 |  | 1.954 | 1.899 | 1.854 |
| [60,65) | 0.049 | 0.051 | 0.053 |  | 0.054 | 0.056 | 0.059 |  | 0.090 | 0.091 | 0.092 |  | 1.657 | 1.615 | 1.568 |  | 1.823 | 1.770 | 1.717 |
| [65,70) | 0.050 | 0.053 | 0.055 |  | 0.055 | 0.058 | 0.061 |  | 0.101 | 0.103 | 0.105 |  | 1.828 | 1.768 | 1.716 |  | 2.017 | 1.950 | 1.888 |
| [70,75) | 0.043 | 0.046 | 0.049 |  | 0.030 | 0.033 | 0.035 |  | 0.058 | 0.059 | 0.061 |  | 1.910 | 1.805 | 1.727 |  | 1.342 | 1.294 | 1.257 |
| [75,+) | 0.141 | 0.147 | 0.153 |  | 0.166 | 0.176 | 0.187 |  | 0.158 | 0.160 | 0.163 |  | 0.951 | 0.912 | 0.873 |  | 1.119 | 1.093 | 1.065 |

**Fig. S6.** Result of sensitivity analysis – Vaccine efficacy = upper bound of 95% confidence interval


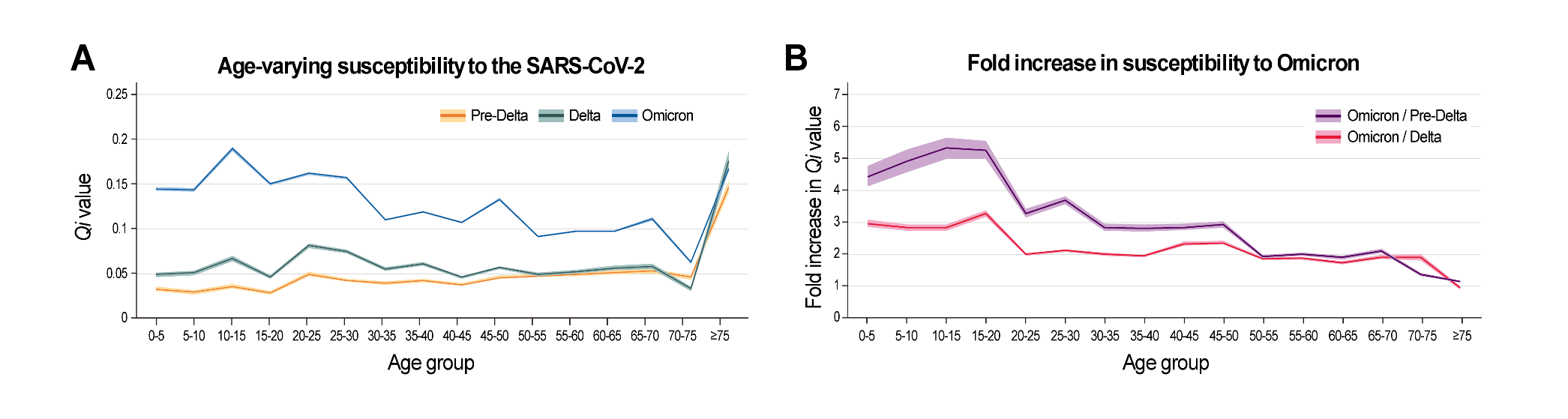


|  | Pre-Delta |  |  |  | Delta |  |  |  | Omicron |  |  |  | Omicron/Delta | |  |  | Omicron/Pre-Delta | |  |
| --- | --- | --- | --- | --- | --- | --- | --- | --- | --- | --- | --- | --- | --- | --- | --- | --- | --- | --- | --- |
|  | 2.50% | 50% | 97.50% |  | 2.50% | 50% | 97.50% |  | 2.50% | 50% | 97.50% |  | 2.50% | 50% | 97.50% |  | 2.50% | 50% | 97.50% |
| [0,5) | 0.030 | 0.033 | 0.035 |  | 0.046 | 0.049 | 0.051 |  | 0.143 | 0.144 | 0.146 |  | 3.078 | 2.964 | 2.852 |  | 4.763 | 4.419 | 4.128 |
| [5,10) | 0.027 | 0.029 | 0.032 |  | 0.048 | 0.051 | 0.053 |  | 0.142 | 0.144 | 0.145 |  | 2.939 | 2.828 | 2.735 |  | 5.282 | 4.918 | 4.568 |
| [10,15) | 0.033 | 0.036 | 0.038 |  | 0.064 | 0.067 | 0.070 |  | 0.187 | 0.189 | 0.191 |  | 2.940 | 2.834 | 2.743 |  | 5.639 | 5.317 | 4.982 |
| [15,20) | 0.027 | 0.029 | 0.030 |  | 0.044 | 0.046 | 0.048 |  | 0.148 | 0.150 | 0.151 |  | 3.374 | 3.277 | 3.175 |  | 5.553 | 5.259 | 4.981 |
| [20,25) | 0.047 | 0.049 | 0.052 |  | 0.079 | 0.081 | 0.084 |  | 0.160 | 0.162 | 0.163 |  | 2.033 | 1.992 | 1.953 |  | 3.416 | 3.283 | 3.161 |
| [25,30) | 0.041 | 0.043 | 0.044 |  | 0.072 | 0.074 | 0.077 |  | 0.156 | 0.157 | 0.159 |  | 2.153 | 2.113 | 2.072 |  | 3.822 | 3.689 | 3.566 |
| [30,35) | 0.037 | 0.039 | 0.041 |  | 0.053 | 0.055 | 0.057 |  | 0.109 | 0.110 | 0.111 |  | 2.045 | 2.004 | 1.957 |  | 2.960 | 2.841 | 2.734 |
| [35,40) | 0.040 | 0.042 | 0.044 |  | 0.059 | 0.061 | 0.063 |  | 0.118 | 0.119 | 0.120 |  | 1.986 | 1.946 | 1.906 |  | 2.918 | 2.819 | 2.712 |
| [40,45) | 0.036 | 0.038 | 0.039 |  | 0.044 | 0.046 | 0.047 |  | 0.106 | 0.107 | 0.108 |  | 2.392 | 2.331 | 2.280 |  | 2.960 | 2.845 | 2.751 |
| [45,50) | 0.044 | 0.046 | 0.048 |  | 0.055 | 0.057 | 0.058 |  | 0.132 | 0.133 | 0.135 |  | 2.408 | 2.352 | 2.307 |  | 3.022 | 2.921 | 2.825 |
| [50,55) | 0.046 | 0.047 | 0.049 |  | 0.047 | 0.049 | 0.051 |  | 0.090 | 0.091 | 0.092 |  | 1.902 | 1.861 | 1.824 |  | 1.980 | 1.923 | 1.873 |
| [55,60) | 0.047 | 0.049 | 0.051 |  | 0.050 | 0.052 | 0.054 |  | 0.096 | 0.097 | 0.098 |  | 1.908 | 1.867 | 1.826 |  | 2.051 | 1.991 | 1.944 |
| [60,65) | 0.049 | 0.051 | 0.053 |  | 0.054 | 0.056 | 0.059 |  | 0.096 | 0.097 | 0.098 |  | 1.774 | 1.728 | 1.680 |  | 1.951 | 1.894 | 1.840 |
| [65,70) | 0.050 | 0.053 | 0.055 |  | 0.055 | 0.058 | 0.061 |  | 0.109 | 0.111 | 0.113 |  | 1.973 | 1.911 | 1.853 |  | 2.176 | 2.108 | 2.040 |
| [70,75) | 0.043 | 0.046 | 0.049 |  | 0.030 | 0.033 | 0.035 |  | 0.061 | 0.062 | 0.064 |  | 2.004 | 1.898 | 1.810 |  | 1.408 | 1.361 | 1.317 |
| [75,+) | 0.141 | 0.147 | 0.153 |  | 0.166 | 0.176 | 0.187 |  | 0.164 | 0.167 | 0.169 |  | 0.991 | 0.951 | 0.908 |  | 1.167 | 1.140 | 1.107 |

**Fig. S7.** Model validation in 3^rd^ wave (pre-Delta)


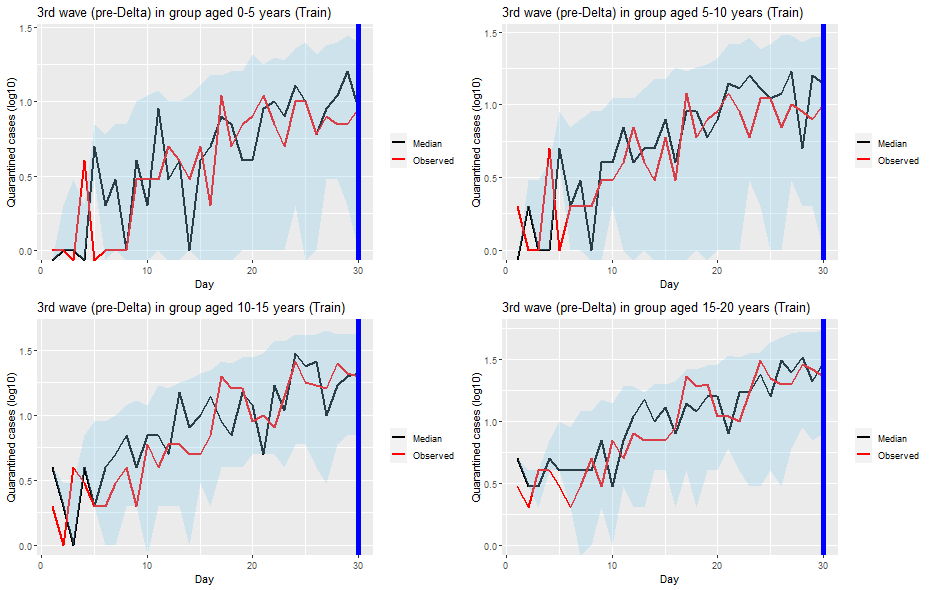


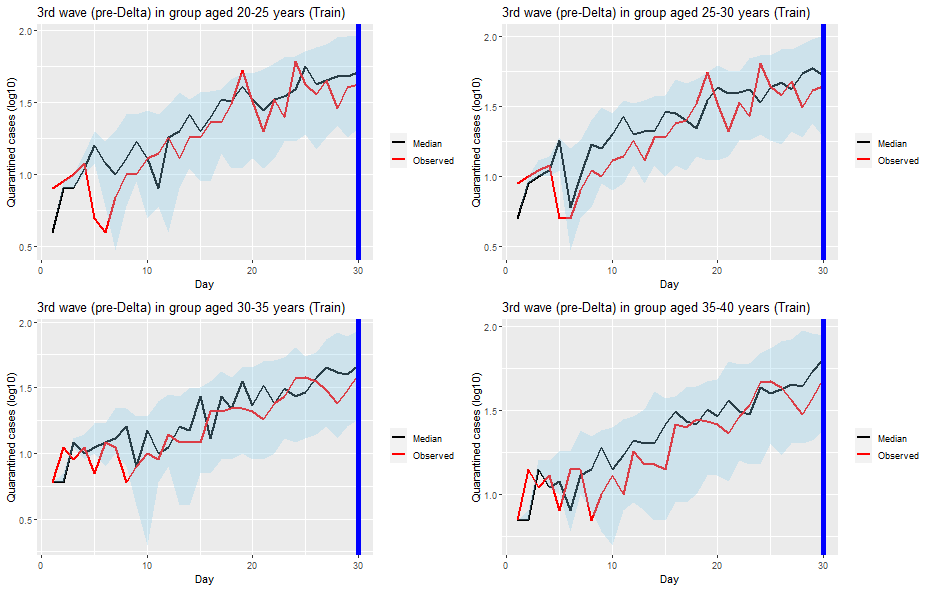


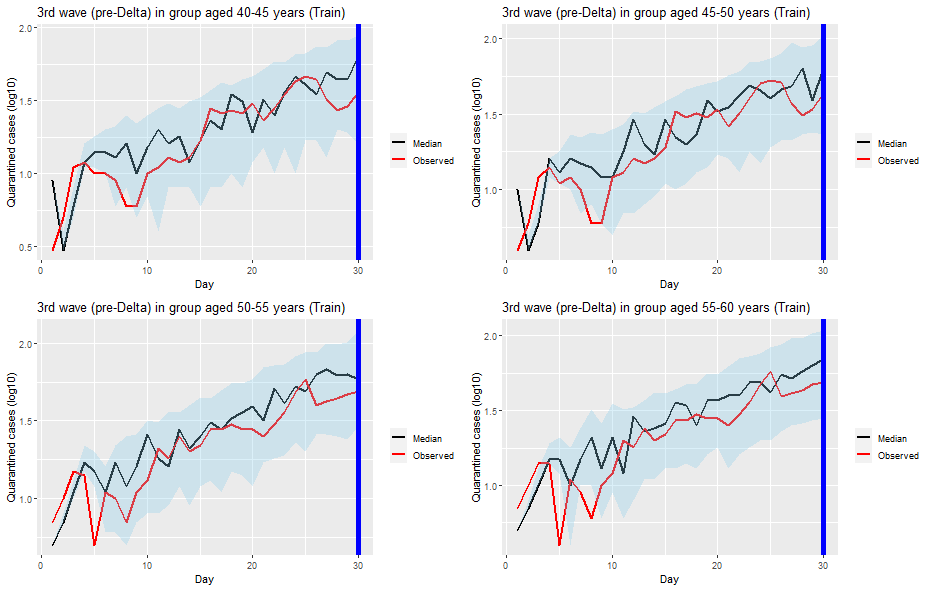


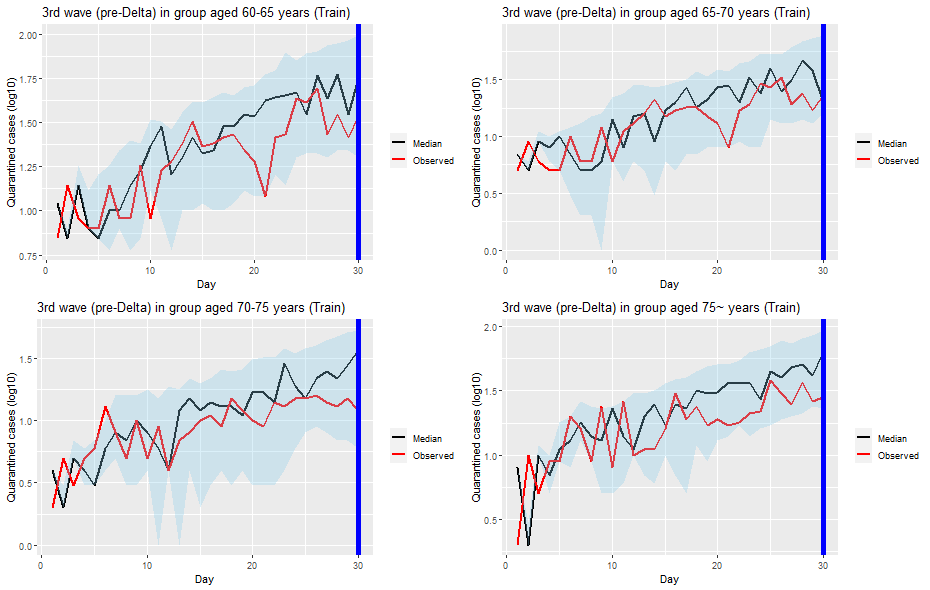


Red line represents observed cases of COVID-19 during the study period, black line represents the median value of simulated cases from the inferred parameters based on the training data, and blue area represents 95% probability interval. The results amply confirm that our model effectively reproduces the age-specific epidemic curves in South Korea.


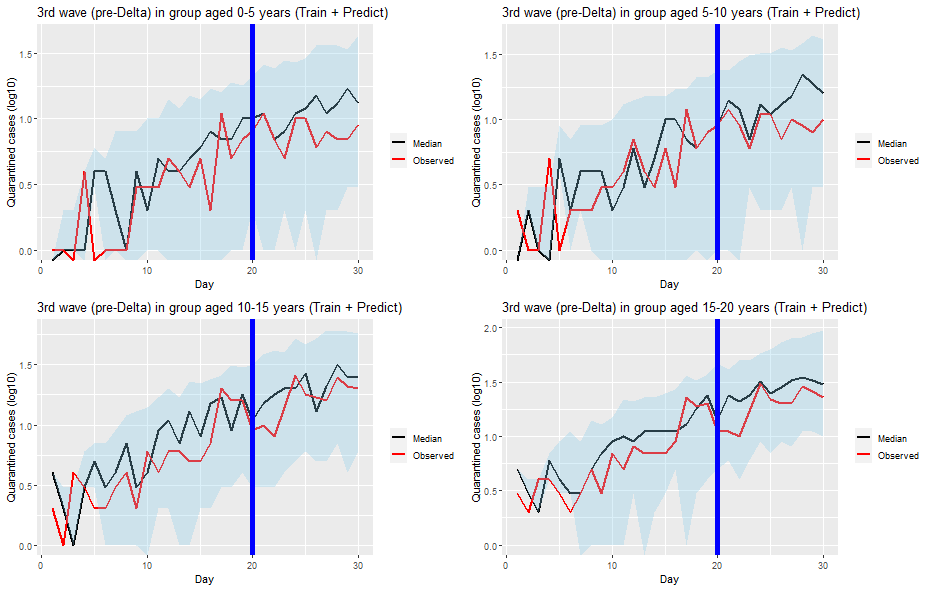


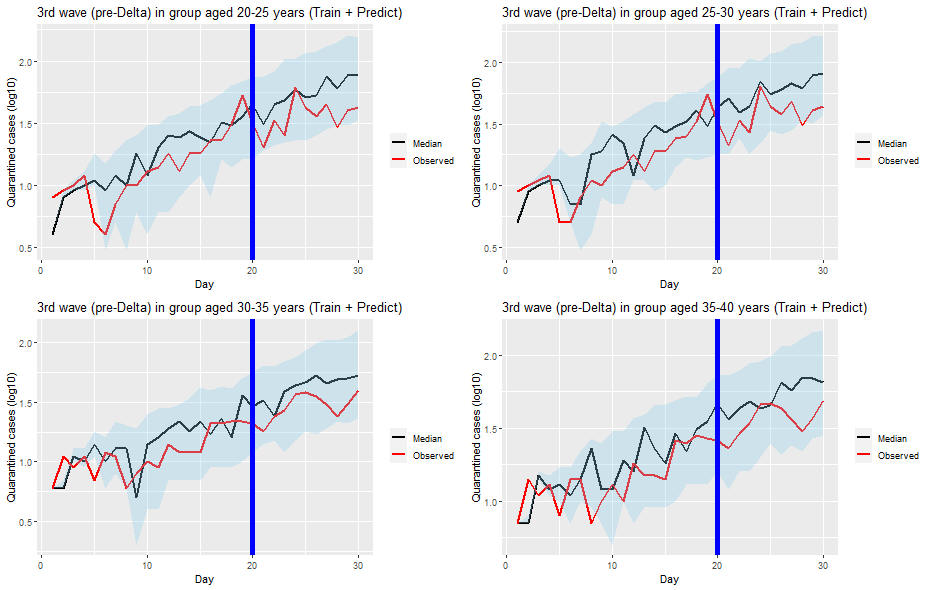


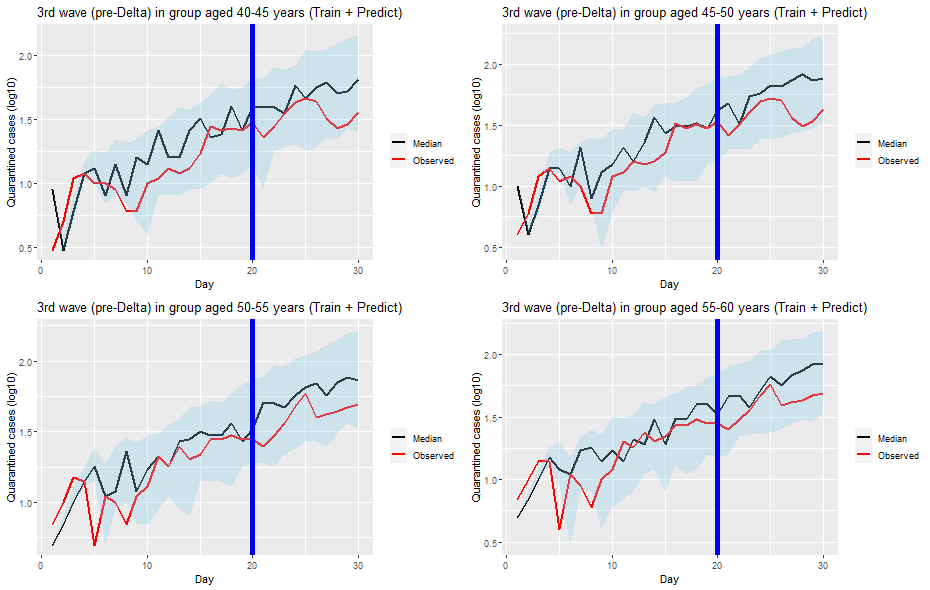


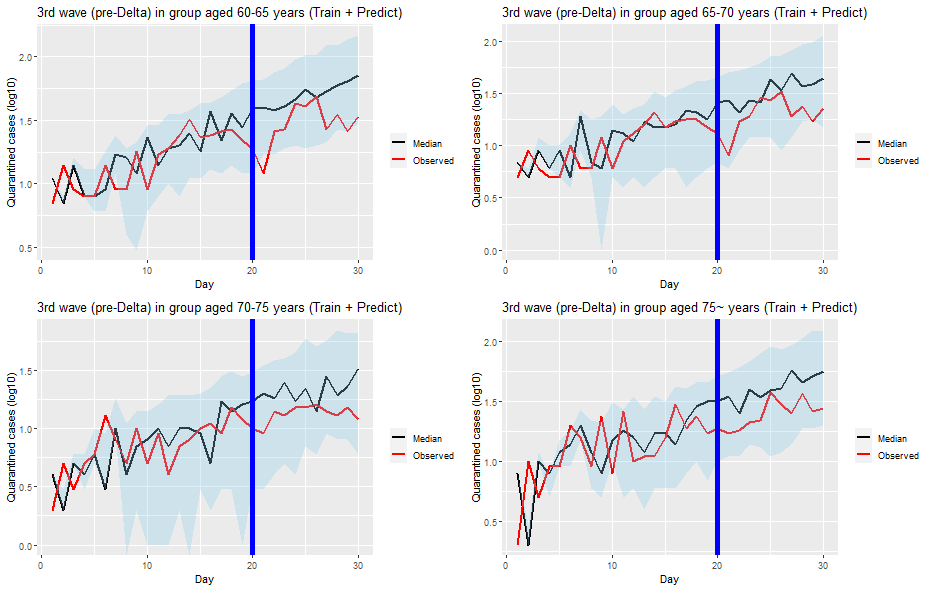


Here, the vertical blue line divides the training period (the period used for estimating the parameters in our model) and the forward prediction period. We tried to see whether the model with inferred parameters could effectively reproduce the age-specific epidemic curves in South Korea beyond the training period (i.e. forward prediction period).

**Fig. S8.** Model validation in 4^th^ wave (Delta)


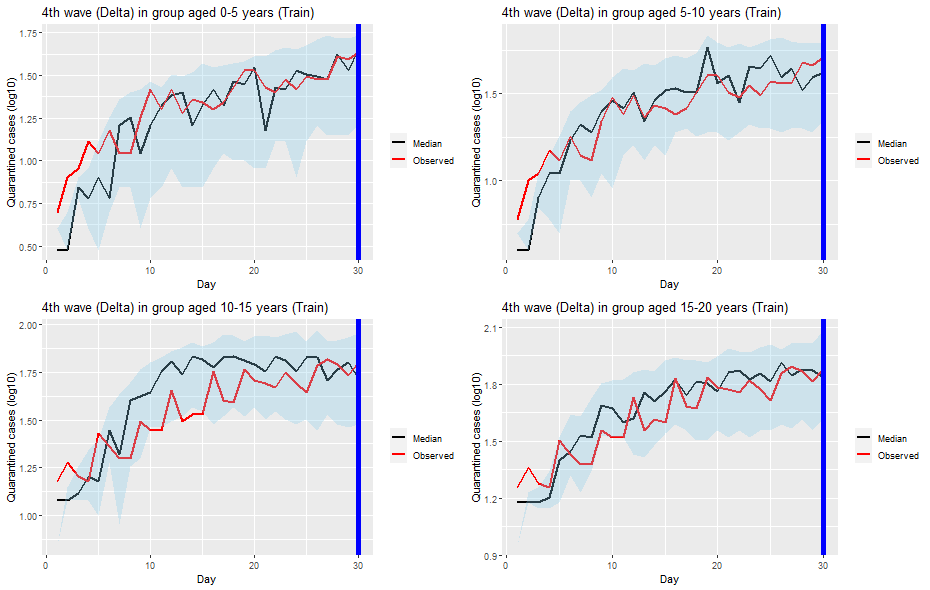


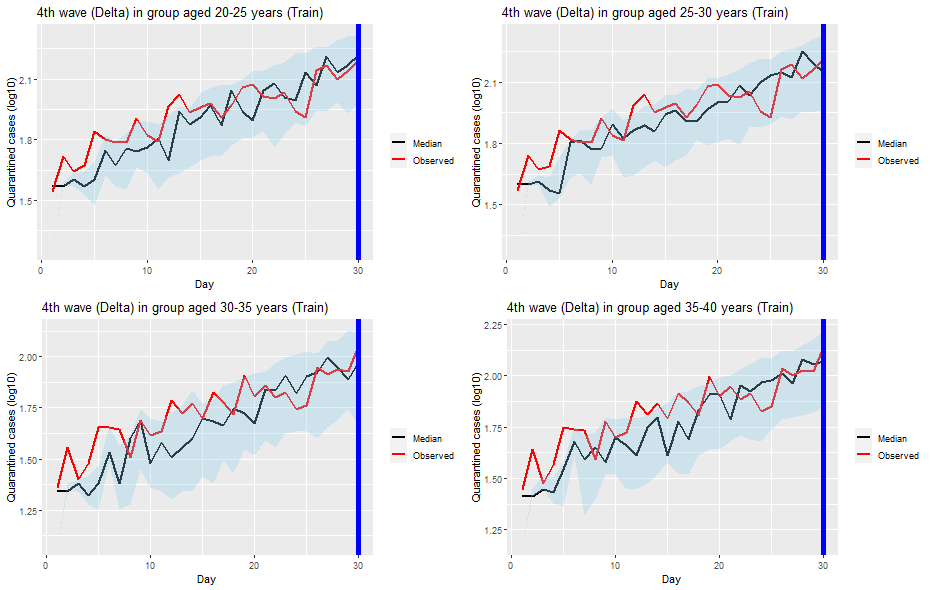


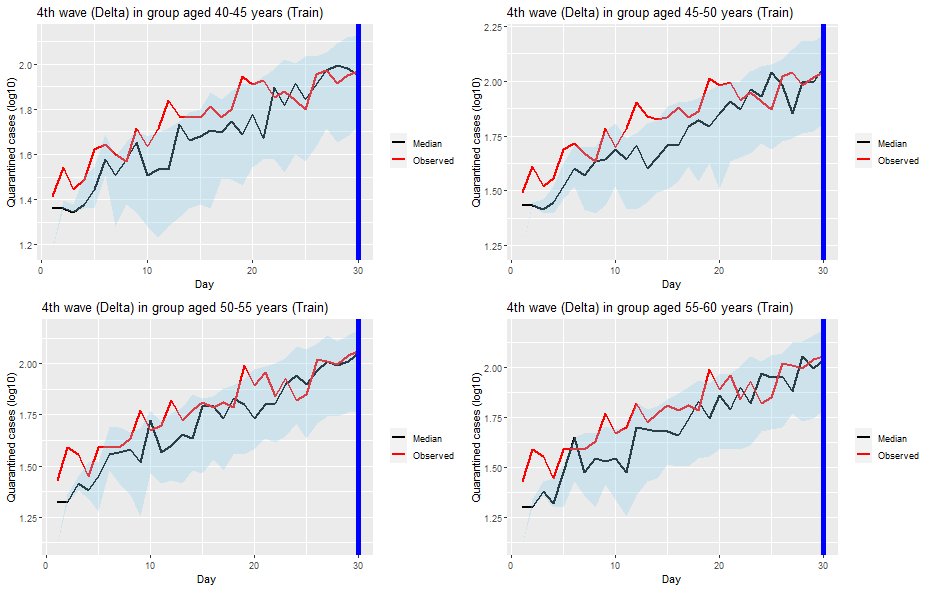


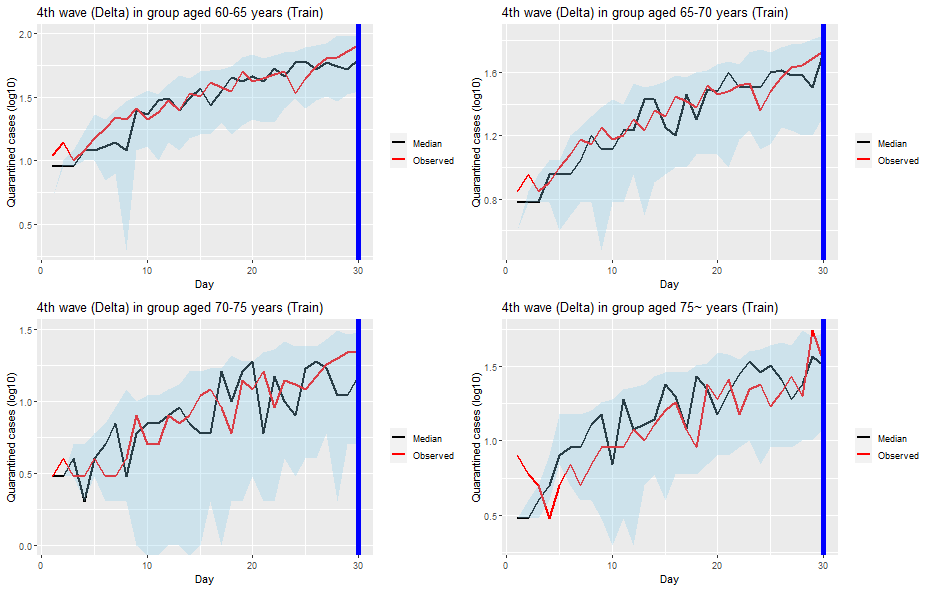


Red line represents observed cases of COVID-19 during the study period, black line represents the median value of simulated cases from the inferred parameters based on the training data, and blue area represents 95% probability interval. The results amply confirm that our model effectively reproduces the age-specific epidemic curves in South Korea.


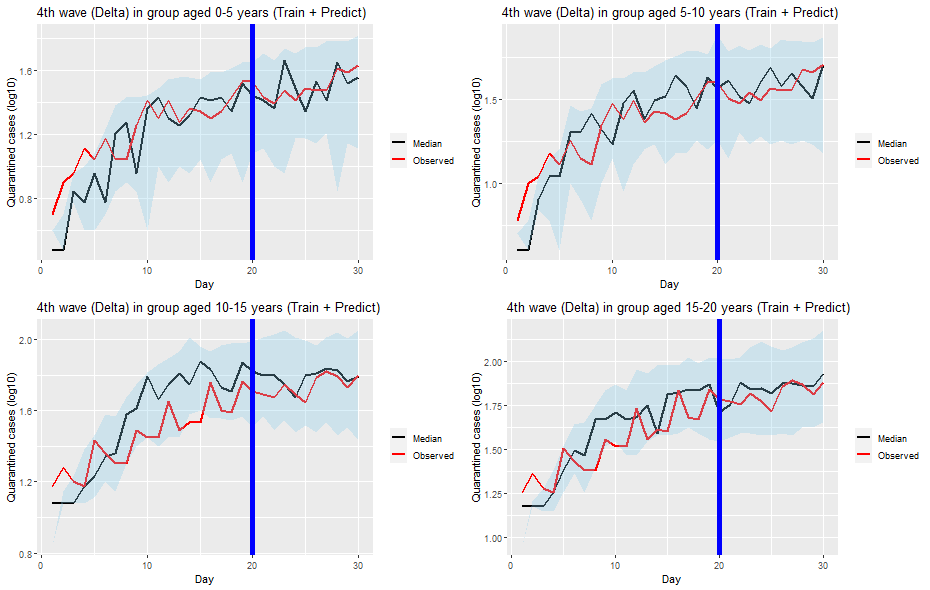


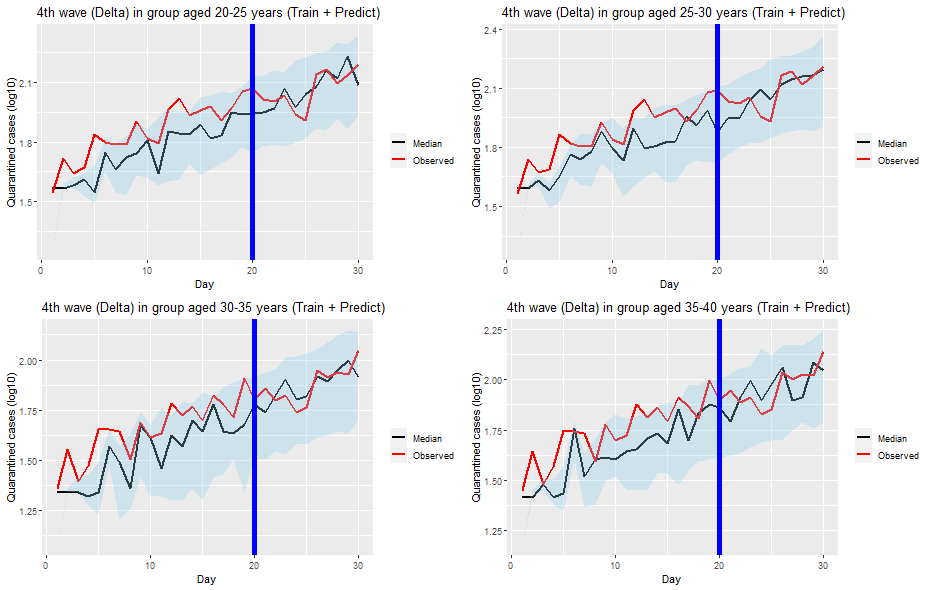


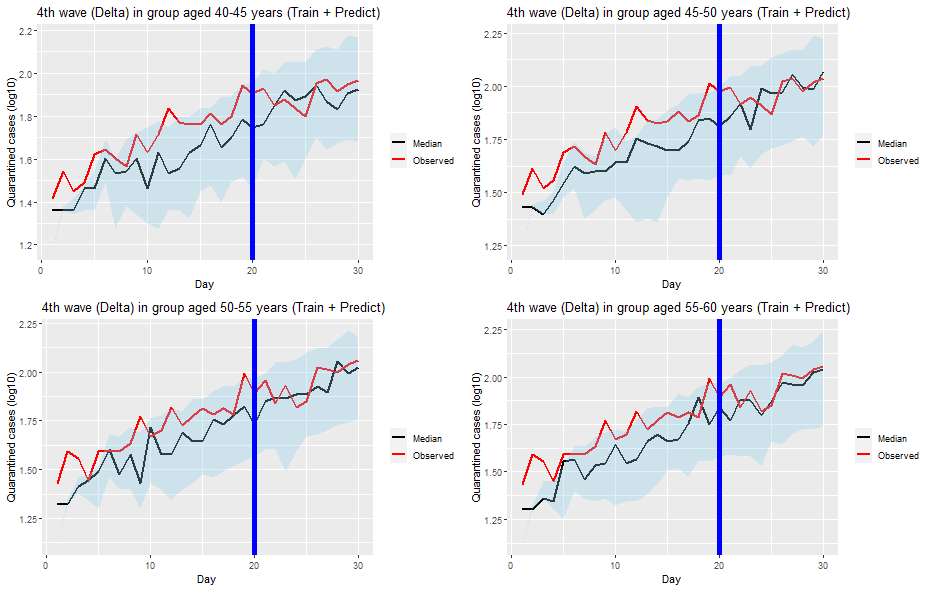


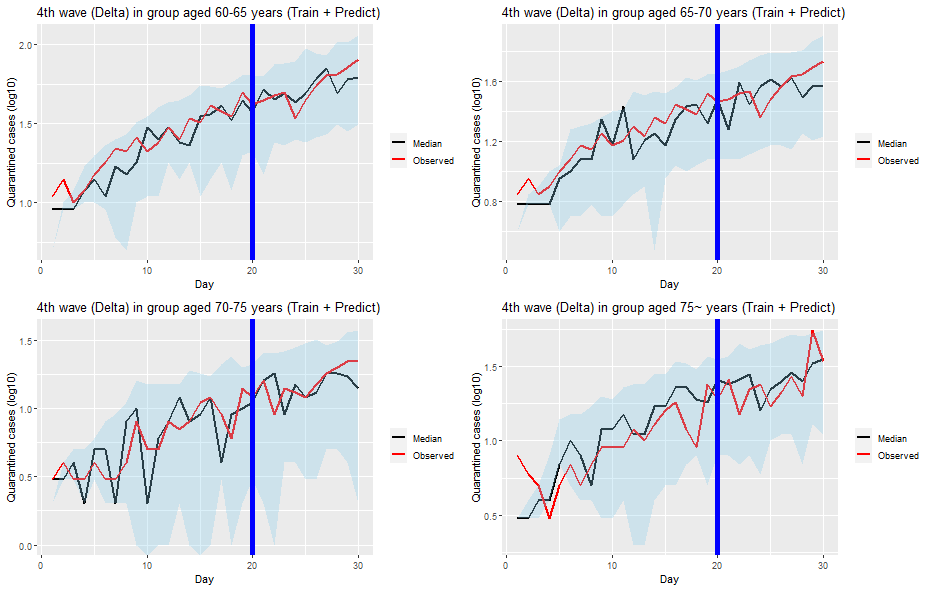


Here, the vertical blue line divides the training period (the period used for estimating the parameters in our model) and the forward prediction period. We tried to see whether the model with inferred parameters could effectively reproduce the age-specific epidemic curves in South Korea beyond the training period (i.e. forward prediction period).

**Fig. S9.** Model validation in 5^th^ wave (Omicron)


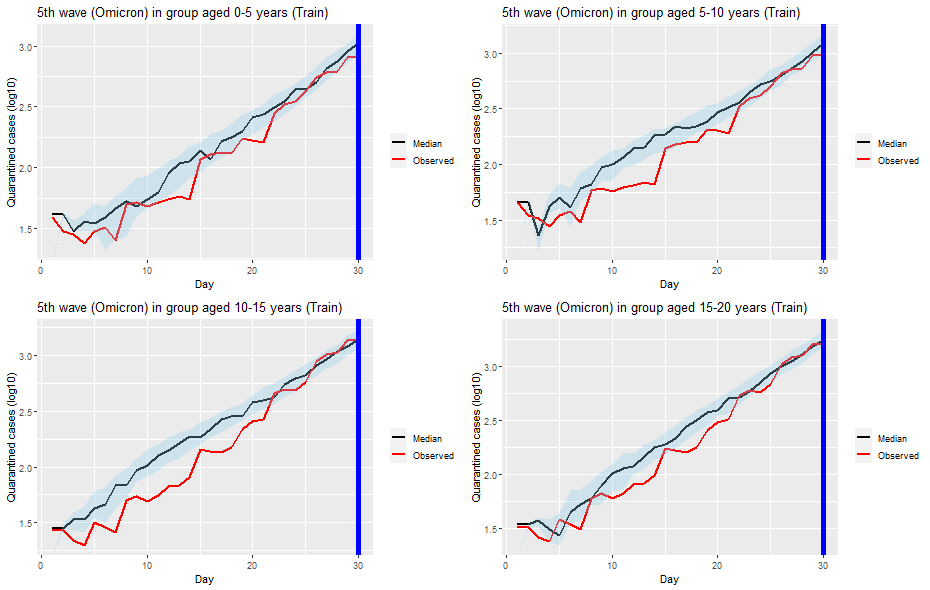


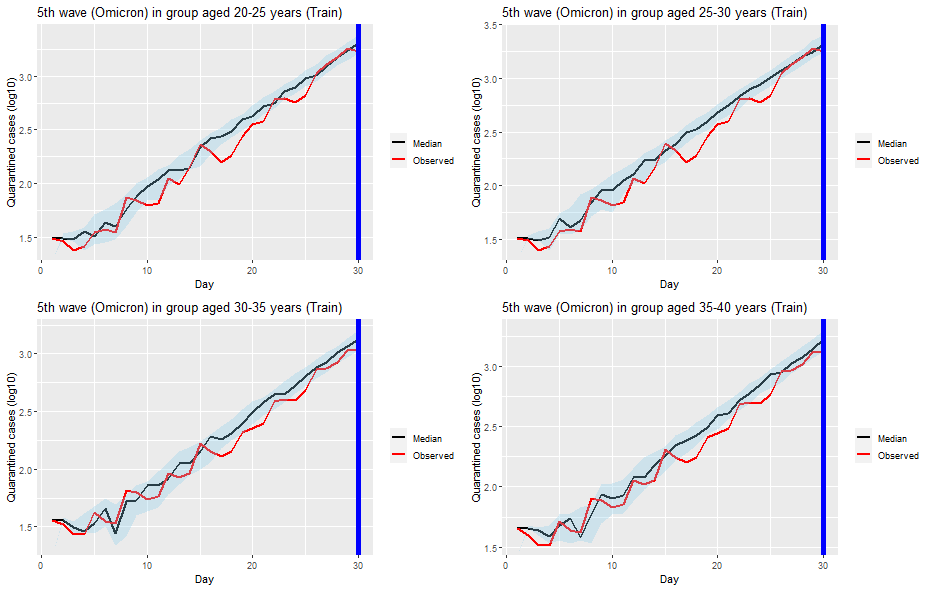


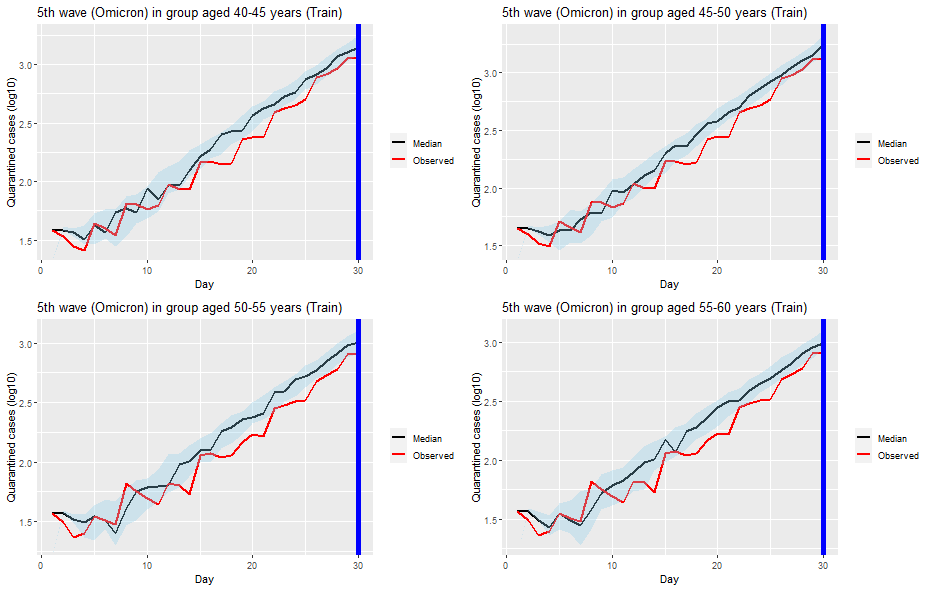


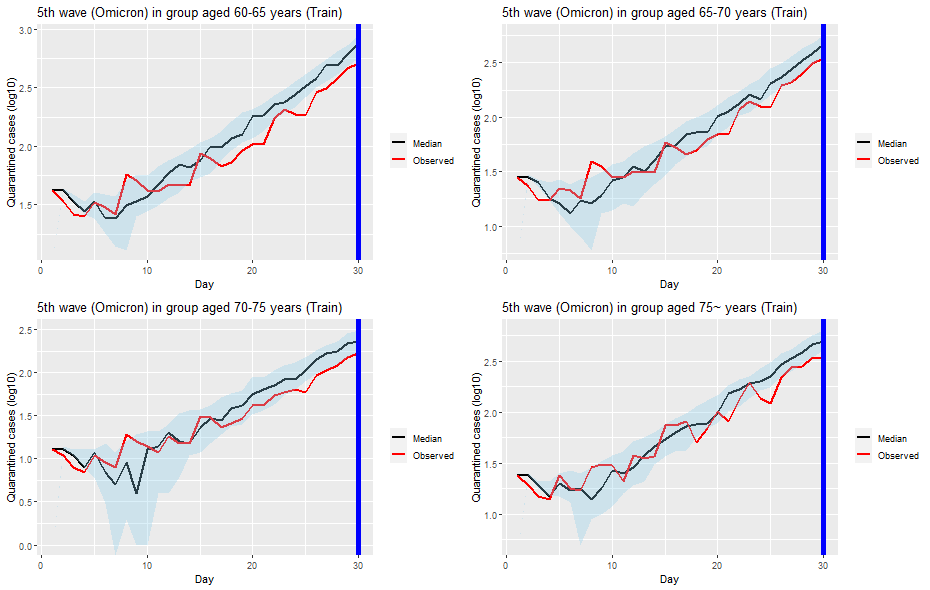


Red line represents observed cases of COVID-19 during the study period, black line represents the median value of simulated cases from the inferred parameters based on the training data, and blue area represents 95% probability interval. The results amply confirm that our model effectively reproduces the age-specific epidemic curves in South Korea.


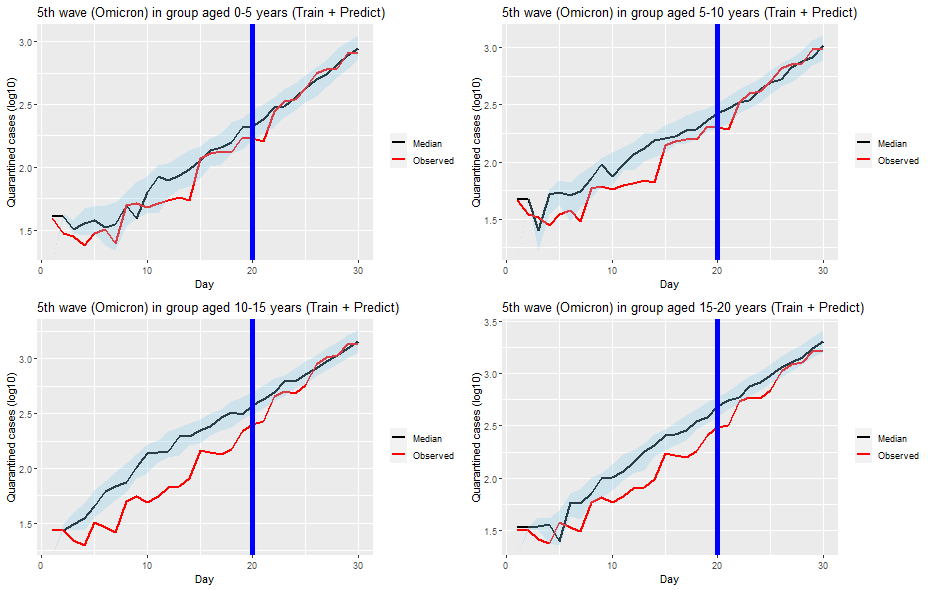


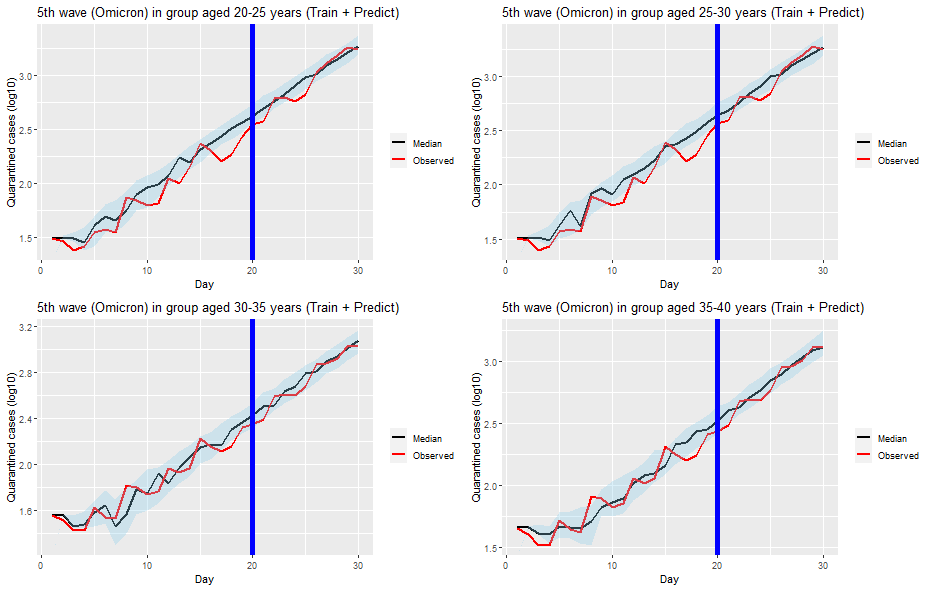


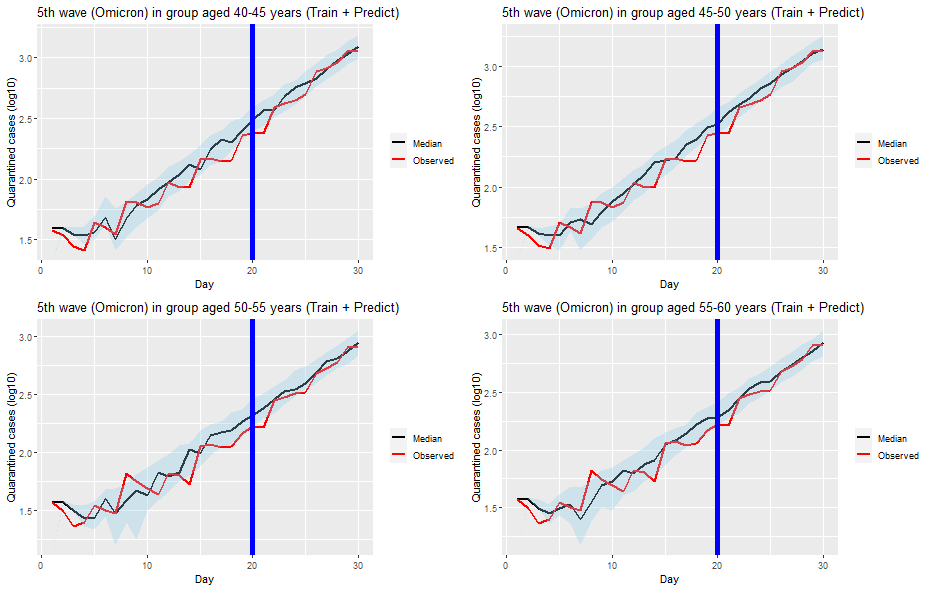


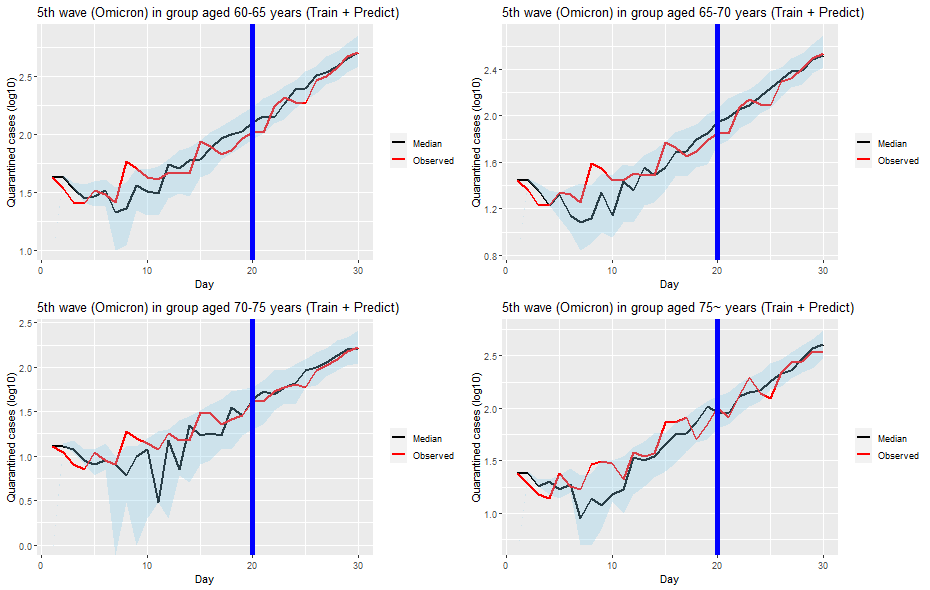


Here, the vertical blue line divides the training period (the period used for estimating the parameters in our model) and the forward prediction period. We tried to see whether the model with inferred parameters could effectively reproduce the age-specific epidemic curves in South Korea beyond the training period (i.e. forward prediction period).

**Fig. S10.** MCMC trace plots and autocorrelation function (ACF) plots in 3^rd^ wave (pre-Delta)


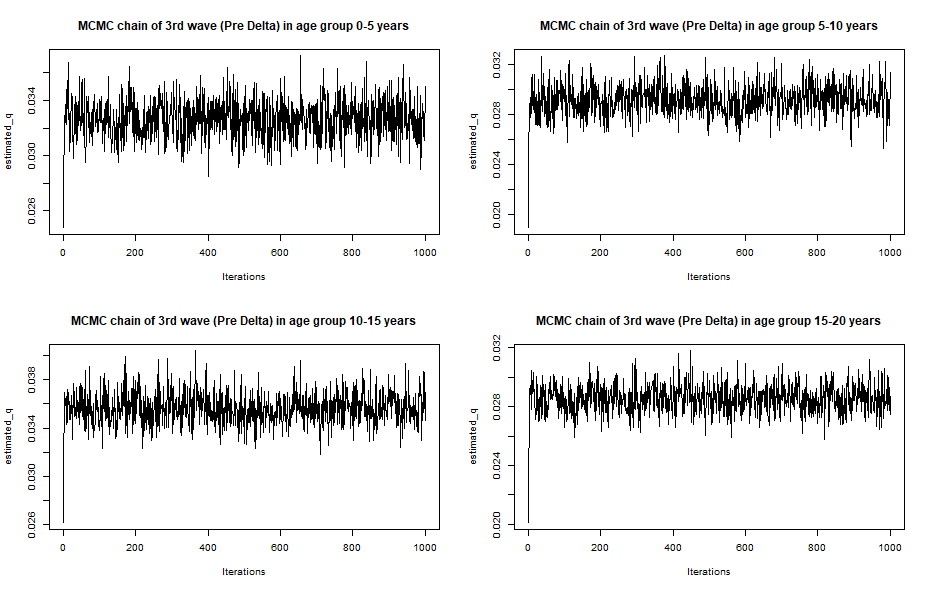


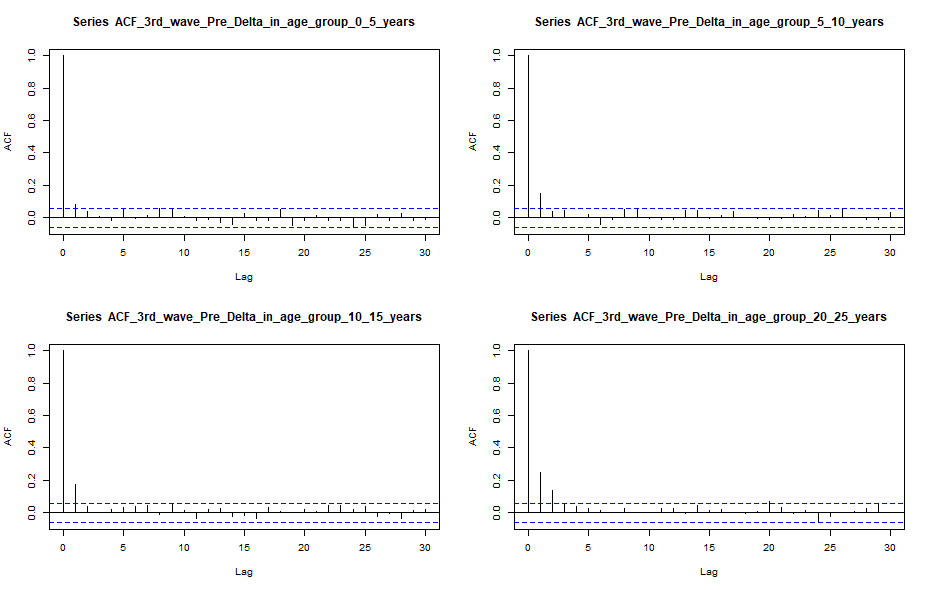


**Fig. S10.** MCMC trace plots and autocorrelation function (ACF) plots in 3^rd^ wave (pre-Delta)


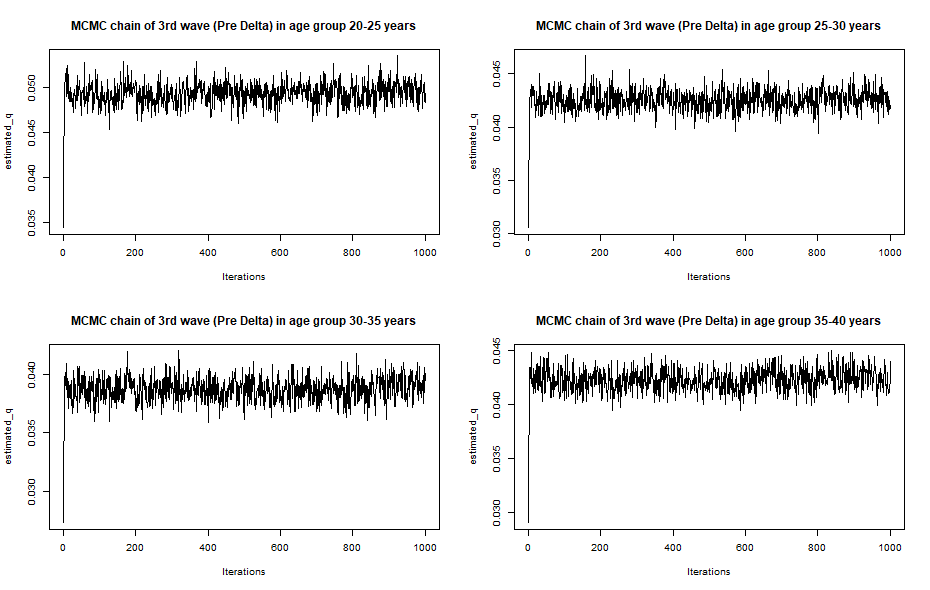


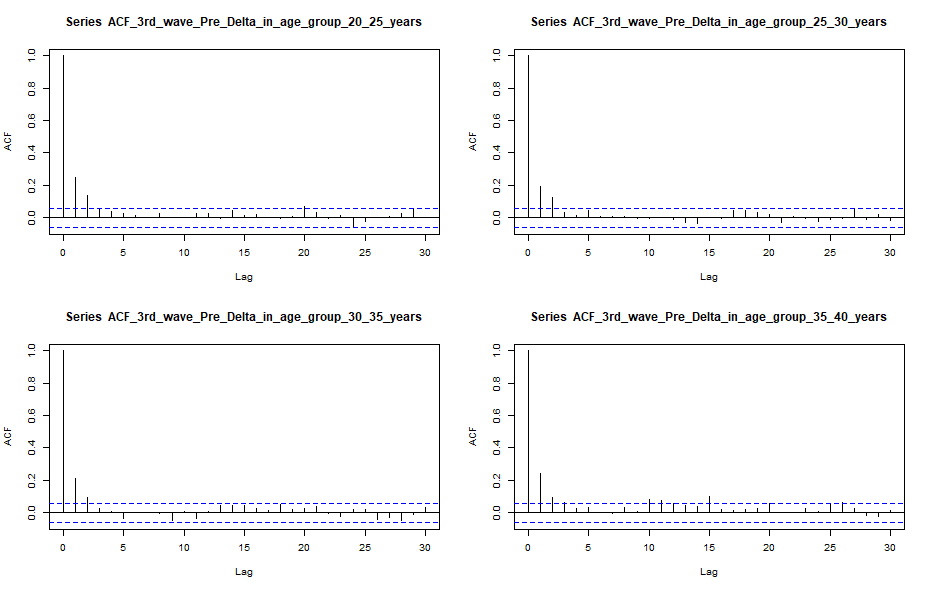


**Fig. S10.** MCMC trace plots and autocorrelation function (ACF) plots in 3^rd^ wave (pre-Delta)


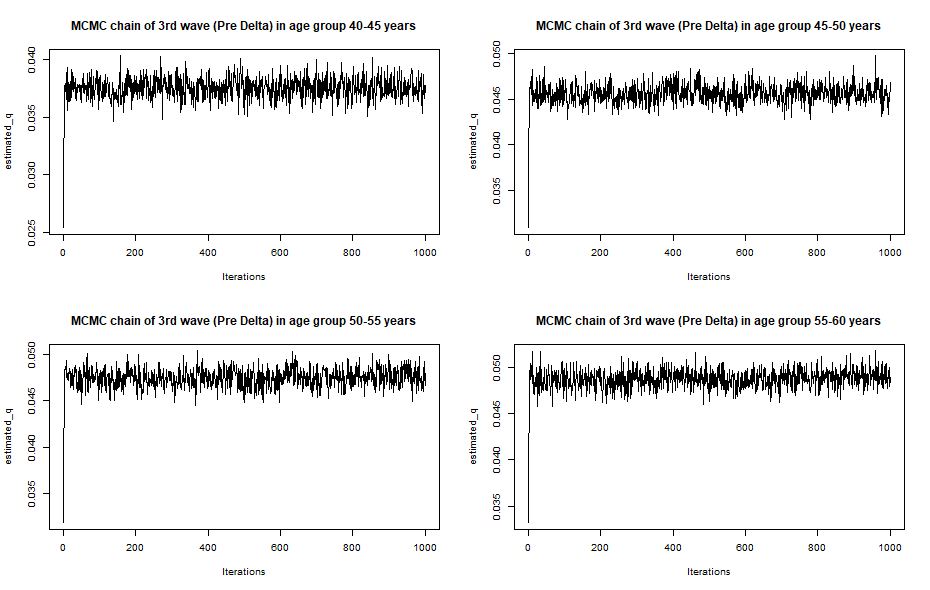


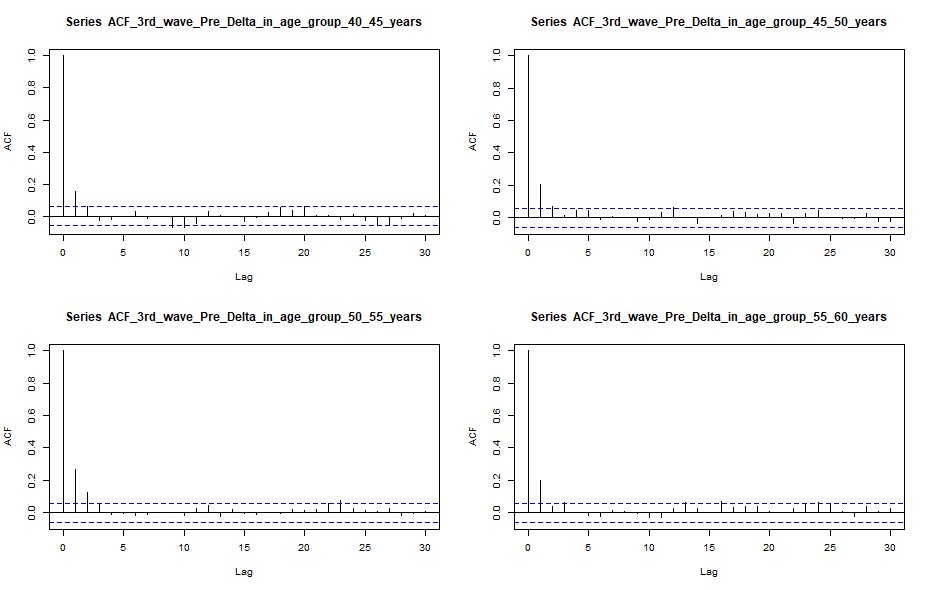


**Fig. S10.** MCMC trace plots and autocorrelation function (ACF) plots in 3^rd^ wave (pre-Delta)


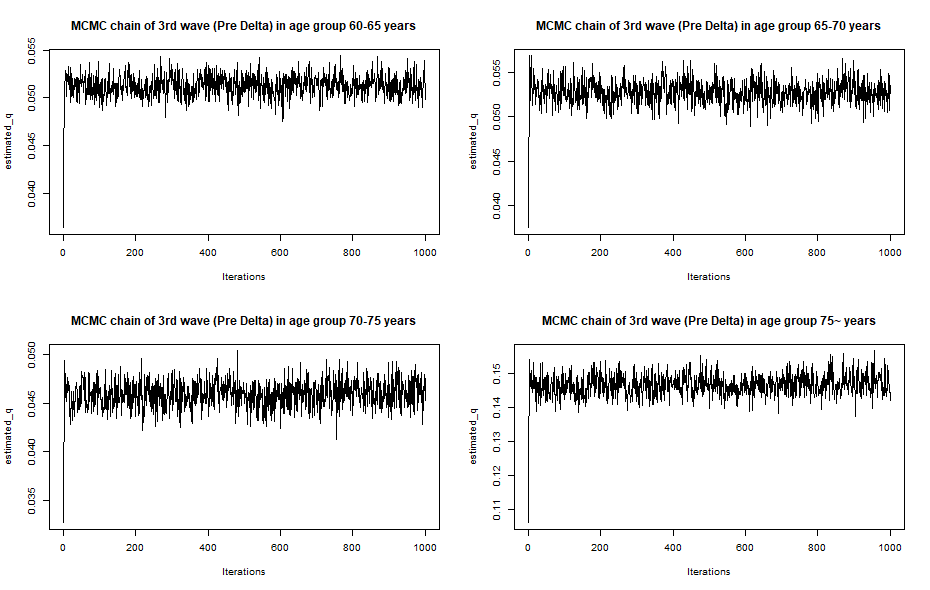


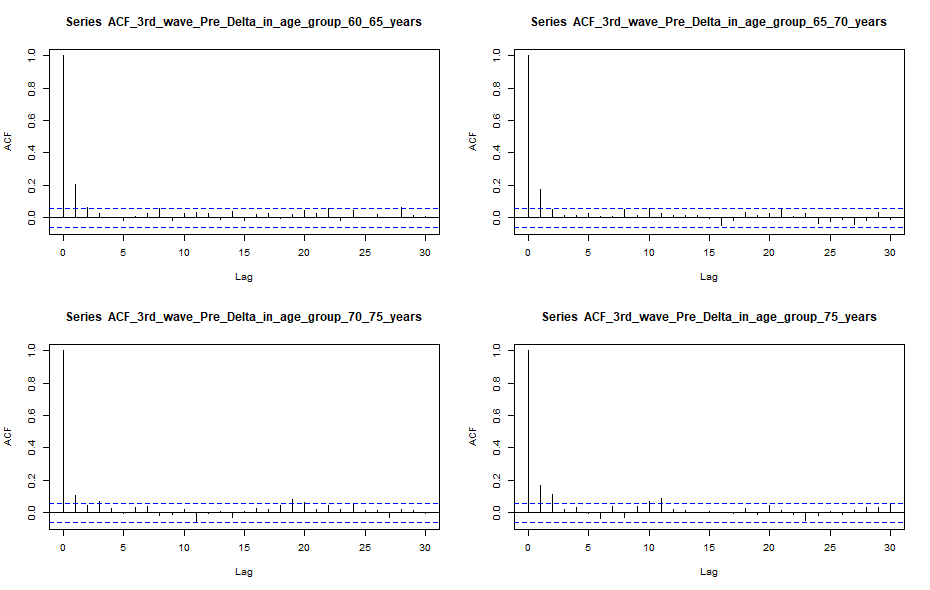


**Fig. S11.** MCMC trace plots and autocorrelation function (ACF) plots in 4^th^ wave (Delta)


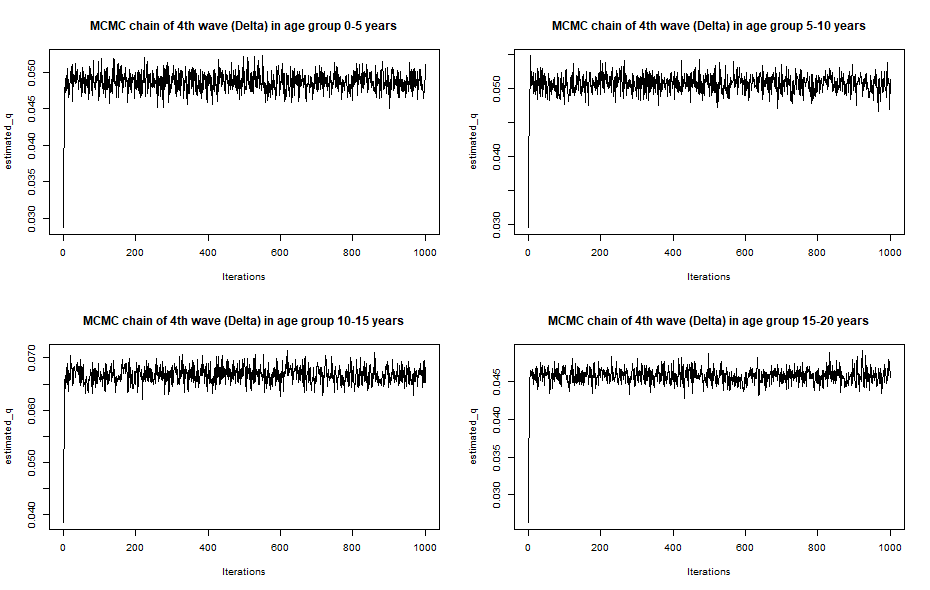


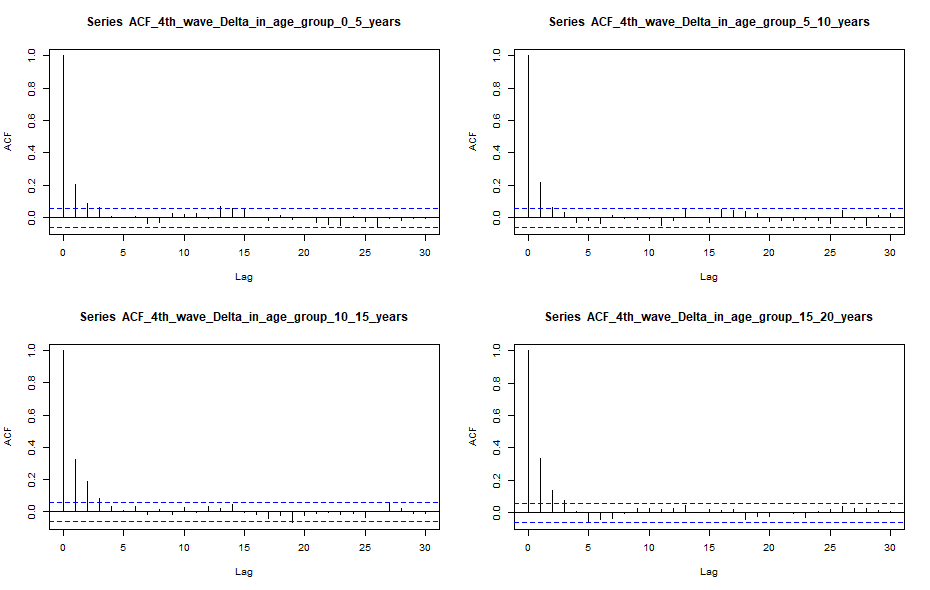


**Fig. S11.** MCMC trace plots and autocorrelation function (ACF) plots in 4^th^ wave (Delta)


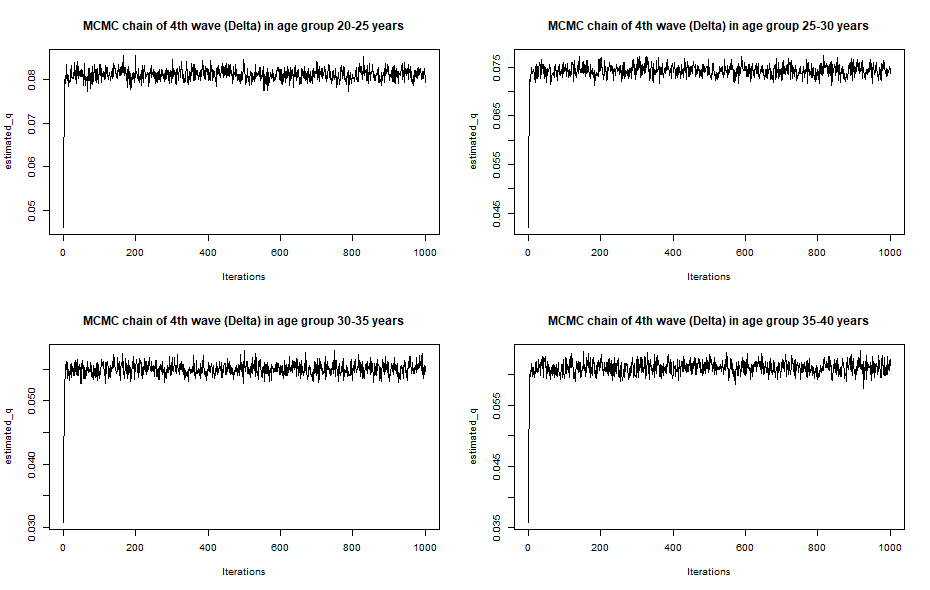


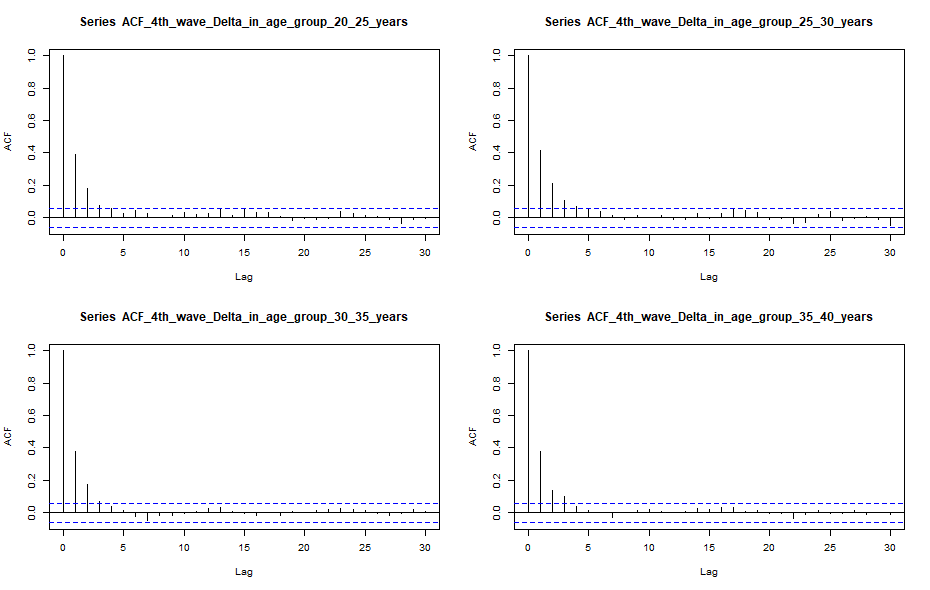


**Fig. S11.** MCMC trace plots and autocorrelation function (ACF) plots in 4^th^ wave (Delta)


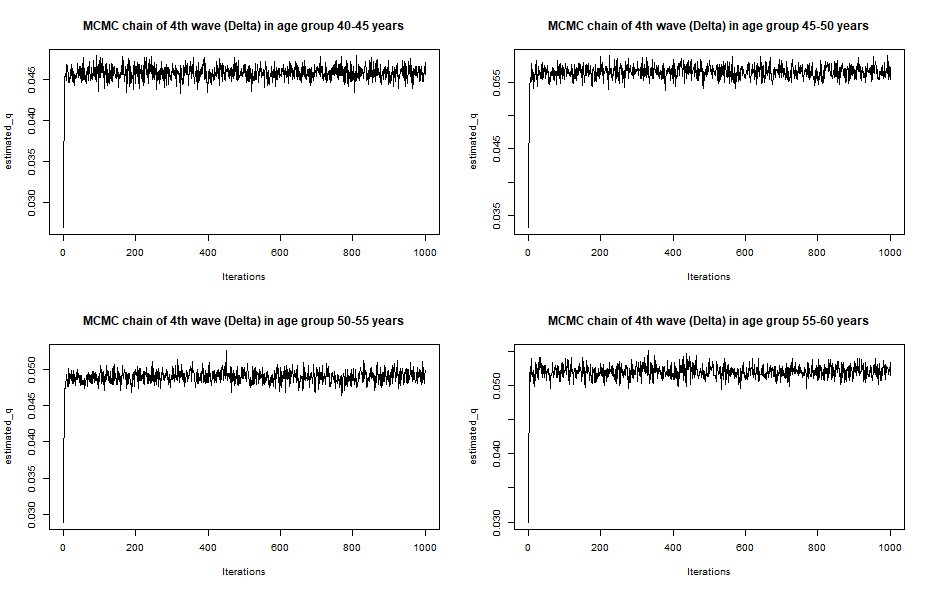


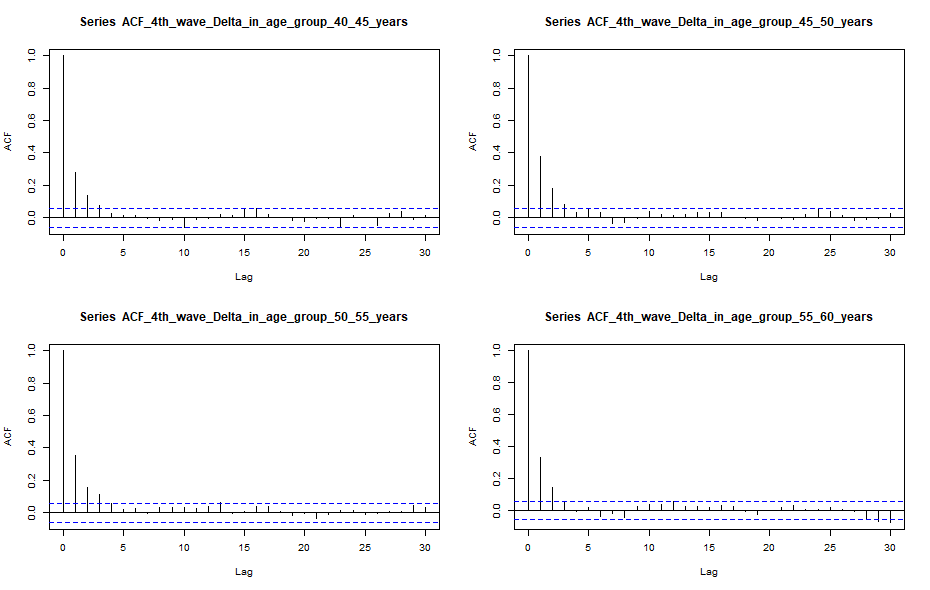


**Fig. S11.** MCMC trace plots and autocorrelation function (ACF) plots in 4^th^ wave (Delta)


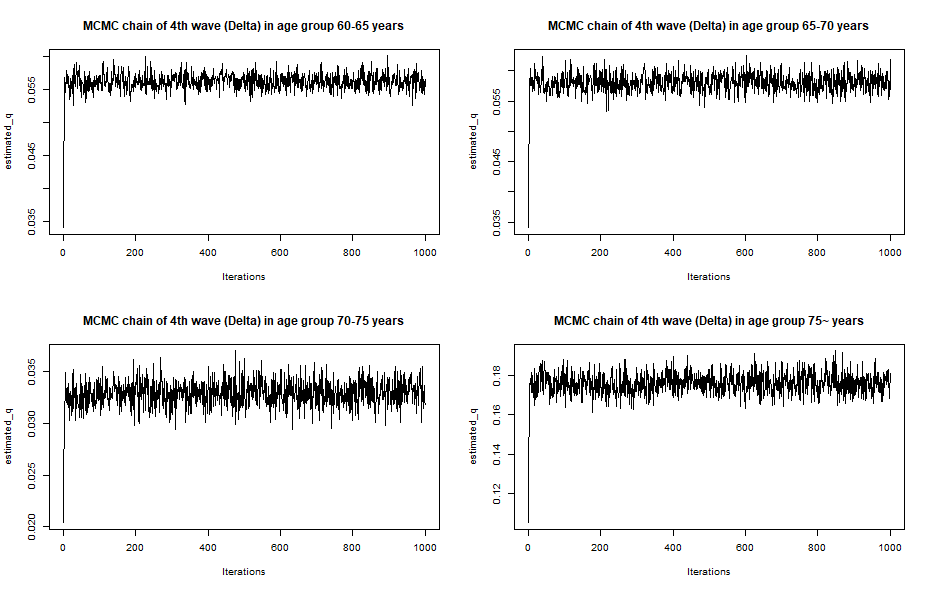


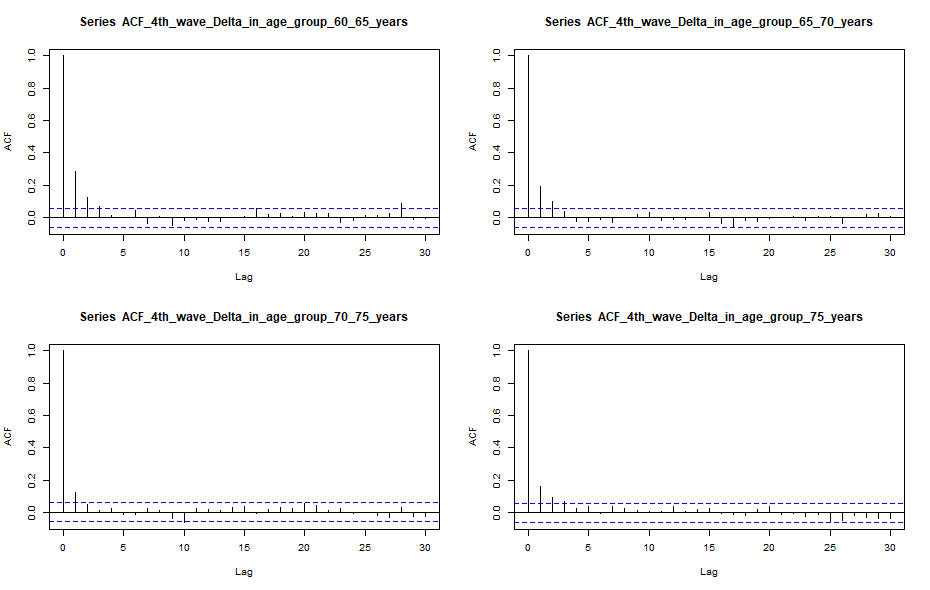


**Fig. S12.** MCMC trace plots and autocorrelation function (ACF) plots in 5^th^ wave (Omicron)


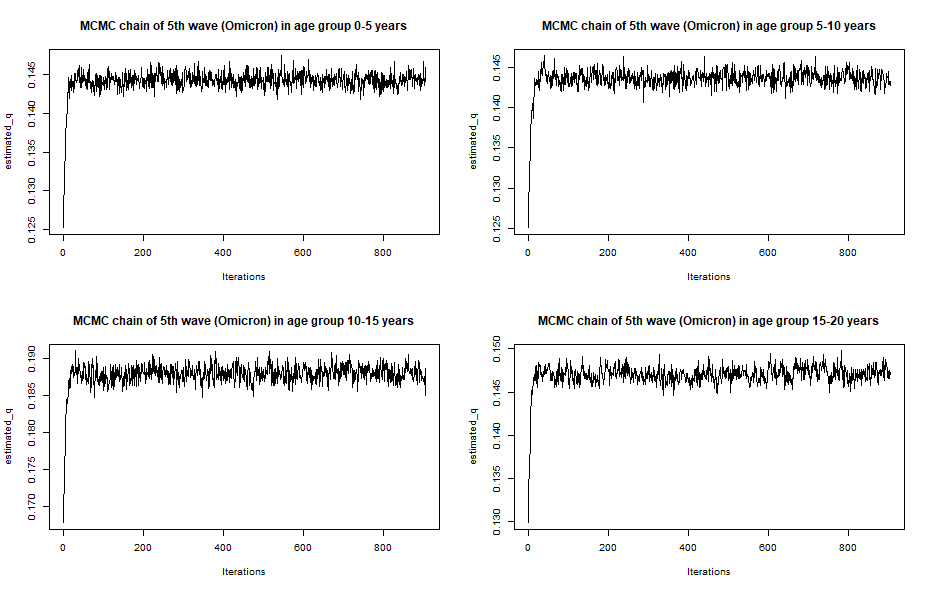


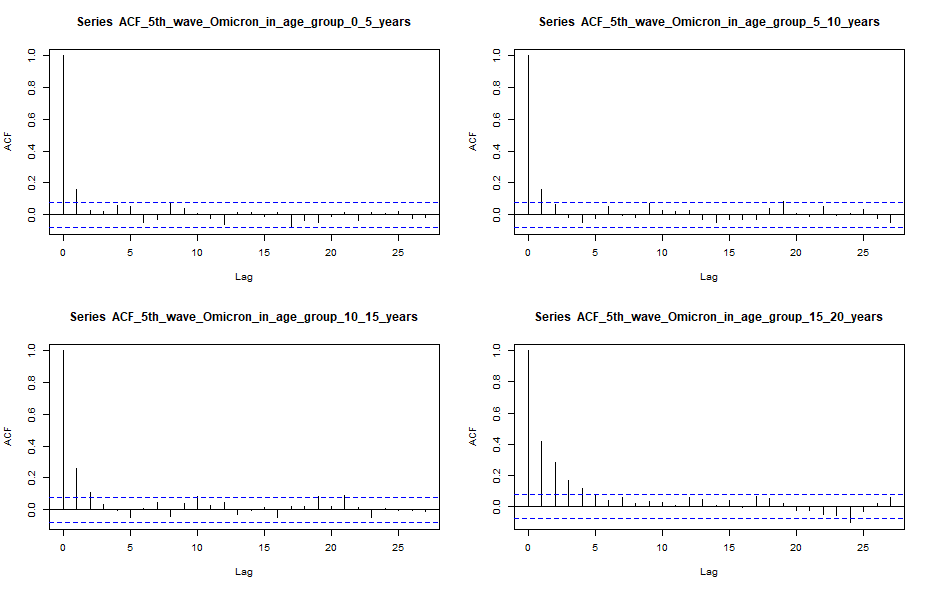


**Fig. S12.** MCMC trace plots and autocorrelation function (ACF) plots in 5^th^ wave (Omicron)


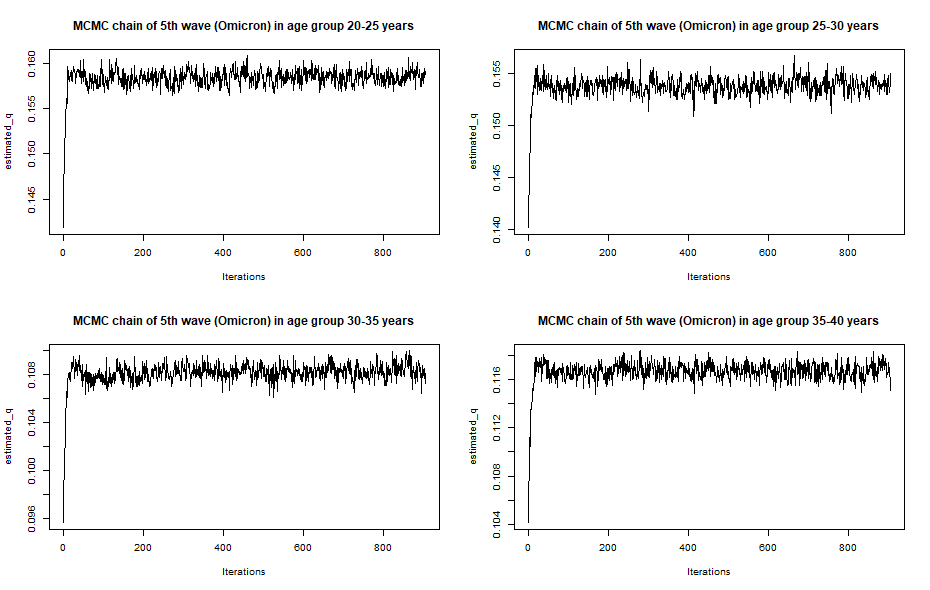


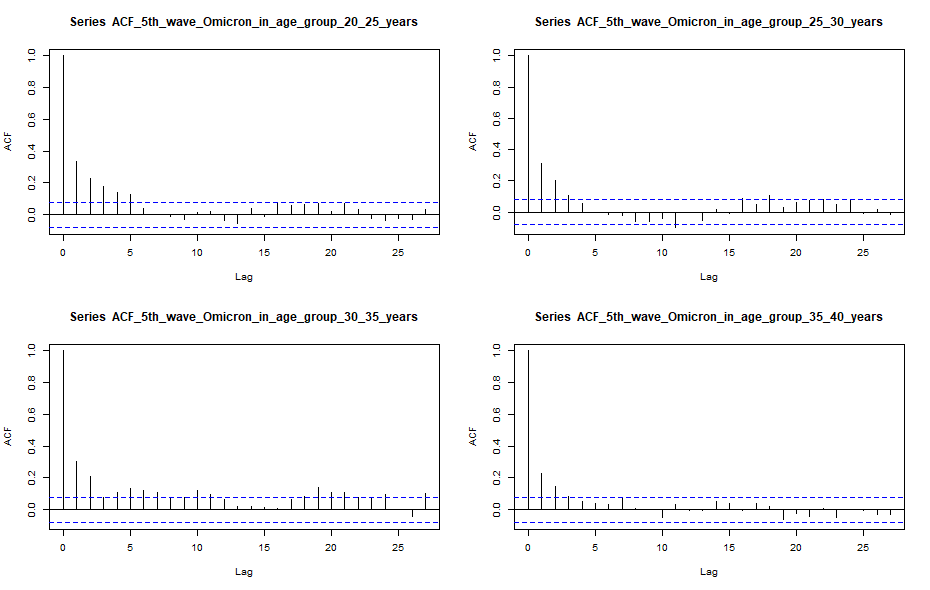


**Fig. S12.** MCMC trace plots and autocorrelation function (ACF) plots in 5^th^ wave (Omicron)


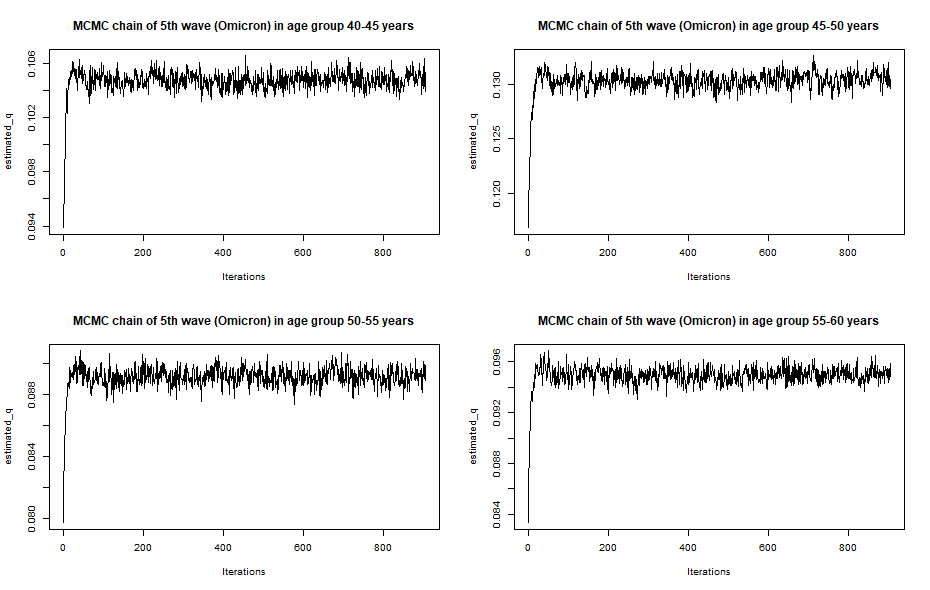


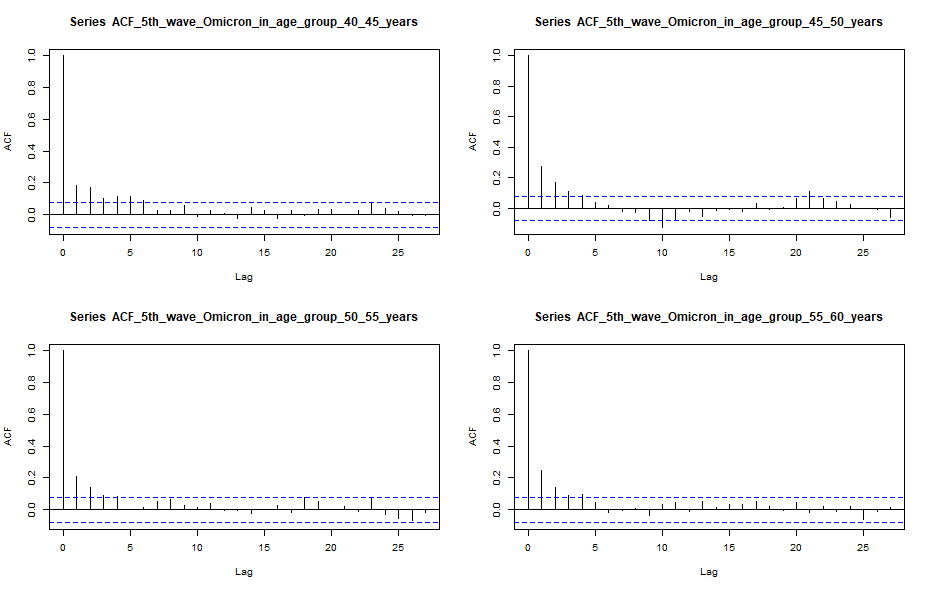


**Fig. S12.** MCMC trace plots and autocorrelation function (ACF) plots in 5^th^ wave (Omicron)


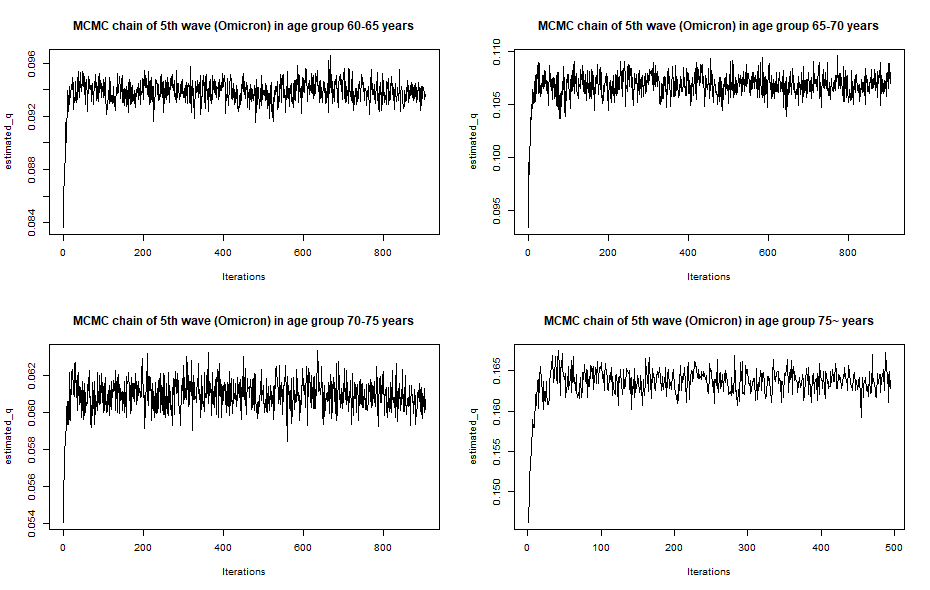


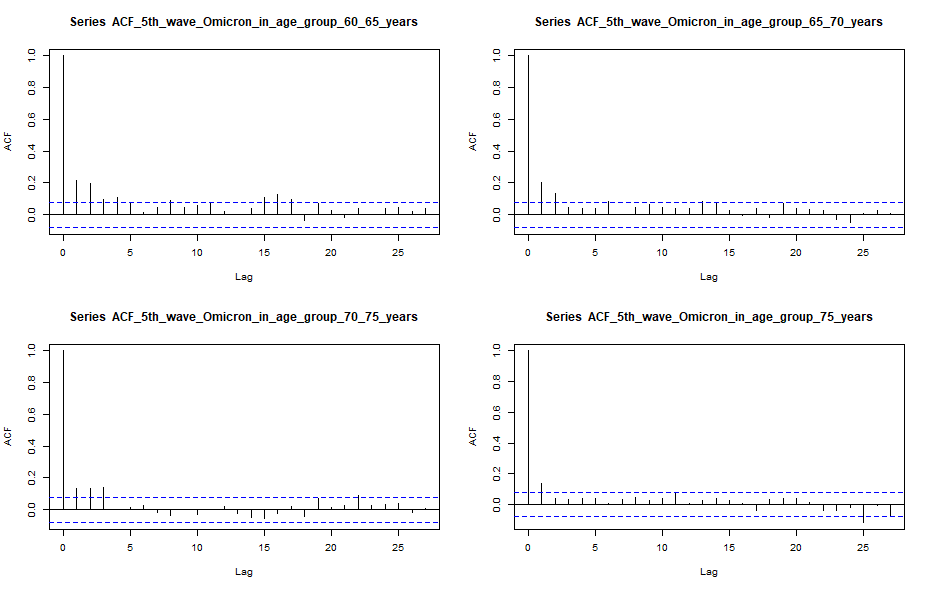


**Table S1.** Overview of Social Distancing System in South Korea

▷ 5 Tier Social distancing system (Nov 7, 2020 to July 11, 2021)

| Classification | Level 1 | Level 1.5 | Level 2 | Level 2.5 | Level 3 |
| --- | --- | --- | --- | --- | --- |
| Definition | Preventive measures | Local Transmission | | Nationwide Transmission | |
| Criteria  (weekly average) | (Capital) < 100 cases  (Others) < 30 cases | (Capital) ≥ 100 cases  (Others) ≥ 30 cases | Nationwide ≥ 300 cases | Nationwide 400–500+ | Nationwide 800–1,000+ |
| Events | Basic quarantine measures mandatory (wearing masks, registration list for gathering) | No. of people limited to 1 per 4m^2^ area | 100+ people prohibited | 50+ people prohibited | Only for family gatherings |
| School | 2/3 density | 2/3 density | 2/3 (high school) 1/3 (otherwise) | 1/3 density | Remote learning |
| Workplace | Recommend tele-working | | | Highly recommend tele-working for 1/3 of employees | Highly recommend tele-working except essential workers |

▷ 4 Tier Social distancing system (July 12, 2021 to Oct 31, 2021)

| Classification | Level 1 | Level 2 | Level 3 | Level 4 |
| --- | --- | --- | --- | --- |
| Definition | Contained and Stable | Local Transmission | Regional Transmission | Full-Blown Nationwide Transmission |
| Criteria | < 1 case per 100,000 people (weekly average) | ≥ 1 case per 100,000 people (weekly average > threshold for 3+ days) | ≥ 2 cases per 100,000 people (weekly average > threshold for 3+ days) | ≥ 4 cases per 100,000 people (weekly average > threshold for 3+ days) |
| Private gatherings | Comply with COVID-19 protocols | Up to 8 people (gatherings of 9+ prohibited) | Up to 4 people (gatherings of 5+ prohibited) | Up to 2 people after 18:00 (gatherings of 3+ prohibited)  ^※^ Private gatherings of up to 4 persons permitted until 18:00 |
|  | - Fully/partially vaccinated people not counted for immediate family gatherings - Fully vaccinated people not counted for private gatherings (except Level 4) | | | |
| Events | 500+ people only with advance reporting to local authorities | 100+ people prohibited | 50+ people prohibited | Events prohibited |
|  | - Fully vaccinated people not counted for events | | | |
| School | Comply with basic health protocols, full-time in-person classes permitted | | | Full-time (high school) 2/3 (otherwise) |
| Workplace | - | Recommend tele-working for 10% of employees | Recommend tele-working for 20% of employees | Recommend tele-working for 30% of employees |

Reference: http://ncov.mohw.go.kr/

**Table S2.** School Attendance Ratio (%) during the 4^th^ and 5^th^ waves

▷ **5^th^ wave**

| Attendance ratio (%) | 2022-04-04 | 2022-03-28 | 2022-03-21 | 2022-03-14 | 2022-03-07 | 2022-03-02 | 2022-02-22 | 2022-02-15 | 2022-02-08 |
| --- | --- | --- | --- | --- | --- | --- | --- | --- | --- |
| Kindergarten | 85.6 | 81.1 | 78.3 | 78.5 | 77.8 | 62.6 | 1.6 | 14 | 27.5 |
| Primary school | 90.2 | 86.2 | 84.2 | 83.2 | 81.3 | 88.1 | 0.2 | 3.2 | 4.1 |
| Middle school | 90.8 | 83.8 | 85.3 | 85.6 | 81.8 | 94 | 2.1 | 27.7 | 10.3 |
| High school | 91.9 | 90.4 | 88 | 86.2 | 85.2 | 93.5 | 70.1 | 55.9 | 6.5 |

| Attendance ratio (%) | 2022-02-01 | 2022-01-25 | 2022-01-18 | 2022-01-11 | 2022-01-04 | 2021-12-28 | 2021-12-21 |
| --- | --- | --- | --- | --- | --- | --- | --- |
| Kindergarten | 20.4 | 15.2 | 1.6 | 20.2 | 45.8 | 78 | 82.7 |
| Primary school | 0.2 | 0.1 | 0 | 3.2 | 31.5 | 58.6 | 82.8 |
| Middle school | 0.9 | 0.6 | 0.1 | 0.4 | 10.3 | 50.5 | 77.7 |
| High school | 5.2 | 4.2 | 0.9 | 0.8 | 10 | 50.2 | 69.2 |

▷ **4^th^ wave**

| Attendance ratio (%) | 2021-09-29 | 2021-09-28 | 2021-09-23 | 2021-09-15 | 2021-09-13 | 2021-09-09 | 2021-09-06 | 2021-09-01 | 2021-08-31 |
| --- | --- | --- | --- | --- | --- | --- | --- | --- | --- |
| Kindergarten | 91.6 | 90.9 | 89.8 | 93.2 | 91.3 | 92.3 | 90.3 | 88.9 | 82.3 |
| Primary school | 79.7 | 78.7 | 75.6 | 79.3 | 77.9 | 77.7 | 76.4 | 55.7 | 48.6 |
| Middle school | 80.5 | 80.3 | 76.5 | 79.9 | 79.7 | 76.9 | 74 | 45 | 56.2 |
| High school | 81.4 | 81.7 | 72.9 | 81 | 80.9 | 80.9 | 81 | 73.5 | 74.2 |

| Attendance ratio (%) | 2021-08-26 | 2021-08-24 | 2021-08-19 | 2021-08-13 | 2021-08-11 | 2021-08-09 | 2021-08-04 | 2021-08-02 | 2021-07-28 |
| --- | --- | --- | --- | --- | --- | --- | --- | --- | --- |
| Kindergarten | 74.4 | 66.5 | 55.7 | 27.5 | 20.4 | 15.2 | 1.9 | 1.6 | 11.7 |
| Primary school | 36.7 | 20.6 | 4.1 | 0.4 | 0.2 | 0.1 | 0 | 0 | 0.7 |
| Middle school | 55.7 | 50.7 | 27.7 | 2.1 | 0.9 | 0.6 | 0.2 | 0.1 | 0 |
| High school | 73.1 | 70.1 | 55.9 | 6.5 | 5.2 | 4.2 | 1.1 | 0.9 | 0.4 |

| Attendance ratio (%) | 2021-07-26 | 2021-07-21 | 2021-07-16 | 2021-07-14 | 2021-07-12 | 2021-07-07 | 2021-06-30 | 2021-06-23 | 2021-06-16 |
| --- | --- | --- | --- | --- | --- | --- | --- | --- | --- |
| Kindergarten | 20.2 | 42.8 | 45.8 | 56.7 | 78 | 94.3 | 94.3 | 95.2 | 94.4 |
| Primary school | 3.2 | 24.5 | 31.5 | 46.9 | 58.6 | 80.2 | 79.9 | 80.2 | 79.3 |
| Middle school | 0.4 | 4.2 | 10.3 | 45.3 | 50.5 | 77.6 | 76.6 | 76.3 | 76 |
| High school | 0.8 | 3.4 | 10 | 47.3 | 57.6 | 79.4 | 85.6 | 76.2 | 75.7 |

Reference: <https://www.moe.go.kr/boardCnts/listRenew.do?boardID=72754&m=031302&s=moe>

**Table S3.** Vaccine coverage data in South Korea

| **Age** | **Vaccine** | **Dose** | **6-Mar-21** | **13-Mar-21** | **20-Mar-21** | **27-Mar-21** | **3-Apr-21** | **10-Apr-21** | **17-Apr-21** | **24-Apr-21** | **1-May-21** | **8-May-21** | **15-May-21** | **22-May-21** |
| --- | --- | --- | --- | --- | --- | --- | --- | --- | --- | --- | --- | --- | --- | --- |
| **10~14** | AZ | 1st | 0 | 0 | 0 | 0 | 0 | 1 | 0 | 0 | 0 | 2 | 0 | 0 |
|  |  | 2nd | 0 | 0 | 0 | 0 | 0 | 0 | 0 | 0 | 0 | 0 | 0 | 0 |
|  |  | 3rd | 0 | 0 | 0 | 0 | 0 | 0 | 0 | 0 | 0 | 0 | 0 | 0 |
|  | P | 1st | 17 | 3 | 10 | 19 | 21 | 66 | 94 | 120 | 97 | 48 | 401 | 517 |
|  |  | 2nd | 4 | 0 | 0 | 1 | 3 | 5 | 19 | 18 | 60 | 89 | 112 | 107 |
|  |  | 3rd | 0 | 0 | 0 | 0 | 0 | 0 | 0 | 0 | 0 | 0 | 0 | 0 |
|  | J | 1st | 0 | 0 | 1 | 0 | 0 | 0 | 1 | 0 | 0 | 0 | 0 | 0 |
|  |  | 3rd | 0 | 0 | 0 | 0 | 0 | 0 | 0 | 0 | 0 | 0 | 0 | 0 |
|  | M | 1st | 1 | 0 | 0 | 1 | 0 | 1 | 0 | 1 | 1 | 0 | 0 | 3 |
|  |  | 2nd | 0 | 0 | 0 | 0 | 1 | 0 | 0 | 1 | 0 | 1 | 0 | 1 |
|  |  | 3rd | 0 | 0 | 0 | 0 | 0 | 0 | 0 | 0 | 0 | 0 | 0 | 0 |
| **15~19** | AZ | 1st | 90 | 118 | 25 | 39 | 26 | 25 | 3 | 3 | 12 | 18 | 9 | 13 |
|  |  | 2nd | 0 | 0 | 0 | 0 | 0 | 0 | 1 | 8 | 4 | 6 | 2 | 62 |
|  |  | 3rd | 0 | 0 | 0 | 0 | 0 | 0 | 0 | 0 | 0 | 0 | 0 | 0 |
|  | P | 1st | 47 | 16 | 50 | 62 | 116 | 191 | 233 | 265 | 174 | 132 | 144 | 272 |
|  |  | 2nd | 16 | 3 | 3 | 9 | 17 | 43 | 65 | 108 | 172 | 235 | 248 | 185 |
|  |  | 3rd | 0 | 0 | 0 | 0 | 0 | 0 | 0 | 0 | 0 | 0 | 0 | 0 |
|  | J | 1st | 0 | 3 | 6 | 3 | 8 | 38 | 12 | 0 | 3 | 9 | 12 | 6 |
|  |  | 3rd | 0 | 0 | 0 | 0 | 0 | 0 | 0 | 0 | 0 | 0 | 0 | 0 |
|  | M | 1st | 19 | 3 | 14 | 23 | 42 | 49 | 47 | 50 | 33 | 22 | 21 | 32 |
|  |  | 2nd | 8 | 1 | 0 | 1 | 6 | 3 | 12 | 21 | 36 | 52 | 51 | 48 |
|  |  | 3rd | 0 | 0 | 0 | 0 | 0 | 0 | 0 | 0 | 0 | 0 | 0 | 0 |
| **20~29** | AZ | 1st | 43295 | 62665 | 12259 | 8356 | 6572 | 2148 | 44 | 81 | 110 | 200 | 235 | 223 |
|  |  | 2nd | 4 | 1 | 4 | 6 | 4 | 5 | 127 | 62 | 73 | 105 | 500 | 30795 |
|  |  | 3rd | 0 | 0 | 0 | 0 | 0 | 0 | 0 | 0 | 0 | 0 | 0 | 0 |
|  | P | 1st | 3102 | 6634 | 12004 | 721 | 1389 | 2404 | 2879 | 2566 | 2714 | 1490 | 1896 | 2205 |
|  |  | 2nd | 764 | 84 | 252 | 1779 | 6557 | 11931 | 656 | 1329 | 2235 | 2843 | 2497 | 2503 |
|  |  | 3rd | 0 | 0 | 0 | 0 | 0 | 0 | 0 | 0 | 0 | 0 | 0 | 0 |
|  | J | 1st | 15 | 165 | 473 | 72 | 167 | 1062 | 295 | 7 | 566 | 489 | 309 | 158 |
|  |  | 3rd | 0 | 0 | 0 | 0 | 0 | 0 | 0 | 0 | 0 | 0 | 0 | 0 |
|  | M | 1st | 1628 | 237 | 288 | 403 | 467 | 552 | 613 | 492 | 274 | 320 | 345 | 344 |
|  |  | 2nd | 1182 | 94 | 69 | 66 | 125 | 276 | 265 | 387 | 468 | 482 | 629 | 440 |
|  |  | 3rd | 0 | 0 | 0 | 0 | 0 | 0 | 0 | 0 | 0 | 0 | 0 | 0 |
| **30~39** | AZ | 1st | 44306 | 58021 | 12661 | 7586 | 4937 | 1727 | 12180 | 24582 | 107518 | 47403 | 5651 | 2660 |
|  |  | 2nd | 0 | 0 | 1 | 0 | 3 | 12 | 58 | 50 | 71 | 115 | 615 | 32971 |
|  |  | 3rd | 0 | 0 | 0 | 0 | 0 | 0 | 0 | 0 | 0 | 0 | 0 | 0 |
|  | P | 1st | 2972 | 6646 | 10183 | 770 | 1398 | 2113 | 2686 | 2526 | 2298 | 1345 | 1296 | 1793 |
|  |  | 2nd | 760 | 106 | 272 | 1669 | 6594 | 10104 | 698 | 1340 | 2008 | 2661 | 2384 | 2155 |
|  |  | 3rd | 0 | 0 | 0 | 0 | 0 | 1 | 0 | 0 | 1 | 0 | 1 | 0 |
|  | J | 1st | 17 | 121 | 533 | 75 | 169 | 876 | 343 | 12 | 140 | 271 | 213 | 145 |
|  |  | 3rd | 0 | 0 | 0 | 0 | 0 | 0 | 0 | 0 | 0 | 0 | 0 | 0 |
|  | M | 1st | 1240 | 195 | 278 | 371 | 455 | 473 | 449 | 440 | 209 | 326 | 414 | 289 |
|  |  | 2nd | 860 | 73 | 47 | 48 | 116 | 240 | 271 | 338 | 440 | 455 | 435 | 410 |
|  |  | 3rd | 0 | 0 | 0 | 0 | 0 | 0 | 0 | 0 | 0 | 0 | 0 | 0 |
| **40~49** | AZ | 1st | 63957 | 53590 | 10871 | 7468 | 5050 | 2227 | 17016 | 47640 | 157068 | 56326 | 6094 | 3242 |
|  |  | 2nd | 0 | 1 | 3 | 3 | 6 | 13 | 44 | 24 | 81 | 138 | 1087 | 47716 |
|  |  | 3rd | 0 | 0 | 0 | 0 | 0 | 0 | 0 | 0 | 0 | 0 | 0 | 0 |
|  | P | 1st | 1945 | 5022 | 7371 | 686 | 1491 | 2832 | 3764 | 3459 | 3034 | 1190 | 1275 | 1578 |
|  |  | 2nd | 431 | 86 | 180 | 1139 | 4969 | 7301 | 642 | 1474 | 2744 | 3668 | 3332 | 2777 |
|  |  | 3rd | 0 | 0 | 0 | 0 | 0 | 0 | 0 | 0 | 0 | 0 | 1 | 0 |
|  | J | 1st | 14 | 159 | 687 | 107 | 183 | 1254 | 679 | 7 | 197 | 513 | 342 | 228 |
|  |  | 3rd | 0 | 0 | 0 | 0 | 0 | 0 | 0 | 0 | 0 | 0 | 0 | 0 |
|  | M | 1st | 1233 | 223 | 208 | 352 | 408 | 417 | 439 | 401 | 206 | 287 | 316 | 259 |
|  |  | 2nd | 884 | 85 | 61 | 43 | 106 | 237 | 203 | 336 | 405 | 410 | 416 | 350 |
|  |  | 3rd | 0 | 0 | 0 | 0 | 0 | 0 | 0 | 0 | 0 | 0 | 0 | 0 |
| **50~59** | AZ | 1st | 105169 | 56178 | 14419 | 9973 | 7388 | 3576 | 19120 | 97958 | 159242 | 51150 | 5334 | 4522 |
|  |  | 2nd | 0 | 1 | 1 | 0 | 7 | 6 | 18 | 27 | 88 | 201 | 2272 | 73903 |
|  |  | 3rd | 0 | 0 | 0 | 0 | 0 | 0 | 0 | 0 | 0 | 0 | 0 | 0 |
|  | P | 1st | 1720 | 4217 | 4711 | 616 | 2341 | 4825 | 6491 | 5784 | 5253 | 1716 | 1232 | 1347 |
|  |  | 2nd | 407 | 69 | 159 | 994 | 4176 | 4688 | 571 | 2244 | 4773 | 6379 | 5641 | 4836 |
|  |  | 3rd | 0 | 0 | 0 | 0 | 0 | 0 | 0 | 0 | 0 | 0 | 0 | 0 |
|  | J | 1st | 26 | 205 | 746 | 116 | 194 | 1890 | 766 | 11 | 195 | 691 | 536 | 301 |
|  |  | 3rd | 0 | 0 | 0 | 0 | 0 | 0 | 0 | 0 | 0 | 0 | 0 | 0 |
|  | M | 1st | 1346 | 272 | 273 | 364 | 410 | 368 | 340 | 308 | 177 | 189 | 187 | 118 |
|  |  | 2nd | 910 | 107 | 64 | 70 | 131 | 294 | 267 | 360 | 399 | 345 | 312 | 266 |
|  |  | 3rd | 0 | 0 | 0 | 0 | 0 | 0 | 0 | 0 | 0 | 0 | 0 | 0 |
| **60~64** | AZ | 1st | 56964 | 20690 | 6005 | 4416 | 3784 | 2300 | 5693 | 66919 | 46963 | 16980 | 1968 | 1967 |
|  |  | 2nd | 0 | 0 | 0 | 1 | 5 | 2 | 6 | 11 | 20 | 52 | 1347 | 38629 |
|  |  | 3rd | 0 | 0 | 0 | 0 | 0 | 0 | 0 | 0 | 0 | 0 | 0 | 0 |
|  | P | 1st | 534 | 801 | 928 | 224 | 996 | 2047 | 2901 | 2881 | 2342 | 563 | 352 | 492 |
|  |  | 2nd | 173 | 34 | 60 | 226 | 768 | 929 | 205 | 971 | 1988 | 2778 | 2756 | 2125 |
|  |  | 3rd | 0 | 0 | 0 | 0 | 0 | 0 | 0 | 0 | 0 | 0 | 0 | 0 |
|  | J | 1st | 12 | 118 | 432 | 67 | 116 | 1179 | 457 | 9 | 108 | 432 | 335 | 141 |
|  |  | 3rd | 0 | 0 | 0 | 0 | 0 | 0 | 0 | 0 | 0 | 0 | 0 | 0 |
|  | M | 1st | 703 | 140 | 166 | 179 | 120 | 118 | 92 | 99 | 89 | 66 | 50 | 59 |
|  |  | 2nd | 446 | 81 | 37 | 41 | 75 | 151 | 149 | 179 | 127 | 105 | 90 | 87 |
|  |  | 3rd | 0 | 0 | 0 | 0 | 0 | 0 | 0 | 0 | 0 | 0 | 0 | 0 |
| **65~69** | AZ | 1st | 319 | 174 | 357 | 20515 | 17936 | 8548 | 6965 | 46596 | 28802 | 11619 | 1357 | 1631 |
|  |  | 2nd | 0 | 0 | 1 | 2 | 1 | 1 | 5 | 9 | 15 | 15 | 46 | 1472 |
|  |  | 3rd | 0 | 0 | 0 | 0 | 0 | 0 | 0 | 0 | 0 | 0 | 0 | 0 |
|  | P | 1st | 713 | 473 | 465 | 130 | 583 | 1414 | 1947 | 1916 | 1519 | 332 | 255 | 332 |
|  |  | 2nd | 332 | 72 | 82 | 184 | 451 | 474 | 124 | 560 | 1327 | 1834 | 1783 | 1362 |
|  |  | 3rd | 0 | 0 | 0 | 0 | 0 | 0 | 0 | 0 | 0 | 0 | 0 | 0 |
|  | J | 1st | 9 | 76 | 239 | 382 | 34 | 528 | 171 | 4 | 56 | 222 | 154 | 68 |
|  |  | 3rd | 0 | 0 | 0 | 0 | 0 | 0 | 0 | 0 | 0 | 0 | 0 | 0 |
|  | M | 1st | 891 | 101 | 67 | 81 | 53 | 64 | 54 | 69 | 60 | 43 | 45 | 33 |
|  |  | 2nd | 507 | 106 | 87 | 55 | 104 | 102 | 77 | 82 | 53 | 48 | 39 | 42 |
|  |  | 3rd | 0 | 0 | 0 | 0 | 0 | 0 | 0 | 0 | 0 | 0 | 0 | 0 |
| **70~74** | AZ | 1st | 34 | 24 | 49 | 10807 | 10012 | 4506 | 3116 | 17723 | 13695 | 6211 | 559 | 861 |
|  |  | 2nd | 0 | 0 | 1 | 3 | 1 | 1 | 6 | 6 | 9 | 6 | 10 | 581 |
|  |  | 3rd | 0 | 0 | 0 | 0 | 0 | 0 | 0 | 0 | 0 | 0 | 0 | 0 |
|  | P | 1st | 360 | 172 | 136 | 49 | 616 | 1419 | 2115 | 1713 | 1509 | 212 | 115 | 168 |
|  |  | 2nd | 182 | 46 | 51 | 70 | 162 | 139 | 39 | 530 | 1328 | 2037 | 1696 | 1372 |
|  |  | 3rd | 0 | 0 | 0 | 0 | 0 | 0 | 0 | 0 | 0 | 0 | 0 | 0 |
|  | J | 1st | 3 | 22 | 86 | 113 | 10 | 130 | 62 | 1 | 17 | 77 | 58 | 19 |
|  |  | 3rd | 0 | 0 | 0 | 0 | 0 | 0 | 0 | 0 | 0 | 0 | 0 | 0 |
|  | M | 1st | 469 | 53 | 41 | 40 | 28 | 47 | 27 | 36 | 18 | 22 | 20 | 13 |
|  |  | 2nd | 265 | 60 | 39 | 33 | 59 | 49 | 42 | 41 | 22 | 19 | 18 | 30 |
|  |  | 3rd | 0 | 0 | 0 | 0 | 0 | 0 | 0 | 0 | 0 | 0 | 0 | 0 |
| **75~79** | AZ | 1st | 7 | 2 | 17 | 9283 | 10985 | 5212 | 2851 | 1923 | 1352 | 401 | 150 | 523 |
|  |  | 2nd | 0 | 0 | 0 | 0 | 1 | 1 | 1 | 1 | 4 | 0 | 3 | 344 |
|  |  | 3rd | 0 | 0 | 0 | 0 | 0 | 0 | 0 | 0 | 0 | 0 | 0 | 0 |
|  | P | 1st | 280 | 53 | 37 | 54 | 12874 | 37948 | 85362 | 154161 | 257442 | 37968 | 11076 | 16769 |
|  |  | 2nd | 163 | 33 | 25 | 44 | 52 | 36 | 26 | 12523 | 37361 | 83719 | 151775 | 246249 |
|  |  | 3rd | 0 | 0 | 0 | 0 | 0 | 0 | 0 | 0 | 0 | 0 | 0 | 0 |
|  | J | 1st | 0 | 3 | 20 | 38 | 5 | 60 | 17 | 2 | 2 | 21 | 21 | 8 |
|  |  | 3rd | 0 | 0 | 0 | 0 | 0 | 0 | 0 | 0 | 0 | 0 | 0 | 0 |
|  | M | 1st | 314 | 31 | 18 | 11 | 18 | 5 | 10 | 13 | 6 | 13 | 5 | 9 |
|  |  | 2nd | 167 | 28 | 60 | 17 | 28 | 35 | 18 | 10 | 18 | 2 | 9 | 10 |
|  |  | 3rd | 0 | 0 | 0 | 0 | 0 | 0 | 0 | 0 | 0 | 0 | 0 | 0 |
| **≥80** | AZ | 1st | 4 | 2 | 4 | 38458 | 56119 | 30204 | 17761 | 10101 | 5780 | 1476 | 704 | 2298 |
|  |  | 2nd | 0 | 0 | 0 | 0 | 1 | 0 | 0 | 1 | 5 | 1 | 8 | 1265 |
|  |  | 3rd | 0 | 0 | 0 | 0 | 0 | 0 | 0 | 0 | 0 | 0 | 0 | 0 |
|  | P | 1st | 220 | 31 | 45 | 34 | 27870 | 84399 | 169765 | 266894 | 346467 | 53863 | 14775 | 22922 |
|  |  | 2nd | 127 | 30 | 30 | 22 | 20 | 27 | 14 | 26877 | 82544 | 164357 | 261386 | 325825 |
|  |  | 3rd | 0 | 0 | 0 | 0 | 0 | 0 | 0 | 0 | 0 | 0 | 0 | 0 |
|  | J | 1st | 2 | 6 | 5 | 5 | 3 | 25 | 11 | 0 | 0 | 9 | 2 | 2 |
|  |  | 3rd | 0 | 0 | 0 | 0 | 0 | 0 | 0 | 0 | 0 | 0 | 0 | 0 |
|  | M | 1st | 255 | 19 | 14 | 6 | 15 | 4 | 11 | 1 | 4 | 11 | 5 | 4 |
|  |  | 2nd | 151 | 24 | 44 | 11 | 13 | 17 | 13 | 4 | 12 | 5 | 11 | 1 |
|  |  | 3rd | 0 | 0 | 0 | 0 | 0 | 0 | 0 | 0 | 0 | 0 | 0 | 0 |

| **Age** | **Vaccine** | **Dose** | **29-May-21** | **5-Jun-21** | **12-Jun-21** | **19-Jun-21** | **26-Jun-21** | **3-Jul-21** | **10-Jul-21** | **17-Jul-21** | **24-Jul-21** | **31-Jul-21** | **7-Aug-21** | **14-Aug-21** | **21-Aug-21** |
| --- | --- | --- | --- | --- | --- | --- | --- | --- | --- | --- | --- | --- | --- | --- | --- |
| **10~14** | AZ | 1st | 1 | 0 | 0 | 0 | 1 | 0 | 0 | 0 | 0 | 0 | 1 | 2 | 0 |
|  |  | 2nd | 0 | 0 | 2 | 0 | 0 | 0 | 0 | 0 | 0 | 0 | 0 | 0 | 0 |
|  |  | 3rd | 0 | 0 | 0 | 0 | 0 | 0 | 0 | 0 | 0 | 0 | 0 | 0 | 0 |
|  | P | 1st | 334 | 435 | 323 | 345 | 219 | 240 | 377 | 257 | 10784 | 2565 | 483 | 1912 | 210 |
|  |  | 2nd | 37 | 361 | 386 | 196 | 115 | 153 | 254 | 297 | 343 | 378 | 389 | 10526 | 2450 |
|  |  | 3rd | 0 | 0 | 0 | 0 | 0 | 0 | 0 | 0 | 0 | 0 | 0 | 0 | 0 |
|  | J | 1st | 0 | 0 | 0 | 0 | 0 | 0 | 0 | 1 | 1 | 0 | 0 | 0 | 1 |
|  |  | 3rd | 0 | 0 | 0 | 0 | 0 | 0 | 0 | 0 | 0 | 0 | 0 | 0 | 0 |
|  | M | 1st | 1 | 0 | 0 | 0 | 2 | 0 | 2 | 0 | 1 | 0 | 1 | 4 | 7 |
|  |  | 2nd | 1 | 1 | 1 | 0 | 0 | 1 | 1 | 0 | 1 | 0 | 1 | 3 | 0 |
|  |  | 3rd | 0 | 0 | 0 | 0 | 0 | 0 | 0 | 0 | 0 | 0 | 0 | 0 | 0 |
| **15~19** | AZ | 1st | 16 | 22 | 10 | 17 | 18 | 14 | 11 | 8 | 14 | 23 | 31 | 43 | 15 |
|  |  | 2nd | 115 | 44 | 29 | 22 | 15 | 15 | 9 | 7 | 12 | 17 | 15 | 9 | 7 |
|  |  | 3rd | 0 | 0 | 0 | 0 | 0 | 0 | 0 | 0 | 0 | 0 | 0 | 1 | 0 |
|  | P | 1st | 225 | 399 | 2792 | 2676 | 2634 | 295 | 1472 | 3214 | 366758 | 77033 | 13787 | 66772 | 18405 |
|  |  | 2nd | 98 | 58 | 96 | 110 | 239 | 2383 | 2621 | 3028 | 546 | 1485 | 3150 | 357786 | 67961 |
|  |  | 3rd | 0 | 0 | 0 | 0 | 0 | 0 | 0 | 0 | 0 | 0 | 0 | 1 | 2 |
|  | J | 1st | 8 | 1 | 7 | 11 | 8 | 8 | 7 | 7 | 9 | 10 | 16 | 8 | 8 |
|  |  | 3rd | 0 | 0 | 0 | 0 | 0 | 0 | 0 | 0 | 0 | 0 | 0 | 0 | 0 |
|  | M | 1st | 35 | 28 | 21 | 14 | 103 | 216 | 321 | 48 | 33 | 4140 | 501 | 747 | 6272 |
|  |  | 2nd | 35 | 16 | 13 | 12 | 17 | 20 | 18 | 26 | 90 | 212 | 328 | 67 | 49 |
|  |  | 3rd | 0 | 0 | 0 | 0 | 0 | 0 | 0 | 0 | 0 | 0 | 0 | 1 | 0 |
| **20~29** | AZ | 1st | 198 | 223 | 197 | 206 | 192 | 129 | 112 | 107 | 111 | 172 | 178 | 311 | 127 |
|  |  | 2nd | 54659 | 23561 | 10086 | 7299 | 3570 | 640 | 170 | 85 | 130 | 114 | 156 | 89 | 104 |
|  |  | 3rd | 0 | 0 | 0 | 0 | 0 | 0 | 0 | 0 | 0 | 0 | 2 | 5 | 14 |
|  | P | 1st | 2728 | 3875 | 160836 | 234900 | 196246 | 4591 | 68789 | 62303 | 75321 | 212919 | 201605 | 183004 | 219701 |
|  |  | 2nd | 1264 | 969 | 1563 | 2497 | 3335 | 152080 | 228605 | 200376 | 12485 | 66712 | 61697 | 73899 | 10236 |
|  |  | 3rd | 0 | 2 | 0 | 0 | 1 | 0 | 3 | 2 | 0 | 3 | 3 | 5 | 11 |
|  | J | 1st | 182 | 135 | 188 | 358 | 232 | 213 | 221 | 176 | 192 | 278 | 180 | 119 | 142 |
|  |  | 3rd | 0 | 0 | 0 | 0 | 0 | 0 | 0 | 0 | 0 | 0 | 0 | 0 | 0 |
|  | M | 1st | 396 | 394 | 349 | 604 | 16224 | 19027 | 20762 | 1334 | 486 | 59388 | 6739 | 6900 | 55837 |
|  |  | 2nd | 274 | 271 | 202 | 200 | 385 | 453 | 471 | 642 | 13610 | 20706 | 20908 | 1614 | 626 |
|  |  | 3rd | 0 | 0 | 0 | 0 | 0 | 0 | 0 | 0 | 0 | 2 | 1 | 1 | 3 |
| **30~39** | AZ | 1st | 27224 | 42018 | 62557 | 73159 | 2002 | 681 | 137 | 175 | 154 | 269 | 321 | 1221 | 17770 |
|  |  | 2nd | 52124 | 22699 | 9446 | 6777 | 4279 | 8458 | 214 | 189 | 168 | 174 | 148 | 230 | 471 |
|  |  | 3rd | 0 | 0 | 0 | 0 | 0 | 0 | 0 | 0 | 0 | 3 | 0 | 4 | 8 |
|  | P | 1st | 1911 | 1683 | 1861 | 2146 | 2210 | 2341 | 9868 | 97957 | 90859 | 230676 | 187753 | 130621 | 173122 |
|  |  | 2nd | 1096 | 756 | 949 | 1608 | 1406 | 3100 | 23177 | 89728 | 69409 | 20583 | 96196 | 114986 | 53206 |
|  |  | 3rd | 0 | 0 | 2 | 2 | 1 | 0 | 2 | 4 | 5 | 4 | 3 | 17 | 23 |
|  | J | 1st | 157 | 141 | 436678 | 364587 | 2676 | 1387 | 136 | 116 | 144 | 144 | 122 | 70 | 88 |
|  |  | 3rd | 0 | 0 | 0 | 0 | 0 | 0 | 0 | 0 | 0 | 0 | 0 | 1 | 2 |
|  | M | 1st | 345 | 327 | 283 | 220 | 1948 | 2207 | 2110 | 541 | 308 | 38053 | 4160 | 5074 | 48267 |
|  |  | 2nd | 195 | 270 | 224 | 199 | 342 | 392 | 404 | 301 | 1773 | 2439 | 2060 | 631 | 368 |
|  |  | 3rd | 0 | 0 | 0 | 0 | 0 | 0 | 1 | 0 | 1 | 2 | 2 | 7 | 9 |
| **40~49** | AZ | 1st | 66583 | 136870 | 158551 | 90061 | 2646 | 957 | 213 | 154 | 175 | 302 | 378 | 2692 | 40016 |
|  |  | 2nd | 56089 | 19428 | 9046 | 6716 | 5120 | 11770 | 354 | 221 | 288 | 240 | 205 | 337 | 989 |
|  |  | 3rd | 0 | 0 | 0 | 0 | 0 | 0 | 0 | 0 | 0 | 0 | 0 | 4 | 2 |
|  | P | 1st | 1969 | 1822 | 2045 | 2033 | 1993 | 1829 | 9171 | 178765 | 121444 | 345856 | 255823 | 169115 | 250494 |
|  |  | 2nd | 1103 | 744 | 992 | 1802 | 1408 | 4537 | 45043 | 140191 | 78674 | 20924 | 178792 | 189266 | 148998 |
|  |  | 3rd | 1 | 1 | 0 | 2 | 0 | 0 | 3 | 4 | 2 | 1 | 9 | 15 | 23 |
|  | J | 1st | 231 | 174 | 93624 | 101265 | 3789 | 2218 | 151 | 113 | 112 | 142 | 84 | 74 | 60 |
|  |  | 3rd | 0 | 0 | 0 | 0 | 0 | 0 | 0 | 0 | 0 | 0 | 0 | 0 | 0 |
|  | M | 1st | 244 | 233 | 188 | 125 | 677 | 737 | 823 | 289 | 211 | 52568 | 5511 | 7107 | 79499 |
|  |  | 2nd | 170 | 247 | 167 | 151 | 250 | 389 | 383 | 228 | 620 | 813 | 831 | 318 | 258 |
|  |  | 3rd | 0 | 0 | 0 | 0 | 0 | 0 | 0 | 0 | 0 | 2 | 0 | 5 | 6 |
| **50~59** | AZ | 1st | 74548 | 142164 | 148643 | 46322 | 2906 | 1188 | 598 | 339 | 233 | 228 | 4638 | 27584 | 11383 |
|  |  | 2nd | 72423 | 21688 | 11764 | 8774 | 6630 | 14438 | 16238 | 19712 | 12506 | 6344 | 4934 | 85069 | 140198 |
|  |  | 3rd | 0 | 0 | 0 | 0 | 0 | 0 | 0 | 0 | 0 | 1 | 0 | 1 | 3 |
|  | P | 1st | 2370 | 1673 | 2072 | 1843 | 1926 | 1843 | 7310 | 121038 | 88863 | 814399 | 663463 | 608234 | 1314376 |
|  |  | 2nd | 1480 | 882 | 1180 | 2539 | 1769 | 4718 | 76760 | 127020 | 56253 | 12265 | 117526 | 87696 | 14995 |
|  |  | 3rd | 0 | 1 | 0 | 1 | 0 | 0 | 0 | 6 | 1 | 9 | 12 | 27 | 36 |
|  | J | 1st | 266 | 178 | 22880 | 35153 | 2824 | 2202 | 87 | 105 | 99 | 101 | 101 | 44 | 49 |
|  |  | 3rd | 0 | 0 | 0 | 0 | 0 | 0 | 0 | 0 | 1 | 0 | 0 | 0 | 1 |
|  | M | 1st | 85 | 172 | 113 | 67 | 435 | 480 | 475 | 177 | 166 | 580158 | 32259 | 25101 | 1134734 |
|  |  | 2nd | 166 | 130 | 119 | 97 | 240 | 313 | 227 | 160 | 386 | 502 | 481 | 226 | 206 |
|  |  | 3rd | 0 | 0 | 0 | 0 | 0 | 0 | 0 | 0 | 1 | 2 | 0 | 4 | 4 |
| **60~64** | AZ | 1st | 4920 | 6114 | 1765619 | 1273341 | 1233 | 471 | 341 | 201 | 140 | 96 | 14106 | 104758 | 52791 |
|  |  | 2nd | 32678 | 8226 | 5343 | 4174 | 3171 | 4644 | 5930 | 10327 | 6669 | 3271 | 3120 | 31912 | 57988 |
|  |  | 3rd | 0 | 0 | 2 | 0 | 0 | 0 | 0 | 0 | 0 | 0 | 0 | 0 | 2 |
|  | P | 1st | 927 | 648 | 719 | 437 | 387 | 414 | 63326 | 42593 | 6374 | 40692 | 26872 | 15240 | 8009 |
|  |  | 2nd | 549 | 339 | 500 | 1057 | 712 | 885 | 56829 | 36208 | 14672 | 61719 | 41652 | 7578 | 1051 |
|  |  | 3rd | 0 | 0 | 0 | 0 | 0 | 0 | 0 | 3 | 0 | 2 | 7 | 7 | 16 |
|  | J | 1st | 114 | 68 | 10723 | 31690 | 423 | 131 | 24 | 17 | 18 | 21 | 24 | 13 | 17 |
|  |  | 3rd | 0 | 0 | 0 | 0 | 0 | 0 | 0 | 0 | 0 | 0 | 0 | 0 | 0 |
|  | M | 1st | 46 | 88 | 66 | 45 | 72 | 62 | 102 | 54 | 31 | 29800 | 1279 | 898 | 7218 |
|  |  | 2nd | 59 | 54 | 46 | 55 | 119 | 166 | 103 | 81 | 87 | 70 | 115 | 67 | 47 |
|  |  | 3rd | 0 | 0 | 0 | 0 | 0 | 0 | 0 | 1 | 0 | 1 | 3 | 0 | 3 |
| **65~69** | AZ | 1st | 457742 | 808832 | 572295 | 588477 | 908 | 345 | 225 | 107 | 110 | 56 | 6447 | 51065 | 26292 |
|  |  | 2nd | 2023 | 2195 | 14497 | 16619 | 10099 | 6354 | 5507 | 8306 | 4962 | 2491 | 3344 | 469480 | 793680 |
|  |  | 3rd | 0 | 1 | 0 | 1 | 0 | 0 | 0 | 0 | 0 | 0 | 0 | 0 | 0 |
|  | P | 1st | 732 | 462 | 537 | 327 | 275 | 243 | 29829 | 15077 | 2780 | 17776 | 15519 | 9596 | 4580 |
|  |  | 2nd | 382 | 247 | 377 | 841 | 518 | 651 | 38887 | 20361 | 9367 | 28979 | 14638 | 3390 | 683 |
|  |  | 3rd | 0 | 0 | 0 | 0 | 1 | 0 | 1 | 1 | 1 | 1 | 4 | 1 | 6 |
|  | J | 1st | 91 | 39 | 4286 | 13215 | 207 | 53 | 6 | 6 | 15 | 13 | 13 | 10 | 6 |
|  |  | 3rd | 0 | 0 | 0 | 0 | 0 | 0 | 0 | 0 | 0 | 0 | 0 | 0 | 0 |
|  | M | 1st | 45 | 52 | 48 | 26 | 47 | 47 | 44 | 50 | 20 | 14511 | 778 | 484 | 4195 |
|  |  | 2nd | 48 | 36 | 40 | 39 | 99 | 108 | 61 | 41 | 47 | 55 | 57 | 69 | 52 |
|  |  | 3rd | 0 | 0 | 0 | 0 | 0 | 0 | 0 | 1 | 0 | 0 | 0 | 1 | 6 |
| **70~74** | AZ | 1st | 575417 | 542063 | 313248 | 318052 | 714 | 273 | 184 | 104 | 77 | 37 | 3711 | 28830 | 14460 |
|  |  | 2nd | 877 | 1130 | 7499 | 9089 | 5218 | 2934 | 2500 | 4045 | 2250 | 1058 | 2539 | 575773 | 534347 |
|  |  | 3rd | 0 | 0 | 0 | 0 | 0 | 0 | 0 | 0 | 0 | 0 | 0 | 0 | 5 |
|  | P | 1st | 534 | 336 | 435 | 159 | 145 | 118 | 16576 | 8433 | 1682 | 11511 | 10430 | 6643 | 3154 |
|  |  | 2nd | 317 | 160 | 224 | 602 | 349 | 454 | 14578 | 9275 | 5136 | 16059 | 8207 | 2022 | 420 |
|  |  | 3rd | 0 | 0 | 1 | 0 | 1 | 1 | 0 | 1 | 0 | 1 | 4 | 2 | 4 |
|  | J | 1st | 25 | 8 | 2162 | 5845 | 108 | 28 | 0 | 4 | 3 | 2 | 5 | 0 | 0 |
|  |  | 3rd | 0 | 0 | 0 | 0 | 0 | 0 | 0 | 0 | 0 | 0 | 0 | 0 | 0 |
|  | M | 1st | 27 | 28 | 21 | 18 | 22 | 20 | 29 | 11 | 5 | 9220 | 498 | 344 | 2654 |
|  |  | 2nd | 17 | 27 | 27 | 20 | 52 | 46 | 35 | 25 | 17 | 25 | 30 | 33 | 25 |
|  |  | 3rd | 0 | 0 | 0 | 0 | 0 | 0 | 0 | 0 | 0 | 0 | 0 | 0 | 4 |
| **75~79** | AZ | 1st | 784 | 638 | 1722 | 2903 | 797 | 257 | 135 | 65 | 74 | 43 | 444 | 1564 | 1112 |
|  |  | 2nd | 703 | 1059 | 6671 | 9222 | 5841 | 2536 | 1857 | 952 | 438 | 232 | 459 | 1270 | 1023 |
|  |  | 3rd | 0 | 0 | 0 | 0 | 0 | 0 | 0 | 0 | 0 | 0 | 0 | 0 | 1 |
|  | P | 1st | 193557 | 313029 | 279804 | 8767 | 8002 | 7489 | 5087 | 7047 | 2754 | 2644 | 4373 | 2959 | 3322 |
|  |  | 2nd | 48092 | 11913 | 17448 | 190439 | 308477 | 276342 | 13252 | 9711 | 8064 | 5528 | 7140 | 2950 | 516 |
|  |  | 3rd | 0 | 0 | 0 | 0 | 0 | 0 | 1 | 0 | 0 | 0 | 0 | 2 | 4 |
|  | J | 1st | 5 | 4 | 26 | 109 | 40 | 9 | 0 | 0 | 0 | 1 | 1 | 0 | 0 |
|  |  | 3rd | 0 | 0 | 0 | 0 | 0 | 0 | 0 | 0 | 0 | 0 | 0 | 0 | 0 |
|  | M | 1st | 7 | 6 | 3 | 5 | 2 | 7 | 2 | 2 | 2 | 112 | 19 | 23 | 88 |
|  |  | 2nd | 7 | 12 | 7 | 9 | 20 | 14 | 6 | 6 | 3 | 4 | 4 | 3 | 4 |
|  |  | 3rd | 0 | 0 | 0 | 0 | 0 | 0 | 0 | 0 | 0 | 0 | 0 | 0 | 1 |
| **≥80** | AZ | 1st | 3240 | 1994 | 5393 | 8351 | 4298 | 1502 | 916 | 414 | 331 | 217 | 2282 | 7255 | 5369 |
|  |  | 2nd | 2663 | 4541 | 28079 | 45802 | 32950 | 15473 | 10755 | 5335 | 2344 | 1102 | 2149 | 5094 | 3965 |
|  |  | 3rd | 0 | 0 | 0 | 0 | 0 | 0 | 0 | 0 | 0 | 0 | 0 | 1 | 0 |
|  | P | 1st | 199724 | 197667 | 180711 | 11913 | 11807 | 10680 | 7133 | 10156 | 4417 | 4205 | 6513 | 4713 | 4998 |
|  |  | 2nd | 72566 | 16996 | 24553 | 195695 | 194753 | 176724 | 14670 | 13090 | 10999 | 7629 | 10216 | 4684 | 676 |
|  |  | 3rd | 0 | 0 | 0 | 0 | 0 | 0 | 0 | 0 | 0 | 0 | 1 | 1 | 2 |
|  | J | 1st | 0 | 3 | 34 | 163 | 80 | 45 | 0 | 0 | 0 | 0 | 1 | 0 | 0 |
|  |  | 3rd | 0 | 0 | 0 | 0 | 0 | 0 | 0 | 0 | 0 | 0 | 0 | 0 | 0 |
|  | M | 1st | 2 | 2 | 1 | 0 | 1 | 2 | 0 | 0 | 1 | 131 | 15 | 54 | 130 |
|  |  | 2nd | 4 | 11 | 7 | 3 | 9 | 6 | 3 | 1 | 1 | 4 | 0 | 2 | 1 |
|  |  | 3rd | 0 | 0 | 0 | 0 | 0 | 0 | 0 | 0 | 0 | 0 | 0 | 0 | 3 |

| **Age** | **Vaccine** | **Dose** | **28-Aug-21** | **4-Sep-21** | **11-Sep-21** | **18-Sep-21** | **25-Sep-21** | **2-Oct-21** | **9-Oct-21** | **16-Oct-21** | **23-Oct-21** | **30-Oct-21** | **6-Nov-21** | **13-Nov-21** | **20-Nov-21** |
| --- | --- | --- | --- | --- | --- | --- | --- | --- | --- | --- | --- | --- | --- | --- | --- |
| **10~14** | AZ | 1st | 0 | 1 | 1 | 1 | 0 | 0 | 1 | 1 | 1 | 1 | 0 | 0 | 0 |
|  |  | 2nd | 0 | 0 | 0 | 1 | 0 | 1 | 2 | 2 | 0 | 1 | 0 | 0 | 0 |
|  |  | 3rd | 0 | 0 | 0 | 0 | 0 | 0 | 0 | 0 | 0 | 0 | 0 | 0 | 0 |
|  | P | 1st | 210 | 211 | 222 | 185 | 148 | 157 | 223 | 323 | 217855 | 195771 | 208038 | 305437 | 158156 |
|  |  | 2nd | 588 | 322 | 1887 | 342 | 200 | 199 | 160 | 148 | 210 | 249 | 349 | 186544 | 168835 |
|  |  | 3rd | 0 | 0 | 2 | 0 | 0 | 1 | 0 | 2 | 1 | 4 | 2 | 3 | 4 |
|  | J | 1st | 0 | 1 | 0 | 0 | 0 | 0 | 0 | 0 | 0 | 0 | 0 | 1 | 0 |
|  |  | 3rd | 0 | 0 | 0 | 0 | 0 | 0 | 0 | 0 | 0 | 0 | 0 | 0 | 0 |
|  | M | 1st | 3 | 2 | 2 | 3 | 12 | 4 | 2 | 0 | 15 | 8 | 2 | 2 | 1 |
|  |  | 2nd | 0 | 1 | 3 | 1 | 3 | 4 | 2 | 4 | 14 | 3 | 2 | 1 | 0 |
|  |  | 3rd | 0 | 0 | 0 | 0 | 0 | 0 | 0 | 0 | 0 | 0 | 0 | 0 | 0 |
| **15~19** | AZ | 1st | 15 | 5 | 32 | 52 | 70 | 42 | 26 | 7 | 35 | 23 | 9 | 10 | 8 |
|  |  | 2nd | 7 | 11 | 23 | 14 | 15 | 32 | 16 | 9 | 16 | 8 | 27 | 35 | 43 |
|  |  | 3rd | 0 | 0 | 0 | 0 | 0 | 1 | 0 | 0 | 0 | 0 | 0 | 0 | 0 |
|  | P | 1st | 44323 | 34299 | 63223 | 45439 | 18654 | 16098 | 2828 | 2762 | 2524 | 3765 | 3666 | 2699 | 2634 |
|  |  | 2nd | 6642 | 6832 | 61374 | 20940 | 14882 | 34399 | 65647 | 45299 | 36537 | 33567 | 8108 | 4480 | 4702 |
|  |  | 3rd | 1 | 5 | 11 | 9 | 5 | 6 | 3 | 11 | 10 | 11 | 49 | 57 | 87 |
|  | J | 1st | 9 | 6 | 1 | 1 | 2 | 2 | 1 | 2 | 0 | 1 | 1 | 1 | 1 |
|  |  | 3rd | 0 | 0 | 0 | 0 | 0 | 0 | 0 | 0 | 0 | 0 | 0 | 0 | 0 |
|  | M | 1st | 671 | 1262 | 8354 | 42382 | 23941 | 17904 | 2417 | 6019 | 670 | 1038 | 1078 | 827 | 404 |
|  |  | 2nd | 152 | 722 | 3407 | 1894 | 2170 | 3897 | 3106 | 14801 | 23520 | 37427 | 9641 | 7737 | 1171 |
|  |  | 3rd | 0 | 0 | 0 | 1 | 0 | 0 | 0 | 0 | 1 | 2 | 6 | 13 | 7 |
| **20~29** | AZ | 1st | 120 | 63 | 217 | 178 | 145 | 81 | 75 | 25 | 28 | 22 | 15 | 14 | 28 |
|  |  | 2nd | 108 | 91 | 186 | 154 | 127 | 161 | 151 | 113 | 135 | 109 | 92 | 124 | 124 |
|  |  | 3rd | 5 | 3 | 2 | 3 | 1 | 2 | 2 | 4 | 5 | 6 | 0 | 2 | 1 |
|  | P | 1st | 461985 | 415594 | 795770 | 564169 | 235523 | 240114 | 23275 | 32934 | 29600 | 40890 | 41773 | 28176 | 28962 |
|  |  | 2nd | 14302 | 102889 | 213965 | 262444 | 152330 | 386554 | 750693 | 573253 | 526712 | 414141 | 94793 | 50800 | 52412 |
|  |  | 3rd | 9 | 40 | 33 | 25 | 23 | 56 | 51 | 1634 | 6159 | 4911 | 2288 | 7955 | 16635 |
|  | J | 1st | 118 | 106 | 100 | 56 | 28 | 31 | 24 | 38 | 47 | 15 | 20 | 15 | 14 |
|  |  | 3rd | 0 | 0 | 1 | 0 | 0 | 0 | 0 | 0 | 1 | 6 | 2 | 5 | 21 |
|  | M | 1st | 6437 | 15801 | 90224 | 453550 | 265624 | 256397 | 36778 | 85390 | 11466 | 14379 | 14457 | 12206 | 5598 |
|  |  | 2nd | 1345 | 9572 | 50333 | 19665 | 17654 | 37674 | 34803 | 165555 | 290277 | 423122 | 112516 | 107485 | 15417 |
|  |  | 3rd | 3 | 1 | 4 | 7 | 6 | 7 | 7 | 15 | 19 | 114 | 305 | 736 | 790 |
| **30~39** | AZ | 1st | 23499 | 10863 | 2950 | 621 | 389 | 522 | 654 | 395 | 347 | 249 | 129 | 56 | 42 |
|  |  | 2nd | 1161 | 1245 | 494 | 389 | 308 | 580 | 672 | 714 | 787 | 645 | 378 | 312 | 272 |
|  |  | 3rd | 6 | 2 | 3 | 6 | 2 | 10 | 4 | 8 | 6 | 5 | 2 | 4 | 1 |
|  | P | 1st | 297474 | 279659 | 695458 | 468690 | 201872 | 216623 | 13171 | 23067 | 21993 | 27160 | 29358 | 21211 | 20005 |
|  |  | 2nd | 70948 | 207856 | 180441 | 201025 | 104738 | 283000 | 527661 | 521805 | 479688 | 352567 | 80061 | 40437 | 29290 |
|  |  | 3rd | 31 | 53 | 29 | 20 | 22 | 70 | 112 | 1710 | 5585 | 4436 | 10373 | 22788 | 34201 |
|  | J | 1st | 20891 | 36121 | 26300 | 20937 | 3769 | 8016 | 4647 | 3616 | 3000 | 2980 | 1487 | 1190 | 919 |
|  |  | 3rd | 2 | 0 | 1 | 3 | 4 | 2 | 5 | 2 | 4 | 12 | 168 | 1362 | 2047 |
|  | M | 1st | 5570 | 17560 | 94019 | 373949 | 231764 | 246074 | 29042 | 76601 | 10773 | 12017 | 12019 | 10769 | 8086 |
|  |  | 2nd | 626 | 5532 | 33027 | 13319 | 14482 | 34785 | 35400 | 149351 | 259008 | 365197 | 99608 | 93934 | 23952 |
|  |  | 3rd | 9 | 7 | 6 | 10 | 8 | 6 | 15 | 46 | 22 | 155 | 14365 | 192869 | 110098 |
| **40~49** | AZ | 1st | 57628 | 23989 | 5315 | 871 | 381 | 478 | 658 | 366 | 319 | 213 | 128 | 36 | 33 |
|  |  | 2nd | 2250 | 1551 | 688 | 554 | 527 | 1151 | 1322 | 1332 | 1178 | 789 | 389 | 335 | 296 |
|  |  | 3rd | 3 | 6 | 1 | 5 | 4 | 12 | 5 | 10 | 8 | 7 | 0 | 4 | 5 |
|  | P | 1st | 396098 | 370183 | 1095800 | 676284 | 253732 | 254323 | 11107 | 17280 | 15771 | 18576 | 18433 | 13789 | 12077 |
|  |  | 2nd | 160324 | 306146 | 235249 | 283146 | 174185 | 480551 | 772963 | 796108 | 657351 | 433483 | 75986 | 32922 | 21410 |
|  |  | 3rd | 48 | 49 | 44 | 41 | 36 | 65 | 85 | 1582 | 4409 | 3886 | 14059 | 24699 | 36075 |
|  | J | 1st | 21006 | 32523 | 24497 | 18765 | 3442 | 6442 | 3534 | 2692 | 2333 | 2213 | 1209 | 904 | 636 |
|  |  | 3rd | 0 | 0 | 2 | 7 | 1 | 2 | 2 | 1 | 3 | 3 | 95 | 929 | 1480 |
|  | M | 1st | 9417 | 27277 | 152878 | 574523 | 314081 | 301334 | 24166 | 67071 | 8069 | 8532 | 8006 | 7353 | 5342 |
|  |  | 2nd | 588 | 6954 | 46454 | 19908 | 25361 | 58383 | 61804 | 266989 | 383839 | 496245 | 103757 | 86295 | 19140 |
|  |  | 3rd | 7 | 2 | 2 | 7 | 15 | 12 | 7 | 28 | 23 | 251 | 8310 | 66038 | 37812 |
| **50~59** | AZ | 1st | 16037 | 12269 | 3815 | 669 | 292 | 498 | 742 | 478 | 512 | 318 | 308 | 38 | 30 |
|  |  | 2nd | 137013 | 44293 | 2582 | 1705 | 1465 | 8924 | 26290 | 14542 | 12045 | 7607 | 2815 | 1049 | 781 |
|  |  | 3rd | 2 | 5 | 3 | 7 | 5 | 7 | 15 | 12 | 6 | 7 | 4 | 6 | 3 |
|  | P | 1st | 1246018 | 24211 | 22534 | 18810 | 4289 | 9302 | 7787 | 12612 | 10822 | 11227 | 10899 | 8648 | 7549 |
|  |  | 2nd | 13746 | 214510 | 693681 | 1004807 | 399905 | 1371747 | 895782 | 40175 | 28168 | 17167 | 13787 | 14599 | 12124 |
|  |  | 3rd | 32 | 53 | 42 | 62 | 41 | 64 | 111 | 1290 | 3253 | 3650 | 19778 | 36988 | 55681 |
|  | J | 1st | 4845 | 15078 | 12776 | 13319 | 2646 | 4665 | 2291 | 1949 | 1544 | 1397 | 637 | 553 | 359 |
|  |  | 3rd | 0 | 1 | 0 | 2 | 0 | 3 | 3 | 3 | 1 | 3 | 70 | 656 | 1021 |
|  | M | 1st | 42457 | 5144 | 12528 | 15830 | 9639 | 33648 | 21772 | 77602 | 5936 | 5706 | 5161 | 5172 | 3426 |
|  |  | 2nd | 1500 | 29469 | 548457 | 92806 | 129994 | 955987 | 44563 | 16262 | 12646 | 32206 | 22633 | 74507 | 12349 |
|  |  | 3rd | 9 | 9 | 10 | 9 | 11 | 8 | 16 | 28 | 38 | 294 | 4206 | 26427 | 17030 |
| **60~64** | AZ | 1st | 35927 | 13031 | 1093 | 268 | 85 | 213 | 300 | 219 | 217 | 117 | 121 | 25 | 8 |
|  |  | 2nd | 1691639 | 1221011 | 20027 | 4303 | 1702 | 27005 | 92815 | 52749 | 31687 | 13245 | 2369 | 1204 | 839 |
|  |  | 3rd | 3 | 1 | 0 | 2 | 3 | 1 | 1 | 2 | 1 | 3 | 2 | 1 | 2 |
|  | P | 1st | 8920 | 6806 | 6689 | 5228 | 1277 | 2880 | 2372 | 3998 | 3616 | 4038 | 4064 | 3413 | 3231 |
|  |  | 2nd | 2058 | 12364 | 34875 | 29772 | 7509 | 10183 | 12223 | 5888 | 6213 | 4060 | 3792 | 4278 | 3990 |
|  |  | 3rd | 11 | 24 | 23 | 15 | 20 | 41 | 42 | 227 | 532 | 1169 | 28478 | 28197 | 31021 |
|  | J | 1st | 778 | 2960 | 2449 | 2388 | 560 | 754 | 356 | 345 | 252 | 261 | 154 | 113 | 103 |
|  |  | 3rd | 1 | 0 | 0 | 0 | 0 | 1 | 0 | 0 | 0 | 3 | 46 | 359 | 421 |
|  | M | 1st | 568 | 1211 | 3266 | 3893 | 2280 | 9183 | 6192 | 22656 | 1731 | 1799 | 1634 | 1643 | 1204 |
|  |  | 2nd | 186 | 1976 | 27306 | 1964 | 1182 | 6241 | 2163 | 3309 | 2696 | 8678 | 6235 | 21113 | 3388 |
|  |  | 3rd | 4 | 3 | 7 | 7 | 5 | 9 | 8 | 7 | 13 | 174 | 1672 | 12103 | 9768 |
| **65~69** | AZ | 1st | 18323 | 6349 | 590 | 170 | 57 | 135 | 196 | 180 | 118 | 98 | 76 | 20 | 9 |
|  |  | 2nd | 568789 | 562208 | 10220 | 2426 | 898 | 12974 | 45843 | 26003 | 16303 | 6826 | 1379 | 886 | 651 |
|  |  | 3rd | 2 | 3 | 0 | 1 | 0 | 2 | 1 | 1 | 2 | 2 | 0 | 0 | 0 |
|  | P | 1st | 4730 | 3408 | 3524 | 2743 | 766 | 1667 | 1362 | 2200 | 2162 | 2358 | 2571 | 2188 | 1987 |
|  |  | 2nd | 1066 | 3890 | 17170 | 18722 | 3905 | 5784 | 6427 | 3128 | 3411 | 2391 | 2129 | 2414 | 2431 |
|  |  | 3rd | 9 | 14 | 13 | 13 | 21 | 73 | 72 | 130 | 304 | 563 | 27131 | 20838 | 20225 |
|  | J | 1st | 335 | 1081 | 1026 | 946 | 201 | 312 | 153 | 165 | 88 | 97 | 51 | 48 | 45 |
|  |  | 3rd | 0 | 0 | 0 | 2 | 0 | 0 | 1 | 0 | 3 | 2 | 15 | 203 | 261 |
|  | M | 1st | 242 | 513 | 1506 | 1687 | 1030 | 4406 | 2960 | 11207 | 930 | 944 | 844 | 877 | 614 |
|  |  | 2nd | 62 | 856 | 13380 | 1096 | 601 | 3657 | 938 | 1523 | 1226 | 4177 | 2978 | 10403 | 1611 |
|  |  | 3rd | 2 | 7 | 8 | 4 | 9 | 3 | 17 | 15 | 33 | 148 | 877 | 5191 | 5456 |
| **70~74** | AZ | 1st | 10101 | 3610 | 375 | 105 | 31 | 97 | 136 | 107 | 89 | 64 | 37 | 23 | 13 |
|  |  | 2nd | 311020 | 303763 | 5730 | 1534 | 552 | 7907 | 25926 | 14226 | 9044 | 4031 | 891 | 517 | 424 |
|  |  | 3rd | 1 | 0 | 1 | 0 | 0 | 0 | 0 | 0 | 2 | 0 | 0 | 0 | 0 |
|  | P | 1st | 3021 | 2096 | 2079 | 1680 | 553 | 1191 | 1021 | 1595 | 1417 | 1691 | 1776 | 1559 | 1456 |
|  |  | 2nd | 677 | 2393 | 11202 | 12966 | 2483 | 3842 | 4093 | 1901 | 2144 | 1738 | 1598 | 1619 | 1717 |
|  |  | 3rd | 3 | 5 | 6 | 12 | 14 | 49 | 34 | 72 | 180 | 319 | 25317 | 16543 | 14279 |
|  | J | 1st | 122 | 444 | 458 | 394 | 77 | 107 | 40 | 60 | 47 | 42 | 25 | 19 | 19 |
|  |  | 3rd | 0 | 0 | 0 | 0 | 0 | 1 | 0 | 0 | 0 | 0 | 9 | 114 | 126 |
|  | M | 1st | 148 | 238 | 760 | 883 | 538 | 2356 | 1560 | 6273 | 529 | 421 | 385 | 423 | 297 |
|  |  | 2nd | 42 | 493 | 8483 | 741 | 353 | 2368 | 488 | 768 | 613 | 2341 | 1557 | 5764 | 905 |
|  |  | 3rd | 13 | 8 | 9 | 3 | 1 | 2 | 4 | 3 | 18 | 66 | 639 | 2800 | 3716 |
| **75~79** | AZ | 1st | 941 | 543 | 168 | 81 | 20 | 79 | 106 | 123 | 73 | 71 | 41 | 28 | 14 |
|  |  | 2nd | 1611 | 2196 | 561 | 256 | 62 | 410 | 1331 | 999 | 758 | 604 | 225 | 233 | 140 |
|  |  | 3rd | 0 | 0 | 0 | 0 | 0 | 0 | 0 | 0 | 0 | 0 | 0 | 0 | 0 |
|  | P | 1st | 2601 | 2696 | 2037 | 1666 | 608 | 1027 | 734 | 913 | 1034 | 1203 | 1125 | 1058 | 1102 |
|  |  | 2nd | 784 | 1332 | 2630 | 4839 | 1368 | 3699 | 4234 | 2158 | 2217 | 1944 | 1099 | 1215 | 1290 |
|  |  | 3rd | 4 | 9 | 7 | 9 | 18 | 36 | 27 | 33 | 212 | 15559 | 51838 | 89052 | 156045 |
|  | J | 1st | 77 | 172 | 132 | 131 | 37 | 62 | 15 | 36 | 20 | 12 | 9 | 8 | 14 |
|  |  | 3rd | 0 | 0 | 0 | 0 | 0 | 0 | 1 | 0 | 0 | 0 | 1 | 10 | 16 |
|  | M | 1st | 14 | 26 | 122 | 145 | 129 | 730 | 540 | 2826 | 240 | 244 | 187 | 198 | 162 |
|  |  | 2nd | 3 | 9 | 103 | 24 | 22 | 79 | 57 | 104 | 123 | 678 | 538 | 2548 | 364 |
|  |  | 3rd | 2 | 7 | 3 | 4 | 3 | 4 | 3 | 4 | 10 | 33 | 39 | 117 | 153 |
| **≥80** | AZ | 1st | 4730 | 2170 | 780 | 329 | 69 | 470 | 557 | 458 | 335 | 254 | 192 | 124 | 36 |
|  |  | 2nd | 5493 | 5628 | 2679 | 1198 | 277 | 2195 | 5977 | 4573 | 3568 | 2795 | 1158 | 1110 | 698 |
|  |  | 3rd | 0 | 0 | 0 | 0 | 0 | 0 | 1 | 0 | 0 | 0 | 0 | 2 | 0 |
|  | P | 1st | 4111 | 4332 | 3341 | 2792 | 980 | 1688 | 1264 | 1680 | 1765 | 2025 | 2211 | 2223 | 2608 |
|  |  | 2nd | 1068 | 1809 | 4052 | 7372 | 2238 | 5446 | 6842 | 3492 | 3741 | 3181 | 1941 | 2022 | 2279 |
|  |  | 3rd | 7 | 2 | 2 | 5 | 6 | 17 | 19 | 18 | 296 | 24402 | 67546 | 132147 | 223148 |
|  | J | 1st | 286 | 678 | 442 | 435 | 76 | 218 | 43 | 85 | 48 | 29 | 14 | 14 | 51 |
|  |  | 3rd | 0 | 0 | 0 | 1 | 0 | 0 | 0 | 0 | 0 | 0 | 0 | 5 | 10 |
|  | M | 1st | 23 | 32 | 137 | 238 | 196 | 1072 | 829 | 4895 | 400 | 369 | 296 | 335 | 371 |
|  |  | 2nd | 1 | 3 | 121 | 41 | 48 | 115 | 72 | 127 | 187 | 1026 | 770 | 4414 | 634 |
|  |  | 3rd | 3 | 2 | 4 | 2 | 9 | 1 | 1 | 0 | 6 | 19 | 31 | 123 | 277 |

| **Age** | **Vaccine** | **Dose** | **27-Nov-21** | **4-Dec-21** | **11-Dec-21** | **18-Dec-21** | **25-Dec-21** | **1-Jan-22** | **8-Jan-22** | **15-Jan-22** | **22-Jan-22** | **29-Jan-22** | **5-Feb-22** | **12-Feb-22** |
| --- | --- | --- | --- | --- | --- | --- | --- | --- | --- | --- | --- | --- | --- | --- |
| **10~14** | AZ | 1st | 0 | 1 | 0 | 0 | 0 | 0 | 0 | 0 | 0 | 0 | 0 | 0 |
|  |  | 2nd | 0 | 1 | 0 | 0 | 0 | 1 | 0 | 0 | 0 | 0 | 0 | 0 |
|  |  | 3rd | 0 | 0 | 0 | 0 | 0 | 0 | 0 | 0 | 0 | 0 | 0 | 0 |
|  | P | 1st | 174736 | 60342 | 109469 | 240464 | 233857 | 154363 | 51190 | 39168 | 28780 | 24347 | 15525 | 16055 |
|  |  | 2nd | 216418 | 275517 | 165304 | 182541 | 83043 | 107501 | 200350 | 199163 | 135228 | 69305 | 39933 | 36189 |
|  |  | 3rd | 5 | 8 | 11 | 31 | 33 | 13 | 29 | 51 | 26 | 12 | 8 | 9 |
|  | J | 1st | 0 | 0 | 0 | 0 | 0 | 0 | 0 | 0 | 0 | 0 | 0 | 0 |
|  |  | 3rd | 0 | 0 | 0 | 0 | 0 | 0 | 0 | 0 | 0 | 0 | 0 | 0 |
|  | M | 1st | 0 | 0 | 2 | 1 | 0 | 1 | 1 | 0 | 0 | 0 | 0 | 0 |
|  |  | 2nd | 0 | 0 | 2 | 0 | 0 | 0 | 1 | 0 | 1 | 0 | 0 | 0 |
|  |  | 3rd | 0 | 0 | 1 | 1 | 1 | 0 | 0 | 0 | 0 | 0 | 0 | 0 |
| **15~19** | AZ | 1st | 4 | 1 | 1 | 0 | 0 | 0 | 1 | 0 | 0 | 0 | 0 | 0 |
|  |  | 2nd | 38 | 31 | 12 | 15 | 6 | 1 | 1 | 0 | 0 | 0 | 0 | 2 |
|  |  | 3rd | 0 | 1 | 0 | 0 | 1 | 2 | 1 | 0 | 1 | 0 | 0 | 0 |
|  | P | 1st | 2966 | 2674 | 3633 | 5966 | 6821 | 3531 | 5057 | 3253 | 2059 | 1642 | 875 | 991 |
|  |  | 2nd | 5379 | 4397 | 4734 | 5587 | 4516 | 4381 | 15660 | 20774 | 14353 | 7187 | 3567 | 3174 |
|  |  | 3rd | 258 | 373 | 1473 | 79495 | 115077 | 72217 | 25637 | 30072 | 25710 | 33320 | 30146 | 49620 |
|  | J | 1st | 1 | 0 | 0 | 1 | 0 | 0 | 0 | 0 | 1 | 0 | 2 | 0 |
|  |  | 3rd | 0 | 1 | 1 | 1 | 0 | 0 | 0 | 0 | 0 | 0 | 0 | 0 |
|  | M | 1st | 10 | 5 | 2 | 5 | 1 | 0 | 0 | 1 | 0 | 0 | 0 | 0 |
|  |  | 2nd | 6 | 5 | 9 | 9 | 5 | 4 | 2 | 0 | 0 | 0 | 0 | 2 |
|  |  | 3rd | 28 | 33 | 76 | 818 | 1090 | 1815 | 167 | 300 | 516 | 1069 | 571 | 921 |
| **20~29** | AZ | 1st | 9 | 4 | 3 | 2 | 0 | 0 | 1 | 2 | 0 | 0 | 0 | 0 |
|  |  | 2nd | 84 | 43 | 20 | 20 | 19 | 7 | 5 | 8 | 2 | 2 | 1 | 3 |
|  |  | 3rd | 6 | 4 | 5 | 6 | 7 | 11 | 21 | 16 | 35 | 14 | 12 | 12 |
|  | P | 1st | 25950 | 27749 | 45539 | 67573 | 63591 | 32771 | 20879 | 15287 | 9710 | 8242 | 4414 | 5304 |
|  |  | 2nd | 58868 | 48393 | 53980 | 53259 | 39358 | 49785 | 65997 | 61943 | 33323 | 24024 | 14057 | 13300 |
|  |  | 3rd | 32381 | 35155 | 81413 | 211031 | 270265 | 325663 | 330415 | 327010 | 280526 | 321701 | 149144 | 186617 |
|  | J | 1st | 11 | 9 | 12 | 6 | 6 | 5 | 2 | 5 | 8 | 1 | 2 | 0 |
|  |  | 3rd | 6 | 14 | 6 | 2 | 3 | 1 | 3 | 3 | 2 | 2 | 1 | 2 |
|  | M | 1st | 28 | 33 | 63 | 80 | 58 | 34 | 23 | 10 | 12 | 15 | 4 | 23 |
|  |  | 2nd | 107 | 94 | 90 | 179 | 84 | 65 | 49 | 47 | 39 | 38 | 22 | 24 |
|  |  | 3rd | 1879 | 4358 | 9553 | 26290 | 20554 | 23792 | 21877 | 41594 | 66582 | 122456 | 57677 | 71581 |
| **30~39** | AZ | 1st | 36 | 25 | 5 | 3 | 1 | 2 | 0 | 2 | 0 | 0 | 0 | 0 |
|  |  | 2nd | 214 | 123 | 58 | 51 | 32 | 19 | 7 | 4 | 0 | 3 | 1 | 0 |
|  |  | 3rd | 11 | 4 | 5 | 5 | 9 | 15 | 28 | 13 | 9 | 7 | 2 | 3 |
|  | P | 1st | 16417 | 19731 | 33065 | 58691 | 54720 | 31055 | 26225 | 19205 | 11820 | 9902 | 5157 | 6311 |
|  |  | 2nd | 29249 | 24440 | 26158 | 25875 | 22259 | 32392 | 61076 | 56894 | 32459 | 26857 | 16929 | 15154 |
|  |  | 3rd | 49582 | 47714 | 68133 | 218446 | 185152 | 199858 | 228368 | 269452 | 255507 | 280379 | 132458 | 160778 |
|  | J | 1st | 626 | 566 | 843 | 908 | 419 | 256 | 267 | 187 | 172 | 130 | 46 | 80 |
|  |  | 3rd | 1493 | 915 | 1097 | 1197 | 605 | 288 | 242 | 201 | 155 | 135 | 44 | 96 |
|  | M | 1st | 6565 | 6485 | 10265 | 11597 | 5199 | 2495 | 2789 | 1495 | 811 | 717 | 443 | 555 |
|  |  | 2nd | 15493 | 13802 | 15216 | 16061 | 10617 | 8793 | 11940 | 11158 | 5854 | 3477 | 2488 | 2215 |
|  |  | 3rd | 96057 | 59417 | 56469 | 110898 | 57177 | 39369 | 29221 | 43886 | 66250 | 110935 | 54809 | 66534 |
| **40~49** | AZ | 1st | 15 | 12 | 4 | 1 | 1 | 2 | 0 | 0 | 1 | 0 | 0 | 0 |
|  |  | 2nd | 214 | 131 | 35 | 52 | 26 | 12 | 2 | 3 | 3 | 1 | 0 | 1 |
|  |  | 3rd | 6 | 6 | 9 | 10 | 14 | 34 | 36 | 15 | 11 | 5 | 2 | 4 |
|  | P | 1st | 9772 | 12288 | 20339 | 42009 | 38313 | 21341 | 17453 | 12722 | 7427 | 5675 | 2811 | 3499 |
|  |  | 2nd | 19936 | 17022 | 16990 | 17433 | 15118 | 21022 | 43666 | 40329 | 22342 | 17964 | 11460 | 9466 |
|  |  | 3rd | 63741 | 57264 | 94995 | 421494 | 347533 | 378634 | 393363 | 473702 | 422734 | 402661 | 193146 | 240825 |
|  | J | 1st | 462 | 354 | 482 | 527 | 275 | 147 | 133 | 132 | 90 | 70 | 18 | 43 |
|  |  | 3rd | 1075 | 808 | 733 | 726 | 301 | 169 | 179 | 157 | 162 | 163 | 41 | 91 |
|  | M | 1st | 4392 | 4070 | 6272 | 7562 | 3495 | 1494 | 1838 | 1018 | 509 | 419 | 240 | 320 |
|  |  | 2nd | 12171 | 10264 | 11076 | 12554 | 8316 | 6628 | 9408 | 9020 | 4637 | 2676 | 1822 | 1505 |
|  |  | 3rd | 38019 | 22929 | 22992 | 51286 | 35072 | 37896 | 37143 | 81838 | 121425 | 179725 | 91594 | 108916 |
| **50~59** | AZ | 1st | 11 | 16 | 1 | 1 | 1 | 2 | 1 | 1 | 0 | 0 | 0 | 0 |
|  |  | 2nd | 575 | 667 | 141 | 133 | 162 | 123 | 5 | 2 | 1 | 2 | 0 | 0 |
|  |  | 3rd | 6 | 10 | 9 | 11 | 8 | 34 | 35 | 8 | 16 | 3 | 0 | 1 |
|  | P | 1st | 6175 | 7225 | 11021 | 26718 | 26085 | 15104 | 11837 | 9666 | 5572 | 4086 | 2017 | 2639 |
|  |  | 2nd | 11549 | 10396 | 10082 | 10829 | 9726 | 12097 | 27983 | 27481 | 15686 | 12458 | 8510 | 6907 |
|  |  | 3rd | 89041 | 68600 | 119848 | 674340 | 661743 | 812042 | 722544 | 555218 | 339326 | 262946 | 122614 | 169653 |
|  | J | 1st | 297 | 227 | 220 | 240 | 118 | 59 | 69 | 81 | 55 | 47 | 12 | 23 |
|  |  | 3rd | 776 | 620 | 497 | 428 | 253 | 142 | 136 | 136 | 142 | 112 | 42 | 75 |
|  | M | 1st | 3103 | 2635 | 3368 | 4176 | 2007 | 865 | 973 | 667 | 320 | 277 | 141 | 173 |
|  |  | 2nd | 7490 | 5963 | 6241 | 6286 | 4763 | 4040 | 4888 | 4945 | 2571 | 1495 | 960 | 873 |
|  |  | 3rd | 20698 | 18313 | 34129 | 304645 | 239654 | 392292 | 261479 | 189347 | 124922 | 115490 | 58831 | 89533 |
| **60~64** | AZ | 1st | 6 | 8 | 0 | 0 | 0 | 0 | 0 | 0 | 0 | 0 | 0 | 0 |
|  |  | 2nd | 680 | 521 | 180 | 170 | 112 | 69 | 2 | 0 | 0 | 0 | 0 | 0 |
|  |  | 3rd | 1 | 2 | 5 | 8 | 9 | 17 | 8 | 5 | 9 | 2 | 1 | 1 |
|  | P | 1st | 2719 | 3237 | 4613 | 11057 | 10609 | 6391 | 5030 | 3913 | 2144 | 1610 | 781 | 1079 |
|  |  | 2nd | 4149 | 4030 | 4357 | 4587 | 4390 | 5162 | 11539 | 11183 | 6772 | 5180 | 3474 | 2763 |
|  |  | 3rd | 61620 | 65212 | 177949 | 308541 | 254639 | 142373 | 105019 | 72436 | 44719 | 34799 | 15513 | 25400 |
|  | J | 1st | 94 | 53 | 50 | 69 | 32 | 9 | 17 | 22 | 10 | 10 | 3 | 6 |
|  |  | 3rd | 326 | 204 | 152 | 178 | 58 | 26 | 33 | 52 | 31 | 33 | 9 | 18 |
|  | M | 1st | 1070 | 825 | 1145 | 1344 | 631 | 306 | 344 | 246 | 98 | 79 | 40 | 51 |
|  |  | 2nd | 2187 | 1695 | 1807 | 1674 | 1219 | 1119 | 1488 | 1471 | 797 | 448 | 319 | 300 |
|  |  | 3rd | 14874 | 29690 | 252388 | 677941 | 535832 | 364715 | 146655 | 85547 | 50783 | 39115 | 17079 | 27454 |
| **65~69** | AZ | 1st | 9 | 8 | 1 | 1 | 0 | 0 | 0 | 0 | 0 | 0 | 0 | 0 |
|  |  | 2nd | 506 | 408 | 123 | 104 | 55 | 43 | 0 | 0 | 0 | 0 | 0 | 0 |
|  |  | 3rd | 1 | 2 | 3 | 11 | 6 | 10 | 5 | 1 | 0 | 0 | 0 | 0 |
|  | P | 1st | 1865 | 2138 | 3139 | 6628 | 6064 | 3635 | 3116 | 2457 | 1347 | 938 | 460 | 684 |
|  |  | 2nd | 2664 | 2688 | 2751 | 3162 | 2822 | 3482 | 7219 | 6873 | 4245 | 3224 | 2106 | 1794 |
|  |  | 3rd | 44128 | 49653 | 158600 | 247715 | 152362 | 73957 | 37124 | 24991 | 16341 | 12711 | 5548 | 10388 |
|  | J | 1st | 50 | 32 | 34 | 28 | 14 | 9 | 11 | 13 | 7 | 11 | 4 | 4 |
|  |  | 3rd | 182 | 97 | 72 | 69 | 21 | 14 | 20 | 18 | 14 | 13 | 1 | 8 |
|  | M | 1st | 511 | 490 | 606 | 659 | 326 | 139 | 156 | 105 | 52 | 39 | 20 | 31 |
|  |  | 2nd | 1130 | 903 | 975 | 808 | 607 | 564 | 790 | 762 | 422 | 273 | 136 | 135 |
|  |  | 3rd | 11344 | 31304 | 310026 | 740803 | 333140 | 177267 | 84926 | 47205 | 27803 | 20483 | 8697 | 14827 |
| **70~74** | AZ | 1st | 5 | 7 | 0 | 0 | 0 | 0 | 0 | 1 | 0 | 0 | 0 | 0 |
|  |  | 2nd | 384 | 288 | 74 | 74 | 41 | 15 | 0 | 0 | 0 | 0 | 0 | 0 |
|  |  | 3rd | 1 | 0 | 0 | 7 | 0 | 4 | 0 | 0 | 0 | 1 | 0 | 0 |
|  | P | 1st | 1429 | 1714 | 2196 | 4105 | 3690 | 2313 | 1771 | 1439 | 806 | 528 | 294 | 448 |
|  |  | 2nd | 1905 | 1866 | 2080 | 2250 | 2274 | 2386 | 4257 | 3940 | 2469 | 1805 | 1189 | 1112 |
|  |  | 3rd | 26417 | 32805 | 119709 | 161369 | 87230 | 41121 | 20028 | 14009 | 9285 | 6817 | 2919 | 5898 |
|  | J | 1st | 17 | 15 | 9 | 18 | 3 | 1 | 3 | 4 | 2 | 3 | 1 | 1 |
|  |  | 3rd | 84 | 51 | 32 | 26 | 14 | 4 | 6 | 3 | 5 | 3 | 2 | 4 |
|  | M | 1st | 249 | 257 | 285 | 276 | 150 | 65 | 55 | 42 | 22 | 13 | 10 | 10 |
|  |  | 2nd | 547 | 403 | 469 | 373 | 313 | 300 | 344 | 296 | 194 | 124 | 63 | 61 |
|  |  | 3rd | 9194 | 28335 | 298426 | 564005 | 200026 | 99205 | 43792 | 24196 | 14619 | 10089 | 4483 | 7937 |
| **75~79** | AZ | 1st | 8 | 13 | 1 | 0 | 0 | 0 | 0 | 0 | 0 | 0 | 0 | 0 |
|  |  | 2nd | 116 | 137 | 17 | 17 | 14 | 0 | 0 | 0 | 0 | 0 | 0 | 0 |
|  |  | 3rd | 0 | 0 | 0 | 0 | 1 | 0 | 0 | 1 | 0 | 0 | 0 | 0 |
|  | P | 1st | 1222 | 1241 | 1867 | 2927 | 2697 | 1553 | 1169 | 1013 | 673 | 506 | 218 | 370 |
|  |  | 2nd | 1330 | 1272 | 1523 | 1558 | 1528 | 1854 | 2838 | 2801 | 1752 | 1298 | 810 | 861 |
|  |  | 3rd | 198972 | 264804 | 298887 | 192409 | 82234 | 39829 | 16221 | 11327 | 7307 | 5198 | 2028 | 3653 |
|  | J | 1st | 16 | 9 | 9 | 6 | 4 | 4 | 0 | 3 | 0 | 1 | 0 | 0 |
|  |  | 3rd | 5 | 5 | 2 | 4 | 4 | 2 | 2 | 3 | 1 | 1 | 0 | 0 |
|  | M | 1st | 170 | 117 | 124 | 108 | 53 | 37 | 27 | 17 | 8 | 12 | 5 | 7 |
|  |  | 2nd | 276 | 212 | 198 | 179 | 174 | 125 | 154 | 132 | 72 | 55 | 24 | 26 |
|  |  | 3rd | 1059 | 1458 | 1875 | 1741 | 841 | 670 | 7589 | 4525 | 2802 | 2080 | 892 | 2030 |
| **≥80** | AZ | 1st | 69 | 69 | 12 | 1 | 0 | 0 | 0 | 0 | 0 | 0 | 0 | 0 |
|  |  | 2nd | 607 | 558 | 48 | 71 | 36 | 10 | 0 | 0 | 0 | 0 | 0 | 0 |
|  |  | 3rd | 0 | 1 | 0 | 0 | 0 | 0 | 0 | 0 | 0 | 0 | 0 | 0 |
|  | P | 1st | 3394 | 3069 | 4728 | 6747 | 6320 | 3687 | 2715 | 2632 | 1878 | 1508 | 568 | 1092 |
|  |  | 2nd | 2763 | 2756 | 3277 | 3862 | 3604 | 4414 | 6667 | 6695 | 4656 | 3315 | 1831 | 2184 |
|  |  | 3rd | 274631 | 283949 | 289669 | 190702 | 85662 | 45592 | 26309 | 21105 | 15686 | 12386 | 3590 | 7361 |
|  | J | 1st | 81 | 29 | 20 | 19 | 23 | 13 | 2 | 6 | 5 | 5 | 0 | 5 |
|  |  | 3rd | 21 | 9 | 4 | 1 | 2 | 0 | 0 | 1 | 0 | 1 | 0 | 1 |
|  | M | 1st | 400 | 223 | 191 | 173 | 71 | 88 | 44 | 34 | 22 | 17 | 7 | 17 |
|  |  | 2nd | 404 | 312 | 367 | 333 | 396 | 264 | 239 | 204 | 96 | 119 | 37 | 49 |
|  |  | 3rd | 2906 | 2385 | 2612 | 2346 | 1315 | 1052 | 629 | 779 | 770 | 863 | 446 | 1713 |

| **Age** | **Vaccine** | **Dose** | **19-Feb-22** | **26-Feb-22** | **5-Mar-22** | **12-Mar-22** |
| --- | --- | --- | --- | --- | --- | --- |
| **10~14** | AZ | 1st | 0 | 0 | 0 | 0 |
|  |  | 2nd | 0 | 0 | 0 | 0 |
|  |  | 3rd | 0 | 0 | 0 | 0 |
|  | P | 1st | 14108 | 14065 | 5604 | 2978 |
|  |  | 2nd | 27954 | 20469 | 12370 | 9452 |
|  |  | 3rd | 6 | 5 | 0 | 0 |
|  | J | 1st | 0 | 0 | 0 | 0 |
|  |  | 3rd | 0 | 0 | 0 | 0 |
|  | M | 1st | 0 | 0 | 0 | 0 |
|  |  | 2nd | 0 | 0 | 0 | 0 |
|  |  | 3rd | 0 | 0 | 0 | 0 |
| **15~19** | AZ | 1st | 0 | 0 | 0 | 0 |
|  |  | 2nd | 0 | 0 | 0 | 0 |
|  |  | 3rd | 0 | 0 | 0 | 0 |
|  | P | 1st | 687 | 518 | 182 | 111 |
|  |  | 2nd | 2186 | 1366 | 755 | 496 |
|  |  | 3rd | 40457 | 43352 | 16364 | 13596 |
|  | J | 1st | 0 | 0 | 0 | 0 |
|  |  | 3rd | 0 | 0 | 0 | 0 |
|  | M | 1st | 0 | 0 | 0 | 0 |
|  |  | 2nd | 1 | 0 | 0 | 0 |
|  |  | 3rd | 662 | 523 | 200 | 125 |
| **20~29** | AZ | 1st | 0 | 0 | 0 | 0 |
|  |  | 2nd | 0 | 0 | 0 | 0 |
|  |  | 3rd | 2 | 0 | 0 | 0 |
|  | P | 1st | 3513 | 2320 | 1060 | 580 |
|  |  | 2nd | 9047 | 5556 | 3648 | 2295 |
|  |  | 3rd | 162074 | 148875 | 76045 | 48963 |
|  | J | 1st | 2 | 4 | 2 | 4 |
|  |  | 3rd | 1 | 2 | 0 | 1 |
|  | M | 1st | 4 | 1 | 0 | 0 |
|  |  | 2nd | 29 | 5 | 0 | 0 |
|  |  | 3rd | 60591 | 52245 | 24924 | 17208 |
| **30~39** | AZ | 1st | 0 | 0 | 0 | 0 |
|  |  | 2nd | 0 | 0 | 0 | 0 |
|  |  | 3rd | 3 | 1 | 0 | 0 |
|  | P | 1st | 4319 | 2863 | 1341 | 744 |
|  |  | 2nd | 10283 | 5996 | 4590 | 2912 |
|  |  | 3rd | 134341 | 118508 | 61283 | 40658 |
|  | J | 1st | 46 | 40 | 12 | 9 |
|  |  | 3rd | 64 | 62 | 43 | 5 |
|  | M | 1st | 299 | 212 | 111 | 62 |
|  |  | 2nd | 1124 | 891 | 365 | 318 |
|  |  | 3rd | 54564 | 45504 | 23176 | 16358 |
| **40~49** | AZ | 1st | 0 | 0 | 0 | 0 |
|  |  | 2nd | 0 | 0 | 0 | 0 |
|  |  | 3rd | 3 | 0 | 1 | 0 |
|  | P | 1st | 2236 | 1396 | 610 | 292 |
|  |  | 2nd | 5945 | 3392 | 2646 | 1666 |
|  |  | 3rd | 190480 | 154747 | 75482 | 49708 |
|  | J | 1st | 17 | 18 | 5 | 5 |
|  |  | 3rd | 83 | 65 | 27 | 11 |
|  | M | 1st | 185 | 133 | 59 | 37 |
|  |  | 2nd | 813 | 575 | 238 | 189 |
|  |  | 3rd | 84592 | 66937 | 32748 | 22529 |
| **50~59** | AZ | 1st | 0 | 0 | 0 | 0 |
|  |  | 2nd | 0 | 0 | 0 | 0 |
|  |  | 3rd | 2 | 0 | 0 | 0 |
|  | P | 1st | 1788 | 1086 | 465 | 222 |
|  |  | 2nd | 4369 | 2498 | 2218 | 1467 |
|  |  | 3rd | 135328 | 113099 | 60338 | 39776 |
|  | J | 1st | 10 | 4 | 6 | 2 |
|  |  | 3rd | 61 | 45 | 16 | 17 |
|  | M | 1st | 115 | 98 | 42 | 18 |
|  |  | 2nd | 497 | 354 | 167 | 138 |
|  |  | 3rd | 71001 | 57503 | 29289 | 18513 |
| **60~64** | AZ | 1st | 0 | 0 | 0 | 0 |
|  |  | 2nd | 0 | 0 | 0 | 0 |
|  |  | 3rd | 1 | 0 | 0 | 0 |
|  | P | 1st | 696 | 420 | 197 | 116 |
|  |  | 2nd | 1746 | 1054 | 899 | 613 |
|  |  | 3rd | 21684 | 20827 | 10386 | 6282 |
|  | J | 1st | 6 | 0 | 3 | 0 |
|  |  | 3rd | 17 | 21 | 3 | 3 |
|  | M | 1st | 23 | 37 | 11 | 7 |
|  |  | 2nd | 170 | 104 | 55 | 49 |
|  |  | 3rd | 22867 | 20346 | 9527 | 4363 |
| **65~69** | AZ | 1st | 0 | 0 | 0 | 0 |
|  |  | 2nd | 0 | 0 | 0 | 0 |
|  |  | 3rd | 1 | 0 | 0 | 0 |
|  | P | 1st | 448 | 312 | 143 | 108 |
|  |  | 2nd | 1074 | 646 | 651 | 396 |
|  |  | 3rd | 8627 | 8163 | 4316 | 2677 |
|  | J | 1st | 0 | 0 | 1 | 0 |
|  |  | 3rd | 5 | 6 | 4 | 0 |
|  | M | 1st | 12 | 11 | 3 | 8 |
|  |  | 2nd | 84 | 60 | 22 | 35 |
|  |  | 3rd | 11681 | 9565 | 4653 | 2175 |
| **70~74** | AZ | 1st | 0 | 0 | 0 | 0 |
|  |  | 2nd | 0 | 0 | 0 | 0 |
|  |  | 3rd | 0 | 0 | 0 | 0 |
|  | P | 1st | 318 | 222 | 95 | 69 |
|  |  | 2nd | 672 | 426 | 445 | 302 |
|  |  | 3rd | 4577 | 4251 | 2579 | 1709 |
|  | J | 1st | 0 | 0 | 0 | 0 |
|  |  | 3rd | 1 | 3 | 2 | 0 |
|  | M | 1st | 10 | 5 | 2 | 2 |
|  |  | 2nd | 30 | 26 | 9 | 11 |
|  |  | 3rd | 5940 | 4555 | 2332 | 1260 |
| **75~79** | AZ | 1st | 0 | 0 | 0 | 0 |
|  |  | 2nd | 0 | 0 | 0 | 0 |
|  |  | 3rd | 0 | 0 | 0 | 0 |
|  | P | 1st | 244 | 191 | 109 | 71 |
|  |  | 2nd | 548 | 340 | 346 | 222 |
|  |  | 3rd | 2914 | 2434 | 1573 | 1367 |
|  | J | 1st | 1 | 0 | 0 | 0 |
|  |  | 3rd | 2 | 1 | 1 | 0 |
|  | M | 1st | 2 | 5 | 5 | 0 |
|  |  | 2nd | 20 | 12 | 12 | 6 |
|  |  | 3rd | 1519 | 1061 | 579 | 384 |
| **≥80** | AZ | 1st | 0 | 0 | 0 | 0 |
|  |  | 2nd | 0 | 0 | 0 | 0 |
|  |  | 3rd | 0 | 0 | 0 | 0 |
|  | P | 1st | 830 | 565 | 290 | 246 |
|  |  | 2nd | 1544 | 984 | 898 | 775 |
|  |  | 3rd | 5879 | 5126 | 3349 | 3031 |
|  | J | 1st | 5 | 1 | 0 | 0 |
|  |  | 3rd | 1 | 3 | 1 | 0 |
|  | M | 1st | 6 | 13 | 5 | 1 |
|  |  | 2nd | 35 | 20 | 12 | 12 |
|  |  | 3rd | 1184 | 865 | 468 | 361 |

AZ; ChAdOx1 nCoV-19 (AZD1222, Oxford–AstraZeneca), P; BNT162b2 (tozinameran, Pfizer–BioNTech), J; Ad26.COV2.S (Johnson & Johnson), M; mRNA-1273 (elasomeran, Moderna)

On July 16, 2021, Korean Ministry of Food and Drug Safety (MFDS) approved the BNT162b2 (tozinameran, Pfizer–BioNTech), for use in individuals aged 12 years or more. On Feb 23, 2022, Korean Ministry of Food and Drug Safety (MFDS) approved the BNT162b2 (tozinameran, Pfizer–BioNTech), for use in children aged 5 to 11 years.

Reference: https://ncv.kdca.go.kr/

*More refined vaccination data of doses and manufacturers were provided by Korea Disease Control and Prevention Agency.
